# Silver‐Catalyzed Decarboxylative Coupling of Oxamic Acids with Styrenes to Synthesize *E*‐Cinnamamides: A Distinguish Reaction Pathway

**DOI:** 10.1002/open.202400513

**Published:** 2025-01-29

**Authors:** Ru‐Han A, Yong‐Wang Huo, Xiao‐Feng Wu

**Affiliations:** ^1^ Dalian National Laboratory for Clean Energy Dalian Institute of Chemical Physics Chinese Academy of Sciences Dalian 116023, Liaoning China; ^2^ Leibniz-Institut für Katalyse e. V. Albert-Einstein-Straβe 29a 18059 Rostock Germany

**Keywords:** Cinnamamide, Oxamic acid, Silver catalysis, Decarboxylation, Radical reaction

## Abstract

A silver‐catalyzed decarboxylative coupling of oxamic acids with styrenes has been developed to produce *E*‐cinnamamides. Oxamic acids act as efficient precursors for carbamoy radicals. Based on the mechanistic experiments and intermediate analysis, the proposed mechanism involves radical addition to styrenes, followed by oxidation and solvent participation, ultimately leading to the formation of cinnamamides which is different from the reported cases.

## Graphical abstract

A silver‐catalyzed decarboxylative coupling of readily available oxamic acids with styrenes to the direct synthesis of E‐cinnamamides has been developed. The reaction pathway was studied and proven to be different from known procedures.

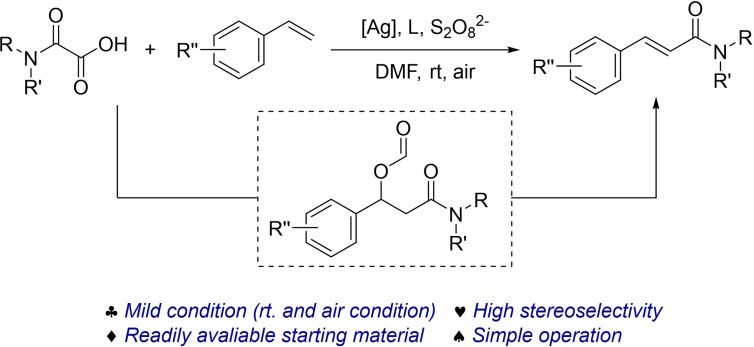

## Introduction

**Scheme 1:**
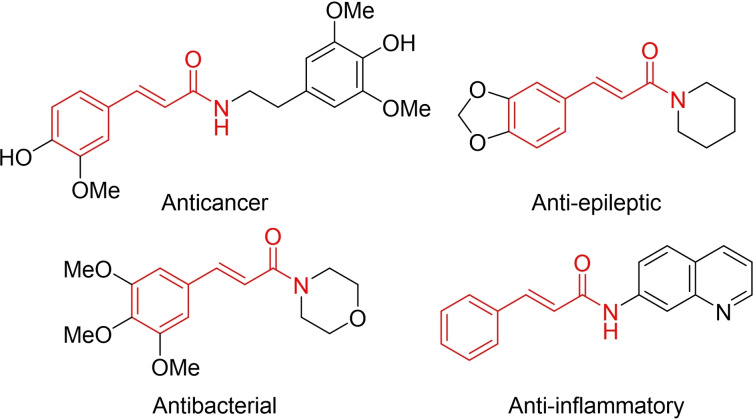
Selected biologically active cinnamamides.

Natural and synthetic compounds with a cinnamamide backbone have demonstrated valuable biological and pharmacological properties, such as anticancer,[^1^, ^2^, ^3^, ^4^, ^5^] antibacterial,[^6^, ^7^, ^8^, ^9^, ^10^] and anti‐inflammatory effects,[^11^, ^12^, ^13^, ^14^] as well as potential applications in the treatment of various neurological diseases.[^15^, ^16^, ^17^] Therefore, developing more efficient and mild methods for the preparation of cinnamamides remains an important research topic in the field of organic chemistry (Scheme 1).^[18]^

Traditional methods for synthesizing cinnamamides involve the condensation of cinnamic acids with amines in the presence of condensing/dehydrating agents, converting cinnamic acids into more reactive acyl chlorides followed by reaction with amines,[^19^, ^20^, ^21^] or the decarboxylation of cinnamic acids.^[22]^ However, these methods often require multiple steps, expensive, toxic or unstable reagents.

**Scheme 2:**
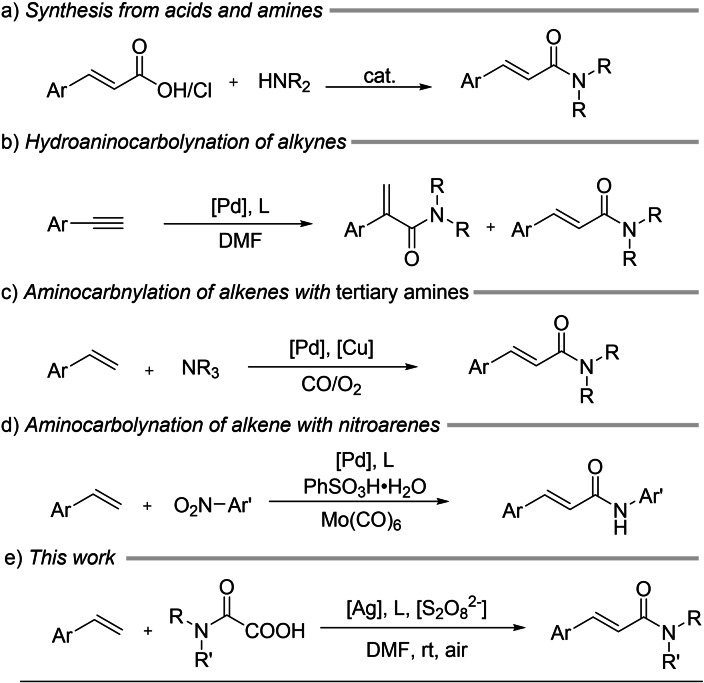
Synthesis of cinnamamides.

Considering the limitations of the aforementioned methods, new synthetic approaches are continuously being developed (Scheme 2). In 2010, Tsuji's group reported a regio‐ and stereoselective synthesis of (*E*)‐*α*,*β*‐unsaturated amides via the reaction of formamides with internal alkynes in the presence of a palladium catalyst with acyl chloride as an additive.^[23]^ In 2015, the Lei group reported the synthesis of (*E*)‐*α*,*β*‐unsaturated amides via a Pd/Cu‐catalyzed aerobic oxidative *N*‐dealkylation reaction using tertiary amines, alkenes, and CO.^[24]^ Furthermore, in 2018, our group reported a method for synthesizing cinnamamides through an aminocarbonylation reaction of alkenes with nitroarenes using Mo(CO)_6_ as a solid CO source and reductant using palladium catalysis.^[25]^

With the development of radical chemistry, carbamoyl radicals have been used as synthons, providing a new approach for the construction of amides. Oxamic acids are easily accessible, non‐toxic, and environmentally friendly compounds.^[26]^ Oxamic acids can be oxidatively decarboxylated to generate carbamoyl radicals via thermal,[^27^, ^28^, ^29^, ^30^, ^31^] photochemical,[^32^, ^33^, ^34^, ^35^, ^36^] electrochemical[^37^, ^38^, ^39^, ^40^] making them potent precursor for generating carbamoyl radicals. Unlike the decarboxylative coupling of carboxylic acids and keto acids, the decarboxylation of oxamic acid can proceed under milder conditions.^[26]^ Classical conditions typically involve decarboxylation in the presence of persulfates and heating, and silver salts can catalyze this reaction.[^41^, ^42^, ^43^, ^44^, ^45^] Herein, we wish to report our recently developed silver‐catalyzed decarboxylation of oxamic acids with styrenes to produce *E*‐cinnamamides. The reaction works at room temperature and under air atmosphere. Analysis of reaction intermediates revealed that the mechanism is not a simple radical addition to alkenes followed by oxidation and *β*‐H elimination to give the desired product which as has been reported in literature,[^46^, ^47^] but rather involves a new pathway with solvent participation.

## Results and Discussion

The research commenced by employing *N*,*N*‐dimethyloxalamic acid (**1 a**) and styrene (**2 a**) as model substrates, using (NH_4_)_2_S_2_O_8_ as the oxidant, AgNO_3_ as the catalyst, and 4,7‐diphenyl‐1,10‐phenanthroline (**L3**) as the ligand in DMF at room temperature under N_2_ atmosphere, with a reaction time of 24 hours. The results of reaction optimization were summarized in Table 1. Notably, this reaction can proceed at room temperature without the need of heating, and prolonged heating actually reduces the reaction yield (Table 1, entry 2). Screening various solvents revealed that DMF is the optimal choice, possibly because it not only functions as a solvent but also participates in the reaction. When testing the reaction's sensitivity to water by adding various amounts, we found that higher water loading led to lower yields and could even completely inhibit the reaction (Table 1, entry 6). Meanwhile, examining the effects of different oxidants on the reaction, we found that organic oxidants are ineffective at room temperature, the reaction does not proceed (Table 1, entry 9). Among peroxydisulfates with various cations, only ammonium persulfate showed optimal performance in the model reaction (Table 1, entry 7, 8). Different silver salts were evaluated as catalysts, and all resulted in lower yields compared to AgNO_3_ (Table 1, entries 11–13). Without a silver salt catalyst, the reaction does not occur, indicating its essential role (Table 1, entry 10). Ligands based on the 1,10‐phenanthroline framework are crucial for the reaction, enabling the decarboxylation of oxamic acid at room temperature (Table S2). During the screening of ligands with various substituents at different positions, we observed that ligands with aromatic groups at the 4,7‐positions yield higher reaction rates. In contrast, substituents at the 2,9‐positions (**L8**) inhibit the reaction, likely due to steric hindrance that prevents the coordination of the ligand with silver ions (Table 1, entries 14–16; for more details, see Supporting Information).

**Table 1:** Optimization of reaction conditions.^[a]^

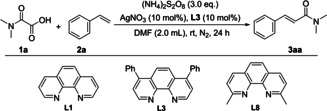		
Entry	Variations	Yield(%)^[a]^
1	None	83
2	At 80 °C	27
3	NMP instead of DMF	13
4	DMAc instead of DMF	24
5	Benzene instead of DMF	N.D.
6	DMF : H_2_O (200 : 1/100 : 1/25 : 1)	45/27/N.D.
7	Na_2_S_2_O_8_ instead of (NH_4_)_2_S_2_O_8_	33
8	K_2_S_2_O_8_ instead of (NH_4_)_2_S_2_O_8_	62
9	BPO instead of (NH_4_)_2_S_2_O_8_	N.D.
10	Without AgNO_3_	N.D.
11	AgCl instead of AgNO_3_	N.D.
12	AgTFA instead of AgNO_3_	68
13^[b]^	Ag_2_CO_3_ instead of AgNO_3_	48
14	Without L3	N.D.
15	L1 instead of L3	72
16	L8 instead of L3	N.D.
17	Air condition	85

Reaction conditions: 1a (0.6 mmol, 3.0 equiv.), 2a (0.2 mmol, 1.0 equiv.), AgNO_3_ (10 mol %), ligand (10 mol %), (NH_4_)_2_S_2_O_8_ (0.6 mmol, 3.0 equiv.), DMF (2.0 mL), N_2_, 24 h. [a] The yields were determined by GC using *n*‐hexadecane as the internal standard. [b] Ag_2_CO_3_ (5 mol %).

**Scheme 3:**
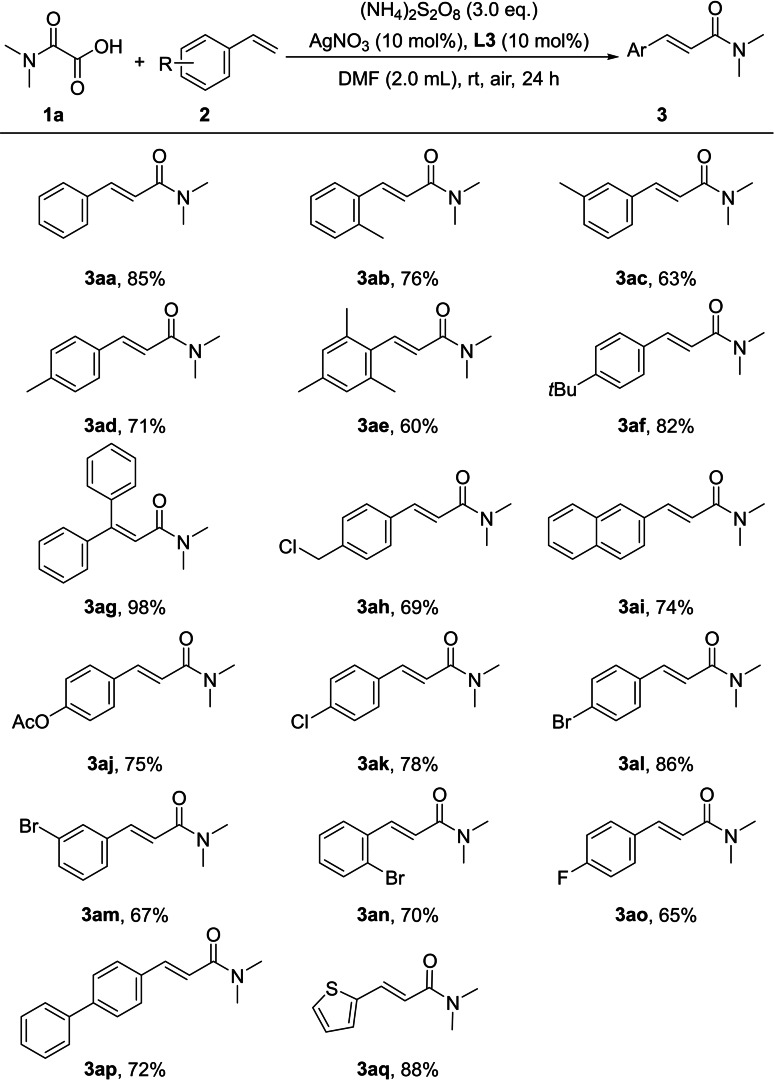
Scope of styrenes. Reaction conditions: **1 a** (0.6 mmol, 3.0 equiv.), **2** (0.2 mmol, 1.0 equiv.), AgNO_3_ (10 mol %), L3 (10 mol %), (NH_4_)_2_S_2_O_8_ (0.6 mmol, 3.0 equiv.), DMF (2.0 mL), air, 24 h.

After established the optimal reaction conditions, we conducted studies on the scope of substrates for this transformation. Initially, various derivatives of styrene were tested, yielding the desired products in moderate to good yields (Scheme 3). Substituents at the *ortho*, *meta*, or *para* positions all yielded the corresponding target products with relatively good yields. The lower yield of **3 ea** may be attributed to the increased steric hindrance from two methyl groups at the *ortho* positions. When a bulky *tert*‐butyl group was placed at the *para* position, it had a minimal impact on the reaction, still allowing for the target product **3 ef** to be obtained with a yield of 82 %. Interestingly, a phenyl group is located at the benzylic position, it not only does not inhibit the reaction but even leads to the highest yield of 98 %, as seen in the case of product **3 ag**. This indicates that the presence of a phenyl group at this position may stabilize the reaction intermediates, potentially through conjugation or resonance effects. The increased stability likely promotes product formation, which explains why **3 ag** achieves such a remarkably high yield. However, placing the phenyl group, whether positioned at the *para* position or extended in the form of a fused ring to expand the π‐system, does not significantly enhance the yield. The corresponding target products were obtained with yields of 72 % (**3 ap**) and 74 % (**3 ai**), respectively. Simply increasing π‐conjugation or placing the phenyl group at the *para* position does not lead to a dramatic improvement in reaction efficiency. Halogen groups at different positions on the aromatic ring consistently result in good yields of the target products (**3 ak**‐**3 ao**). When the phenyl ring was replaced by a thiophene ring, the target product **3 aq** was obtained with an excellent yield of 88 %. It is worth to mention that 4‐nitrostyrene, 4‐(trifluoromethyl)styrene, and 4‐vinylpyridine was also tested, but less than 10 % of the desired product was obtained even after 48 hours.

**Scheme 4:**
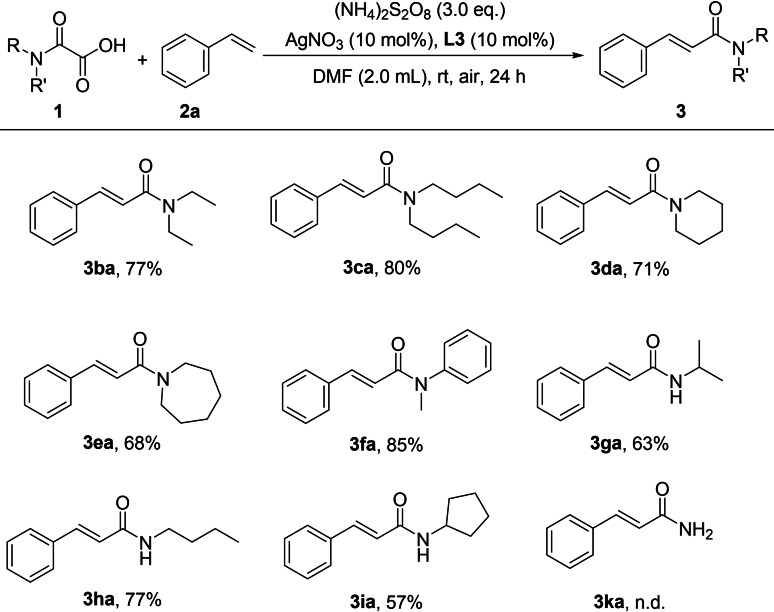
Scope of oxamic acids. Reaction conditions: **1** (0.6 mmol, 3.0 equiv.), **2 a** (0.2 mmol, 1.0 equiv.), AgNO_3_ (10 mol %), L3 (10 mol %), (NH_4_)_2_S_2_O_8_ (0.6 mmol, 3.0 equiv.), DMF (2.0 mL), air, 24 h.

Subsequently, we investigated the scope of oxamic acid derivatives with different substituents on the amino group under the standard conditions (Scheme 4). As expected, the disubstituted oxamic acids used in this set of experiments resulted in good yields for the target products **3 ba** to **3 fa**, the presence of two substituents on the oxamic acid does not hinder the reaction significantly, and the reaction can still proceed efficiently. When the amine moiety was secondary amine, the target products were obtained with good yields (**3 ga**–**3 ia**). The secondary amines are well‐tolerated under the reaction conditions. However, when the unsubstituted oxamic acid was used as the reaction substrate, the target product **3 ak** was not obtained.

**Scheme 5:**
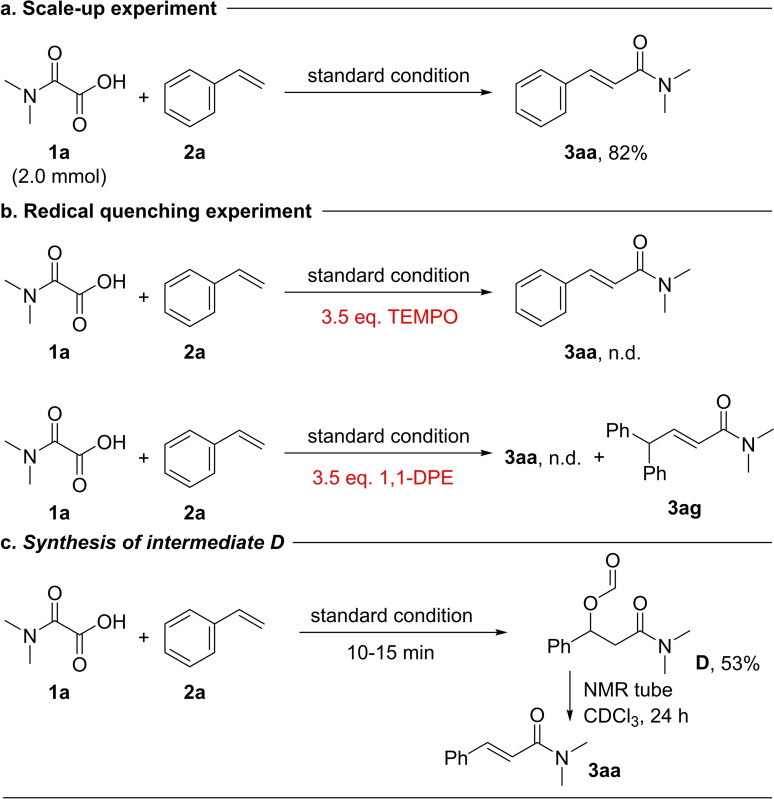
Control experiments.

Furthermore, under standard condition, we evaluated the reaction's feasibility through scale‐up experiment. As expected, the target product **3 aa** was obtained with a yield of 82 % (Scheme 5a). Then several control experiments were conducted to gain a more comprehensive understanding of the reaction. Initially, when the model reaction was performed with the inclusion of the radical trapping agent TEMPO, the desired product was undetectable by GC‐MS. Subsequently, when 1,1‐diphenylethylene (DPE) was added to the reaction, only **3 ag** was detected (Scheme 5b). This suggests that the reaction likely proceeds through a radical pathway. Finally, by reducing the reaction time, the unstable intermediate was successfully obtained, which spontaneously converts into **3 aa** in NMR tube (Scheme 5c).

**Scheme 6:**
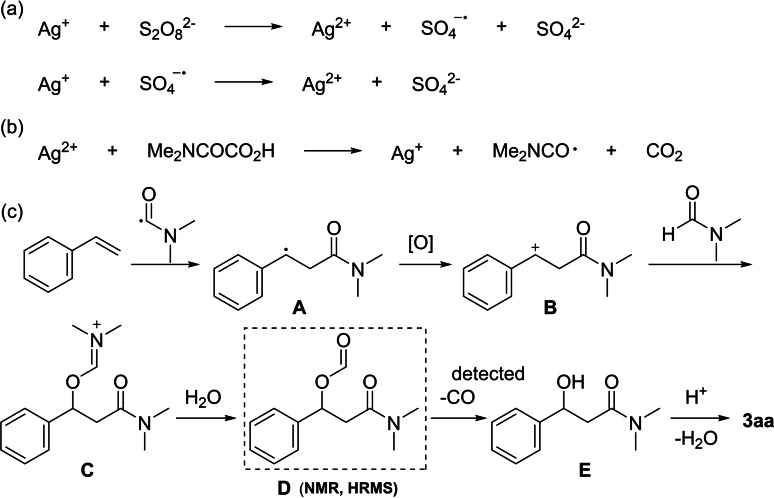
Plausible reaction mechanism.

Based on the mechanistic investigations and the relevant previous reports,[^41^, ^42^, ^43^, ^44^, ^45^, ^46^, ^47^] a tentative reaction mechanism was proposed as depicted in Scheme 6. The reaction begins with the oxidation of Ag^+^ by persulfate (S₂O₈^2−^) to generate Ag^2+^ (Scheme 6a). Oxamic acid undergoes oxidative decarboxylation to form a carbamoyl radical, the reduced silver ions in the reaction are reoxidized by persulfate to Ag^2+^, continuing to participate in the redox cycle (Scheme 6b). The carbamoyl radical then adds to the styrene, forming a new radical intermediate **A**. This intermediate is then oxidized to form a cation **B**, which undergoes nucleophilic attack by the lone pair of electrons from DMF, leading to the formation of an imine like intermediate **C**. This intermediate **C** is subsequently hydrolyzed, followed by decarbonylation and dehydration steps to resulting in the final product **3 aa** (Scheme 6c).

## Conclusions

In conclusion, we have developed a silver catalyzed method for the oxidative decarboxylation of oxamic acids and their coupling with styrenes to synthesize cinnamamides under mild conditions. This reaction proceeds efficiently at room temperature and in the presence of air. Oxamic acids, serving as carbamoyl radical precursors, are easily accessible, stable, and environmentally friendly. Through mechanistic experiments and intermediate analysis, the mechanism is proposed to involve a pathway with solvent participation which is different from reported cases. The reaction exhibits high yields, broad substrate scope, and excellent selectivity. Overall, this work presents a new efficient approach for the synthesis of *E*‐cinnamamides.

## Conflict of Interests

The authors declare no conflict of interest.

## Supporting information

As a service to our authors and readers, this journal provides supporting information supplied by the authors. Such materials are peer reviewed and may be re‐organized for online delivery, but are not copy‐edited or typeset. Technical support issues arising from supporting information (other than missing files) should be addressed to the authors.

Supporting Information

## Data Availability

The data that support the findings of this study are available in the supplementary material of this article.
