# Supplementary material for: Silver‐Catalyzed Decarboxylative Coupling of Oxamic Acids with Styrenes to Synthesize E‐Cinnamamides: A Distinguish Reaction Pathway
Source: ChemistryOpen. 2025 Jan 29;14(8):e202400513. doi: 10.1002/open.202400513 (PMC12368892; doi:10.1002/open.202400513)
Supplement: Supplementary file 1 — Supporting Information [file OPEN-14-e202400513-s001.pdf]

ChemistryOpen

Supporting Information

Silver-Catalyzed Decarboxylative Coupling of Oxamic Acids with Styrenes to Synthesize *E*-Cinnamamides: A Distinguish Reaction Pathway

Ru-Han A, Yong-Wang Huo, and Xiao-Feng Wu*

Supporting Information

Silver-Catalyzed Decarboxylative Coupling of Oxamic Acids with Styrenes to Synthesize *E*-Cinnamamides: A Distinguish Reaction Pathway

Ru-Han A[a,b], Yong-Wang Huo[a,b], Xiao-Feng Wu[a,b,*]

[a]*Dalian National Laboratory for Clean Energy, Dalian Institute of Chemical Physics, Chinese Academy of Sciences, Dalian 116023, Liaoning, China*

[b]*Leibniz-Institut für Katalyse e. V., Albert-Einstein-Straβe 29a, 18059 Rostock, Germany*

Email: xwu2020@dicp.ac.cn (X.-F. Wu)

Table of Contents

1. General information S2
2. Optimization of reaction conditions S3
3. General Procedures for Synthesis of Substrates S6
   3.1 General Procedure for the Synthesis of Oxamic acids S6
4. General Procedures........ S7
   4.1 General Procedures for S7
   4.2 Procedure for 2.0 mmol Scale Reaction S7
5. Characterization Data S8
6. Mechanism study experiment........ S14
   6.1 Redical inhibition experiment S14
   6.2 Redical capture experiment S14
   6.3 Procedure for the Synthesis of Intermediates D S15
7. References S17
8. Copies of NMR Spectra........ S18

1. General information

Unless otherwise noted, all reactions were carried out under air or nitrogen (N_2) atmosphere. All reagents were purchased from commercial suppliers and used without further purification. Column chromatography was performed on silica gel (200-300 meshes) using petroleum ether (bp. 60~90 °C), ethyl acetate, dichloromethane as eluent. All NMR spectra were recorded at ambient temperature using Bruker Avance III 400 MHz NMR spectrometers and spectral data were reported in ppm relative to tetramethylsilane (TMS) as the internal standard and $CDCl_3$ or DMSO-d_6 as solvent. All coupling constants (*J*) were reported in Hertz unite (Hz) with the following abbreviations: s = singlet, d = doublet, dd = double doublet, t = triplet, dt = double triplet, q = quatriplet, m = multiplet, br = broad. All reactions were monitored by GC-FID or NMR analysis, GC-yields were calculated using n-hexadecane as internal standard. All measurements were carried out at room temperature unless otherwise stated.

2. Optimization of reaction conditions

Table S1. Effect of solvent

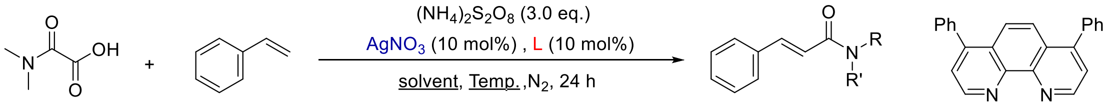

1a 2a 3aa L

Entry	Solvent	Volume (mL)	Temp. (°C)	Yield (%)[a]
1	MeCN	1.0	80	3
2	MeCN	2.0	80	3
3	MeCN	2.0	80	6
4	DCE	2.0	80	6
5	$PhCF_3$	2.0	80	10
6	THF	2.0	80	6
7	DMSO	2.0	80	8
8	DMF	2.0	80	27
9	EA	2.0	80	37
10	1,4-dioxane	2.0	80	4
11	DMF	2.0	r.t.	83
12	DMF[b]	2.0	r.t.	85
13	DMF+H_2O	2.0 (200:1)	r.t.	45
14	DMF+H_2O	2.0 (100:1)	r.t.	27
15	DMF+H_2O	2.0 (25:1)	r.t.	n.d.
16	DMAc	2.0	r.t.	24
17	NMP	2.0	r.t.	13
18	Acetone	2.0	r.t.	n.d.
19	Benzene	2.0	r.t.	n.d.
20	n-hexane	2.0	r.t.	n.d.
21	MeOH	2.0	r.t.	n.d.
22	H_2O	2.0	r.t.	n.d.

Reaction conditions: **1a** (0.6 mmol, 3.0 equiv.), **2a** (0.2 mmol, 1.0 equiv.), $AgNO_3$ (10 mol%), ligand (10 mol%), $(NH_4)_2S_2O_8$ (0.6 mmol, 3.0 equiv.), N_2, 24 h. [a] The yields were determined by GC using *n*-hexadecane as the internal standard; [b] Air atmosphere.

Table S2. Effect of ligand

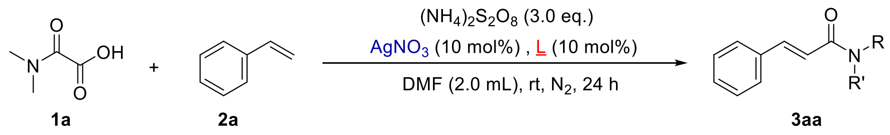

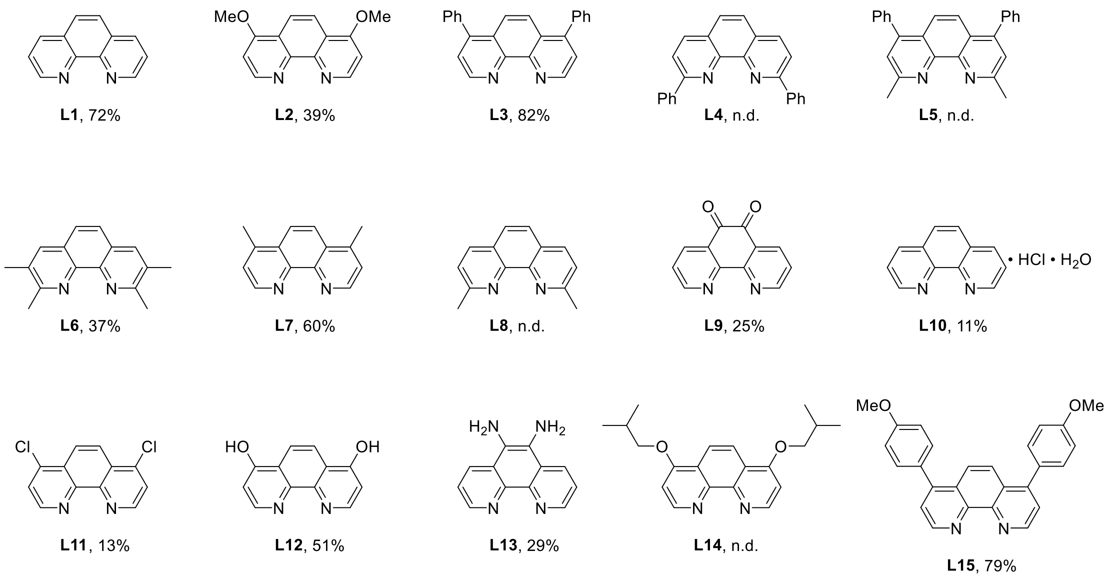

Reaction conditions: **1a** (0.6 mmol, 3.0 equiv.), **2a** (0.2 mmol, 1.0 equiv.), $AgNO_3$ (10 mol%), ligand (10 mol%), $(NH_4)_2S_2O_8$ (0.6 mmol, 3.0 equiv.), DMF (2.0 mL), N_2, 24 h. The yields were determined by GC using *n*-hexadecane as the internal standard.

Table S3. Effect of catalyst

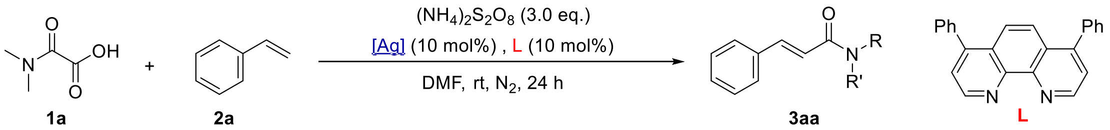

Entry	Cat.	Yield%[a]	Entry	Cat.	Yield%[a]
1	AgF	29	10	Ag_2O[b]	30
2	AgCl	n.d.	11	Ag_2CO_3	48
3	$AgClO_4$	28	12	Ag_2CO_3[b]	20
4	AgTFA	68	13	Ag_3PO_4	57
5	$AgSO_3CF_3$	51	14	Ag_3PO_4[c]	26
6	$AgSbF_6$	15	15	$AgNO_3$[b]	22
7	$AgNO_2$	35	16	$AgNO_3$[d]	77
8	$AgNO_3$	87	17	—	n.d.
9	Ag_2O	54	18	$AgBF_4$	33

Reaction conditions: **1a** (0.6 mmol, 3.0 equiv.), **2a** (0.2 mmol, 1.0 equiv.), $AgNO_3$ (10 mol%), ligand (10 mol%), $(NH_4)_2S_2O_8$ (0.6 mmol, 3.0 equiv.), DMF (2.0 mL), N_2, 24 h. [a] The yields were determined by GC using *n*-hexadecane as the internal standard; [b] 5 mol%; [c] 3.3 mol%; [d] 20 mol%.

Table S4. Effect of oxidant

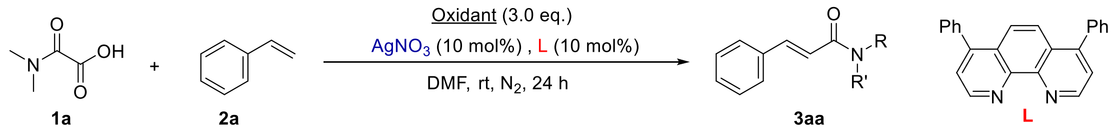

Entry	Oxidant	Yield%[a]
1	$(NH_4)_2S_2O_8$	83
2	$Na_2S_2O_8$	33
3	$K_2S_2O_8$	62
4	Oxone	3.
5	H_2O_2	n.d.
6	BPO	n.d.
7	—	n.d.

Reaction conditions: **1a** (0.6 mmol, 3.0 equiv.), **2a** (0.2 mmol, 1.0 equiv.), $AgNO_3$ (10 mol%), ligand (10 mol%), oxidant (0.6 mmol, 3.0 equiv.), DMF (2.0 mL), N_2, 24 h. [a] The yields were determined by GC using *n*-hexadecane as the internal standard.

3. General Procedures for Synthesis of Substrates

3.1 General Procedure for the Synthesis of Oxamic acids[1]

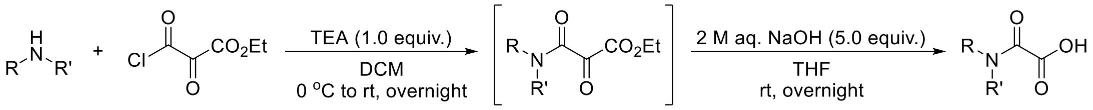

S1 **S2** **S3** **1**

The oxamic acids were prepared according to the reported literature.[1] Ethyl oxalyl chloride **S2** (0.62 mL, 5.5 mmol, 1.1 equiv.) was added dropwise to a solution of the amine **S1** (5 mmol, 1.0 equiv.) and triethylamine (0.8 mL, 5.5 mmol, 1.1 equiv.) at 0 °C under N_2 atmosphere. The solution was then allowed to warm to room temperature and stirred at room temperature for 3-6 h. After the reaction completed, 1 M HCl (aq.) (10 mL) was then added and the organic phase separated. The aqueous phase was then extracted with CH_2Cl_2 and the organic phases were combined, washed with brine, dried over Na_2SO_4 and the solvent was removed in vacuo. The residue of **S3** was then carried through to the next step without further purification.

The **S3** was then dissolved in THF (12.5 mL) followed by 2 M NaOH (aq.) (12.5 mL, 25 mmol, 2.5 equiv.) and the mixture stirred overnight at room temperature monitored via TLC. The mixture was then acidified (~ pH 1) with conc. HCl (aq.) followed by dilution with EtOAc. The layers were then separated, and the aqueous layer was extracted with more EtOAc. The organic phases were then combined, washed with brine, and dried over Na_2SO_4. The solvent was then removed in vacuo and the product **1** was either used without further purification.

4. General Procedures

4.1 General Procedures for

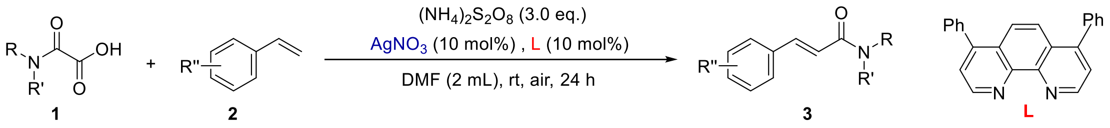

A 4 mL screw-cap vial equipped with a magnetic stirrer bar was charged with $AgNO_3$ (3.4 mg, 10 mol%), 4,7-diphenyl-1,10-phenanthroline (6.7 mg, 10 mol%), ammonium persulfate (136.9 mg, 0.6 mmol, 3.0 equiv.) and oxamic acid **1** (0.6 mmol, 3.0 equiv.). The vial was closed by PTFE/white rubber septum (Wheaton 13 mm Septa) and phenolic cap. Then aromatic vinyl (0.2 mmol, 1.0 equiv.) and DMF (2.0 mL) was injected. The mixture was stirred vigorously at room temperature for 24 h. After the reaction was complete, the solvent was removed in vacuo. The crude residue was purified by flash chromatography using petroleum ether/ethyl acetate or dichloromethane to afford the corresponding products.

4.2 Procedure for 2.0 mmol Scale Reaction

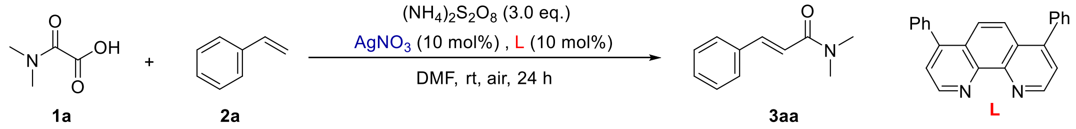

A 25 mL screw-cap vial equipped with a magnetic stirrer bar was charged with $AgNO_3$ (34 mg, 10 mol%), 4,7-diphenyl-1,10-phenanthroline (67 mg, 10 mol%), ammonium persulfate (1.37 g, 6.0 mmol, 3.0 equiv.) and oxamic acid **1a** (0.73 g, 6.0 mmol, 3.0 equiv.). The vial was closed by PTFE/white rubber septum and phenolic cap. Then styrene (0.23 mL, 2.0 mmol, 1.0 equiv.) and DMF (20 mL) was injected. The mixture was stirred vigorously at room temperature for 24 h. After the reaction was complete, the solvent was removed in vacuo. The crude residue was purified by flash chromatography using petroleum ether/ethyl acetate 1/1 to afford the pure products (white solid, 82% yield).

5. Characterization Data

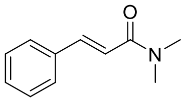

***N*,*N*-dimethylcinnamamide (3aa)**

30 mg, 85% yield, white solid. Eluent: petroleum ether/ethyl acetate = 1:1.

^{1}H NMR (400 MHz, $CDCl_3$) δ 7.67 (d, *J* = 15.4 Hz, 1H), 7.57 – 7.49 (m, 2H), 7.42 – 7.30 (m, 3H), 6.89 (d, *J* = 15.4 Hz, 1H), 3.13 (d, *J* = 40.8 Hz, 6H).

^{13}C NMR (101 MHz, $CDCl_3$) δ 166.7, 142.4, 135.4, 129.5, 128.8, 127.8, 117.4, 37.4, 36.0.

HRMS (ESI-TOF) m/z: $[M+H]^+$: Calcd. for $C_{11}H_{14}NO^+$ 176.1070, Found: 176.1080.

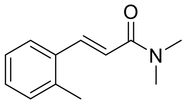

(E)-N,N-dimethyl-3-(o-tolyl)acrylamide (3ba)

28.7 mg, 76% yield, white solid. Eluent: petroleum ether/ethyl acetate = 1:1.

^{1}H NMR (400 MHz, $CDCl_3$) δ 7.94 (d, *J* = 15.3 Hz, 1H), 7.56 – 7.51 (m, 1H), 7.26 – 7.14 (m, 3H), 6.79 (d, *J* = 15.3 Hz, 1H), 3.13 (d, *J* = 38.6 Hz, 6H), 2.43 (s, 3H).

^{13}C NMR (101 MHz, $CDCl_3$) δ 166.8, 140.2, 137.5, 134.6, 130.7, 129.3, 126.2, 126.1, 118.8, 37.4, 35.9, 19.9.

HRMS (ESI-TOF) m/z: $[M+H]^+$: Calcd. for $C_{12}H_{16}NO^+$ 190.1226, Found: 190.1221.

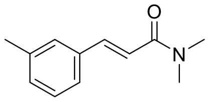

(E)-N,N-dimethyl-3-(m-tolyl)acrylamide (3ca)

23.8 mg, 63% yield, white solid. Eluent: petroleum ether/ethyl acetate = 1:1.

^{1}H NMR (400 MHz, $CDCl_3$) δ 7.64 (d, *J* = 15.4 Hz, 1H), 7.38 – 7.30 (m, 2H), 7.29 – 7.24 (m, 1H), 7.19 – 7.13 (m, 1H), 6.88 (d, *J* = 15.4 Hz, 1H), 3.13 (d, *J* = 43.1 Hz, 6H), 2.37 (s, 3H).

^{13}C NMR (101 MHz, $CDCl_3$) δ 166.8, 142.6, 138.4, 135.3, 130.4, 128.7, 128.4, 125.0, 117.2, 37.5, 36.0, 21.4.

HRMS (ESI-TOF) m/z: $[M+H]^+$: Calcd. for $C_{12}H_{16}NO^+$ 190.1226, Found: 190.1238.

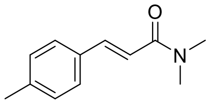

(*E*)-*N*,*N*-dimethyl-3-(*p*-tolyl)acrylamide (3ad)

26.8 mg, 71% yield, white solid. Eluent: petroleum ether/ethyl acetate = 1:1.

^{1}H NMR (400 MHz, $CDCl_3$) δ 7.65 (d, *J* = 15.4 Hz, 1H), 7.43 (d, *J* = 8.2 Hz, 2H), 7.18 (d, *J* = 7.9 Hz, 2H), 6.85 (d, *J* = 15.4 Hz, 1H), 3.12 (d, *J* = 41.1 Hz, 6H), 2.37 (s, 3H).

^{13}C NMR (101 MHz, $CDCl_3$) δ 166.9, 142.4, 139.8, 132.6, 129.5, 127.8, 116.3, 37.4, 36.0, 21.4.

HRMS (ESI-TOF) m/z: $[M+H]^+$: Calcd. for $C_{12}H_{16}NO^+$ 190.1226, Found: 190.1226.

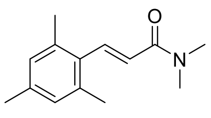

(*E*)-3-mesityl-*N*,*N*-dimethylacrylamide (3ae)

25.9 mg, 60% yield, white solid. Eluent: petroleum ether/ethyl acetate = 1:1.

^{1}H NMR (400 MHz, $CDCl_3$) δ 7.76 (d, *J* = 15.8 Hz, 1H), 6.89 (s, 2H), 6.48 (d, *J* = 15.8 Hz, 1H), 3.09 (d, *J* = 13.3 Hz, 6H), 2.32 (s, 6H), 2.28 (s, 3H).

^{13}C NMR (101 MHz, $CDCl_3$) δ 166.8, 140.8, 137.6, 136.4, 132.3, 129.0, 122.8, 37.4, 35.9, 21.1, 21.0.

HRMS (ESI-TOF) m/z: $[M+H]^+$: Calcd. for $C_{14}H_{20}NO^+$ 218.1539, Found: 218.1552.

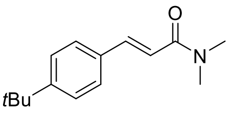

(*E*)-3-(4-(*tert*-butyl)phenyl)-*N*,*N*-dimethylacrylamide (3af)

37.8 mg, 82% yield, white solid. Eluent: petroleum ether/ethyl acetate = 1:1.

^{1}H NMR (400 MHz, $CDCl_3$) δ 7.66 (d, *J* = 15.4 Hz, 1H), 7.50 – 7.44 (m, 2H), 7.42 – 7.37 (m, 2H), 6.86 (d, *J* = 15.4 Hz, 1H), 3.12 (d, *J* = 40.8 Hz, 6H), 1.32 (s, 9H).

^{13}C NMR (101 MHz, $CDCl_3$) δ 166.9, 153.0, 142.3, 132.6, 127.6, 125.7, 116.5, 37.4, 36.0, 34.8, 31.2.

HRMS (ESI-TOF) m/z: $[M+H]^+$: Calcd. for $C_{15}H_{22}NO^+$ 232.1696, Found: 232.1723.

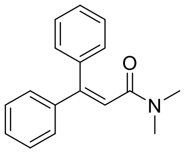

***N*,*N*-dimethyl-3,3-diphenylacrylamide (3ag)**

49.3 mg, 98% yield, white solid. Eluent: petroleum ether/ethyl acetate = 1:1.

^{1}H NMR (400 MHz, $CDCl_3$) δ 7.36 – 7.28 (m, 8H), 7.26 – 7.23 (m, 2H), 6.35 (s, 1H), 2.79 (d, *J* = 29.8 Hz, 6H).

^{13}C NMR (101 MHz, $CDCl_3$) δ 168.5, 147.4, 141.1, 139.0, 129.3, 128.5, 128.4, 128.3, 128.2, 128.2, 121.4, 37.7, 34.4.

HRMS (ESI-TOF) m/z: $[M+H]^+$: Calcd. for $C_{17}H_{18}NO^+$ 252.1383, Found: 252.1383.

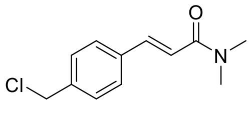

(*E*)-3-(4-(chloromethyl)phenyl)-*N*,*N*-dimethylacrylamide (3ah)

30.8 mg, 69% yield, white solid. Eluent: petroleum ether/ethyl acetate = 1:1.

^{1}H NMR (400 MHz, $CDCl_3$) δ 7.66 (d, *J* = 15.5 Hz, 1H), 7.55 – 7.50 (m, 2H), 7.42 – 7.37 (m, 2H), 6.90 (d, *J* = 15.4 Hz, 1H), 4.59 (s, 2H), 3.13 (d, *J* = 41.8 Hz, 6H).

^{13}C NMR (101 MHz, $CDCl_3$) δ 166.6, 141.6, 138.7, 135.5, 129.0, 128.1, 118.1, 45.8, 37.5, 36.0.

HRMS (ESI-TOF) m/z: $[M+H]^+$: Calcd. for $C_{12}H_{15}ClNO^+$ 224.0837, Found: 224.0835.

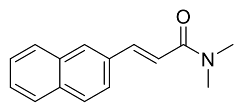

(*E*)-*N*,*N*-dimethyl-3-(naphthalen-2-yl)acrylamide (3ai)

33.3 mg, 74% yield, white solid. Eluent: petroleum ether/ethyl acetate = 1:1.

^{1}H NMR (400 MHz, $CDCl_3$) δ 7.93 (s, 1H), 7.90 – 7.78 (m, 4H), 7.72 – 7.65 (m, 1H), 7.54 – 7.45 (m, 2H), 7.01 (d, *J* = 15.4 Hz, 1H), 3.16 (d, *J* = 48.2 Hz, 6H).

^{13}C NMR (101 MHz, $CDCl_3$) δ 166.8, 142.5, 133.9, 133.4, 132.8, 129.2, 128.5, 128.4, 127.7, 126.9, 126.6, 123.7, 117.5, 37.5, 36.0.

HRMS (ESI-TOF) m/z: $[M+H]^+$: Calcd. for $C_{15}H_{16}NO^+$ 226.1226, Found: 226.1225.

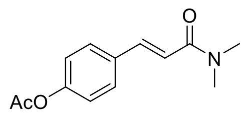

(*E*)-4-(3-(dimethylamino)-3-oxoprop-1-en-1-yl)phenyl acetate (3aj)

34.9 mg, 75% yield, white solid. Eluent: petroleum ether/ethyl acetate = 1:1.

^{1}H NMR (400 MHz, $CDCl_3$) δ 7.65 (d, *J* = 15.4 Hz, 1H), 7.57 – 7.51 (m, 2H), 7.13 – 7.06 (m, 2H), 6.84 (d, *J* = 15.4 Hz, 1H), 3.12 (d, *J* = 40.0 Hz, 6H), 2.31 (s, 3H).

^{13}C NMR (101 MHz, $CDCl_3$) δ 169.3, 166.6, 151.5, 141.3, 133.2, 128.9, 122.0, 117.6, 37.4, 36.0, 21.2.

HRMS (ESI-TOF) m/z: $[M+H]^+$: Calcd. for $C_{13}H_{16}NO_3^+$ 234.1125, Found: 234.1118.

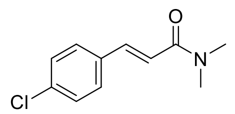

(*E*)-3-(4-chlorophenyl)-*N*,*N*-dimethylacrylamide (3ak)

32.7 mg, 78% yield, white solid. Eluent: petroleum ether/ethyl acetate = 1:1.

^{1}H NMR (400 MHz, $CDCl_3$) δ 7.62 (d, *J* = 15.4 Hz, 1H), 7.48 – 7.43 (m, 2H), 7.37 – 7.32 (m, 2H), 6.86 (d, *J* = 15.4 Hz, 1H), 3.12 (d, *J* = 41.1 Hz, 6H).

^{13}C NMR (101 MHz, $CDCl_3$) δ 166.4, 141.0, 135.3, 133.9, 129.0, 129.0, 118.0, 37.4, 36.0.

HRMS (ESI-TOF) m/z: $[M+H]^+$: Calcd. for $C_{11}H_{13}ClNO^+$ 210.0680, Found: 210.0676.

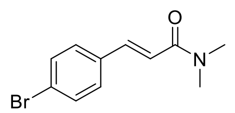

(*E*)-3-(4-bromophenyl)-*N*,*N*-dimethylacrylamide (3al)

43.7 mg, 86% yield, white solid. Eluent: petroleum ether/ethyl acetate = 1:1.

^{1}H NMR (400 MHz, $CDCl_3$) δ 7.60 (d, *J* = 15.4 Hz, 1H), 7.54 – 7.46 (m, 2H), 7.42 – 7.35 (m, 2H), 6.88 (d, *J* = 15.4 Hz, 1H), 3.12 (d, *J* = 41.2 Hz, 6H).

^{13}C NMR (101 MHz, $CDCl_3$) δ 166.4, 141.1, 134.3, 132.0, 129.2, 123.6, 118.1, 37.4, 36.0.

HRMS (ESI-TOF) m/z: $[M+H]^+$: Calcd. for $C_{11}H_{12}BrNO^+$ 254.0175, Found: 254.0181.

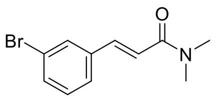

(*E*)-3-(3-bromophenyl)-*N*,*N*-dimethylacrylamide (3am)

% yield, white solid. Eluent: dichloromethane.

^{1}H NMR (400 MHz, $CDCl_3$) δ 7.68 (t, *J* = 1.9 Hz, 1H), 7.59 (d, *J* = 15.4 Hz, 1H), 7.49 – 7.40 (m, 2H), 7.25 – 7.21 (m, 1H), 6.88 (d, *J* = 15.4 Hz, 1H), 3.13 (d, *J* = 42.8 Hz, 6H).

^{13}C NMR (101 MHz, $CDCl_3$) δ 166.2, 140.7, 137.5, 132.3, 130.3, 130.1, 126.8, 122.9, 118.9, 37.5, 36.0.

HRMS (ESI-TOF) m/z: $[M+H]^+$: Calcd. for $C_{11}H_{12}BrNO^+$ 254.0175, Found:254.0165.

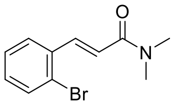

(*E*)-3-(2-bromophenyl)-*N*,*N*-dimethylacrylamide (3an)

% yield, colorless oil. Eluent: dichloromethane.

^{1}H NMR (400 MHz, $CDCl_3$) δ 7.95 (d, *J* = 15.4 Hz, 1H), 7.62 – 7.55 (m, 2H), 7.33 – 7.28 (m, 1H), 7.22 – 7.17 (m, 1H), 6.82 (d, *J* = 15.4 Hz, 1H), 3.13 (d, *J* = 37.4 Hz, 6H).

^{13}C NMR (101 MHz, $CDCl_3$) δ 166.3, 140.8, 135.7, 133.4, 130.4, 127.8, 127.5, 125.0, 120.9, 37.5, 35.9.

HRMS (ESI-TOF) m/z: $[M+H]^+$: Calcd. for $C_{11}H_{13}BrNO^+$ 254.0175, Found: 254.0170.

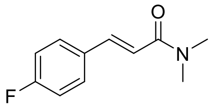

(*E*)-3-(4-fluorophenyl)-*N*,*N*-dimethylacrylamide (3ao)

25.1 mg, 65% yield, white solid. Eluent: petroleum ether/ethyl acetate = 1:1.

^{1}H NMR (400 MHz, $CDCl_3$) δ 7.63 (d, *J* = 15.4 Hz, 1H), 7.56 – 7.46 (m, 2H), 7.06 (t, *J* = 8.5 Hz, 2H), 6.81 (d, *J* = 15.4 Hz, 1H), 3.12 (d, *J* = 41.1 Hz, 6H).

^{13}C NMR (101 MHz, $CDCl_3$) δ 166.6, 164.7, 162.2, 141.2, 131.6, 131.6, 129.6, 129.5, 117.2, 117.1, 116.0, 115.8, 37.4, 36.0.

HRMS (ESI-TOF) m/z: $[M+H]^+$: Calcd. for $C_{11}H_{12}FNO^+$ 194.0976, Found: 194.0982.

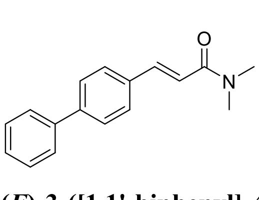

(*E*)-3-([1,1'-biphenyl]-4-yl)-*N*,*N*-dimethylacrylamide (3ap)

35.3 mg, 72% yield, white solid. Eluent: petroleum ether/ethyl acetate = 1:1.

^{1}H NMR (400 MHz, $CDCl_3$) δ 7.71 (d, *J* = 15.4 Hz, 1H), 7.63 – 7.59 (m, 6H), 7.48 – 7.43 (m, 2H), 7.39 – 7.34 (m, 1H), 6.93 (d, *J* = 15.4 Hz, 1H), 3.14 (d, *J* = 44.3 Hz, 6H).

^{13}C NMR (101 MHz, $CDCl_3$) δ 166.7, 142.3, 141.9, 140.3, 134.4, 128.9, 128.3, 127.7, 127.5, 127.0, 117.3, 37.5, 36.0.

HRMS (ESI-TOF) m/z: $[M+H]^+$: Calcd. for $C_{17}H_{18}NO^+$ 252.1383, Found: 252.1382.

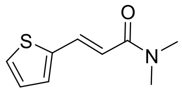

(*E*)-*N*,*N*-dimethyl-3-(thiophen-2-yl)acrylamide (3aq)

31.9 mg, 88% yield, white solid. Eluent: petroleum ether/ethyl acetate = 1:1.

^{1}H NMR (400 MHz, $CDCl_3$) δ 7.79 (d, *J* = 15.1 Hz, 1H), 7.31 (d, *J* = 5.1 Hz, 1H), 7.21 (d, *J* = 3.6 Hz, 1H), 7.05 – 7.02 (m, 1H), 6.69 (d, *J* = 15.1 Hz, 1H), 3.11 (d, *J* = 38.2 Hz, 6H).

^{13}C NMR (101 MHz, $CDCl_3$) δ 166.4, 140.6, 135.1, 130.2, 128.0, 127.1, 116.2, 37.4, 36.0.

HRMS (ESI-TOF) m/z: [M+H]$^{+}$: Calcd. for $C_9H_{11}NOS^+$ 182.0634, Found: 182.0632.

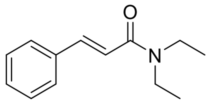

***N*,*N*-diethylcinnamamide (3ba)**

31.2 mg, 77% yield, white solid. Eluent: petroleum ether/ethyl acetate = 1:1.

^{1}H NMR (400 MHz, $CDCl_3$) δ 7.71 (d, *J* = 15.4 Hz, 1H), 7.55 – 7.50 (m, 2H), 7.41 – 7.31 (m, 3H), 6.83 (d, *J* = 15.4 Hz, 1H), 3.54 – 3.45 (m, 4H), 1.27 (t, *J* = 7.0 Hz, 3H), 1.19 (t, *J* = 7.1 Hz, 3H).

^{13}C NMR (101 MHz, $CDCl_3$) δ 165.7, 142.3, 135.5, 129.4, 128.8, 127.8, 117.8, 42.3, 41.1, 15.1, 13.2.

HRMS (ESI-TOF) m/z: [M+H]$^{+}$: Calcd. for $C_{13}H_{18}NO^+$ 204.1383, Found: 204.1382.

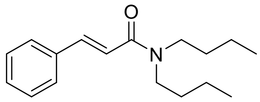

***N*,*N*-dibutylcinnamamide (3ca)**

41.4 mg, 80% yield, colorless oil. Eluent: dichloromethane.

^{1}H NMR (400 MHz, $CDCl_3$) δ 7.70 (d, *J* = 15.4 Hz, 1H), 7.54 – 7.48 (m, 2H), 7.41 – 7.31 (m, 3H), 6.83 (d, *J* = 15.4 Hz, 1H), 3.47 – 3.35 (m, 4H), 1.63 – 1.54 (m, 4H), 1.43 – 1.30 (m, 4H), 1.01 – 0.91 (m, 6H).

^{13}C NMR (101 MHz, $CDCl_3$) δ 166.0, 142.2, 135.6, 129.4, 128.8, 127.7, 117.9, 48.0, 46.7, 32.0, 30.1, 20.3, 20.1, 13.9, 13.9.

HRMS (ESI-TOF) m/z: [M+H]$^{+}$: Calcd. for $C_{17}H_{26}NO^+$ 260.2009, Found: 260.2008.

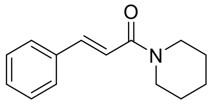

(*E*)-3-phenyl-1-(piperidin-1-yl)prop-2-en-1-one (3da)

30.1 mg, 71% yield, white solid. Eluent: petroleum ether /ethyl acetate = 1:1.

^{1}H NMR (400 MHz, $CDCl_3$) δ 7.64 (d, *J* = 15.5 Hz, 1H), 7.55 – 7.49 (m, 2H), 7.40 – 7.31 (m, 3H), 6.90 (d, *J* = 15.5 Hz, 1H), 3.63 (d, *J* = 31.6 Hz, 4H), 1.70 – 1.66 (m, 2H), 1.65 – 1.58 (m, 4H).

^{13}C NMR (101 MHz, $CDCl_3$) δ 165.4, 142.2, 135.5, 129.4, 128.8, 127.7, 117.8, 47.0, 43.4, 26.8, 25.6, 24.7.

HRMS (ESI-TOF) m/z: [M+H]$^{+}$: Calcd. for $C_{14}H_{18}NO^+$ 216.1383, Found: 216.1392.

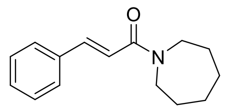

(*E*)-1-(azepan-1-yl)-3-phenylprop-2-en-1-one (3ea)

31.1 mg, 68% yield, white solid. Eluent: petroleum ether /ethyl acetate = 1:1.

^{1}H NMR (400 MHz, CDCl$_3$) δ 7.70 (d, J = 15.4 Hz, 1H), 7.56 – 7.50 (m, 2H), 7.40 – 7.33 (m, 3H), 6.87 (d, J = 15.4 Hz, 1H), 3.68 – 3.58 (m, 4H), 1.82 – 1.75 (m, 4H), 1.64 – 1.55 (m, 4H).

^{13}C NMR (101 MHz, CDCl$_3$) δ 166.3, 142.3, 135.5, 129.4, 128.8, 127.8, 117.8, 48.0, 46.6, 29.4, 27.7, 27.0, 26.6.

HRMS (ESI-TOF) m/z: [M+H]$^+$: Calcd. for $C_{15}H_{20}NO^+$ 230.1539, Found: 230.1555.

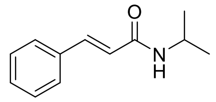

***N*-isopropylcinnamamide (3ga)**

23.8, 63% yield, white solid. Eluent: dichloromethane.

^{1}H NMR (400 MHz, CDCl$_3$) δ 7.61 (d, J = 15.6 Hz, 1H), 7.51 – 7.46 (m, 2H), 7.37 – 7.33 (m, 3H), 6.37 (d, J = 15.6 Hz, 1H), 5.56 (d, J = 5.5 Hz, 1H), 4.28 – 4.18 (m, 1H), 1.22 (d, J = 6.6 Hz, 6H).

^{13}C NMR (101 MHz, CDCl$_3$) δ 165.0, 140.7, 134.9, 129.6, 128.8, 127.7, 121.1, 41.6, 22.9.

HRMS (ESI-TOF) m/z: [M+H]$^+$: Calcd. for $C_{12}H_{16}NO^+$ 190.1226, Found: 190.1225.

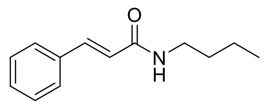

***N*-butylcinnamamide (3ha)**

31.3 mg, 77% yield, white solid. Eluent: dichloromethane.

^{1}H NMR (400 MHz, CDCl$_3$) δ 7.62 (d, J = 15.6 Hz, 1H), 7.52 – 7.47 (m, 2H), 7.39 – 7.32 (m, 3H), 6.39 (d, J = 15.6 Hz, 1H), 5.70 (s, 1H), 3.43 – 3.36 (m, 2H), 1.56 (p, J = 7.3 Hz, 2H), 1.45 – 1.35 (m, 2H), 0.95 (t, J = 7.3 Hz, 3H).

^{13}C NMR (101 MHz, CDCl$_3$) δ 165.9, 140.8, 134.9, 129.6, 128.8, 127.8, 120.8, 39.5, 31.8, 20.1, 13.8.

HRMS (ESI-TOF) m/z: [M+H]$^+$: Calcd. for $C_{13}H_{18}NO^+$ 204.1383, Found: 204.1377.

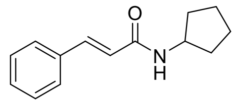

***N*-cyclopentylcinnamamide (3ia)**

24.5 mg, 57% yield, white solid. Eluent: dichloromethane.

^{1}H NMR (400 MHz, CDCl$_3$) δ 7.61 (d, J = 15.6 Hz, 1H), 7.52 – 7.46 (m, 2H), 7.40 – 7.31 (m, 3H), 6.37 (d, J = 15.6 Hz, 1H), 5.63 (d, J = 5.3 Hz, 1H), 4.40 – 4.30 (m, 1H), 2.11 – 2.01 (m, 2H), 1.71 – 1.63 (m, 4H), 1.50 – 1.40 (m, 2H).

^{13}C NMR (101 MHz, CDCl$_3$) δ 165.5, 140.7, 135.0, 129.6, 128.8, 127.7, 120.9, 51.4, 33.3, 23.8.

HRMS (ESI-TOF) m/z: [M+H]$^+$: Calcd. for $C_{14}H_{18}NO^+$ 216.1838, Found:216.1838.

6. Mechanism study experiment

6.1 Radical inhibition experiment

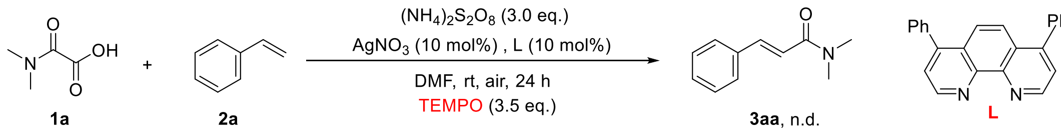

A 4 mL screw-cap vial equipped with a magnetic stirrer bar was charged with $AgNO_3$ (3.4 mg, 10 mol%), 4,7-diphenyl-1,10-phenanthroline (6.7 mg, 10 mol%), ammonium persulfate (136.9 mg, 0.6 mmol, 3.0 equiv.), oxamic acid **1a** (70.2 mg, 0.6 mmol, 3.0 equiv.) and TEMPO (109.4 mg, 0.7 mmol, 3.5 equiv.). The vial was closed by PTFE/white rubber septum (Wheaton 13 mm Septa) and phenolic cap. Then styrene (23 µL, 0.2 mmol, 1.0 equiv.) and DMF (2.0 mL) was injected. The mixture was stirred vigorously at room temperature for 24 h. Monitored by GCMS.

6.2 Radical capture experiment

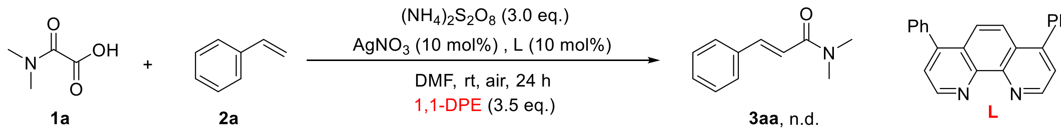

A 4 mL screw-cap vial equipped with a magnetic stirrer bar was charged with $AgNO_3$ (3.4 mg, 10 mol%), 4,7-diphenyl-1,10-phenanthroline (6.7 mg, 10 mol%), ammonium persulfate (136.9 mg, 0.6 mmol, 3.0 equiv.), oxamic acid **1a** (70.2 mg, 0.6 mmol, 3.0 equiv.) and 1,1-DPE (126.2 mg, 0.7 mmol, 3.5 equiv.). The vial was closed by PTFE/white rubber septum (Wheaton 13 mm Septa) and phenolic cap. Then styrene (23 µL, 0.2 mmol, 1.0 equiv.) and DMF (2.0 mL) was injected. The mixture was stirred vigorously at room temperature for 24 h. After the reaction was complete, the solvent was removed in vacuo. The crude residue was purified by flash chromatography using petroleum ether/ethyl acetate 1/1 to afford the main product **3ag**.

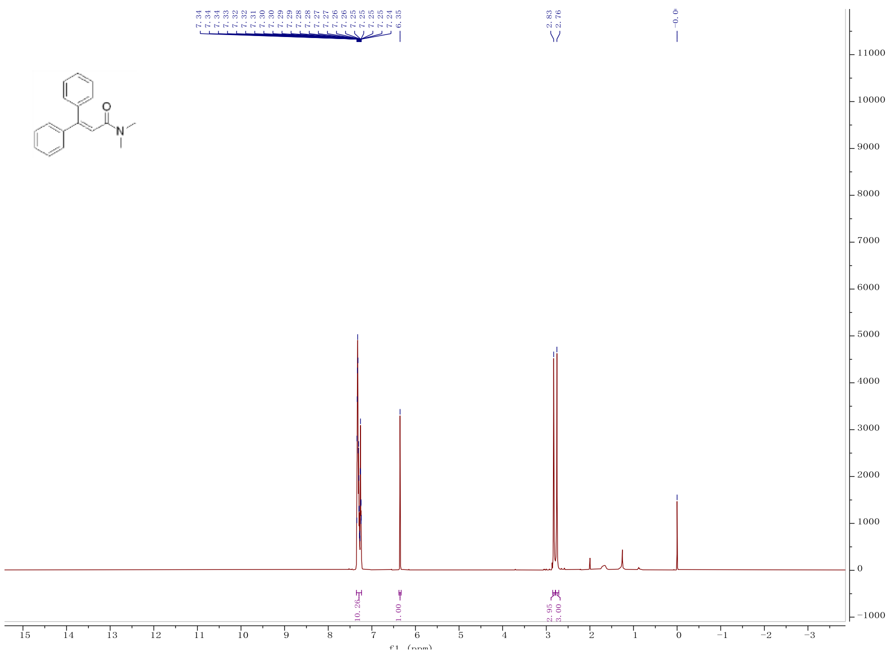

6.3 Procedure for the Synthesis of Intermediates D

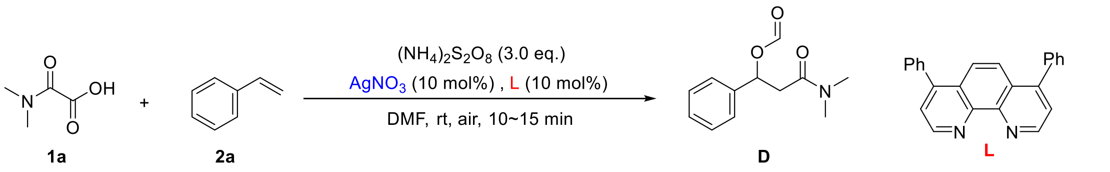

A 4 mL screw-cap vial equipped with a magnetic stirrer bar was charged with $AgNO_3$ (3.4 mg, 10 mol%), 4,7-diphenyl-1,10-phenanthroline (6.7 mg, 10 mol%), ammonium persulfate (136.9 mg, 0.6 mmol, 3.0 equiv.) and oxamic acid **1a** (70.2 mg, 0.6 mmol, 3.0 equiv.). The vial was closed by PTFE/white rubber septum (Wheaton 13 mm Septa) and phenolic cap. Then styrene (23 µL, 0.2 mmol, 1.0 equiv.) and DMF (2.0 mL) was injected. The mixture was stirred vigorously at room temperature for 10~15 min. After the reaction was complete, the mixture was poured into 15 mL of water and extracted with ethyl acetate three times. The organic phases were combined, and the combined organic phase was extracted once with water, followed by a back-extraction of the aqueous phase with ethyl acetate. Finally, the combined organic phase was washed with brine, dried over Na_2SO_4, and the solvent was removed in vacuo The crude residue was purified by flash chromatography using petroleum ether/ethyl acetate (1:1-1:2) to afford the intermediate **D** (colorless oil, 53% yield). **^{1}H NMR (400 MHz, $CDCl_3$)** δ 8.09 (s, 1H), 7.44 – 7.29 (m, 5H), 6.34 (dd, J = 8.7, 4.5 Hz, 1H), 3.07 (dd, J = 15.9, 8.8 Hz, 1H), 2.96 (d, J = 12.2 Hz, 6H), 2.73 (dd, J = 15.9, 4.5 Hz, 1H). **^{13}C NMR (101 MHz, $CDCl_3$)** δ 168.8, 160.2, 139.5, 128.7, 128.4, 126.5, 73.0, 40.1, 37.2, 35.5. **HRMS** (ESI-TOF) m/z: $[M+H]^+$: calcd. for $C_{12}H_{16}NO^+$ 222.1125, found: 222.1117. The intermediate is unstable and will transform into the final product **3aa** easily through decarboxylation and dehydration.

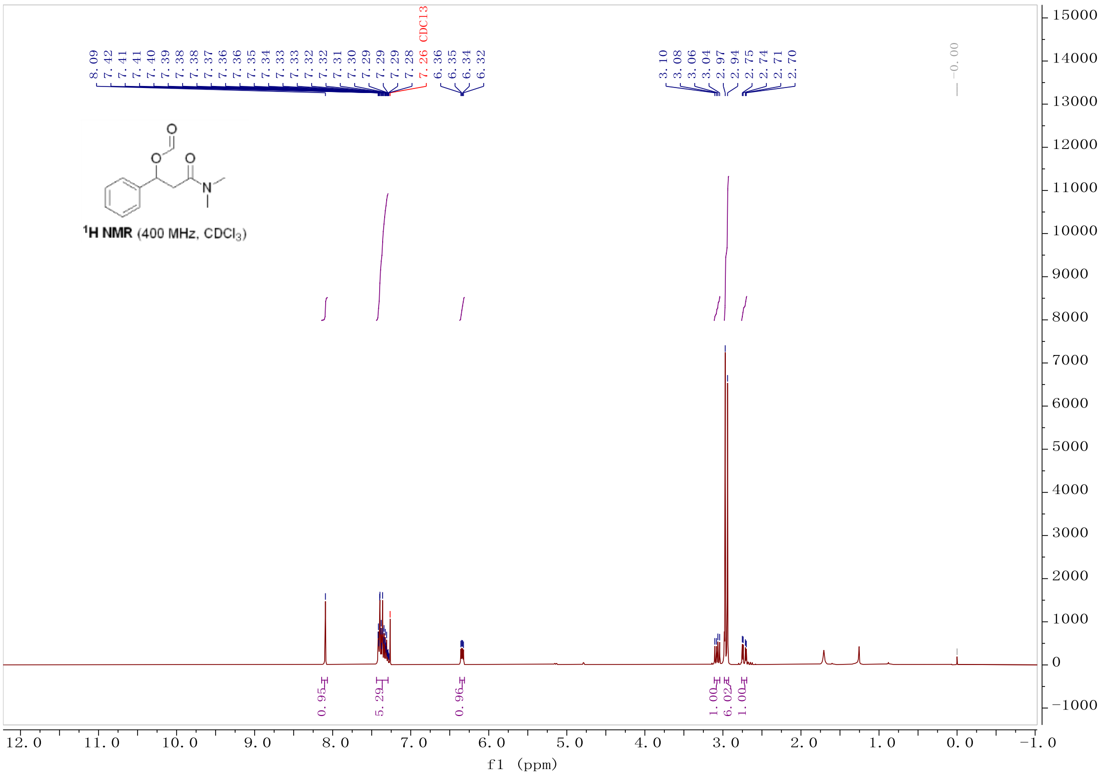

8.09
7.42
7.41
7.41
7.40
7.39
7.38
7.38
7.37
7.36
7.36
7.35
7.34
7.33
7.33
7.32
7.32
7.31
7.30
7.29
7.29
7.29
7.28
7.26 CDCl3
6.36
6.35
6.34
6.32
3.10
3.08
3.06
3.04
2.97
2.94
2.75
2.74
2.71
2.70
-0.00
O
O
O
N
1H NMR (400 MHz, CDCl3)
0.95
5.29
0.96
1.00
6.02
1.00
f1 (ppm)

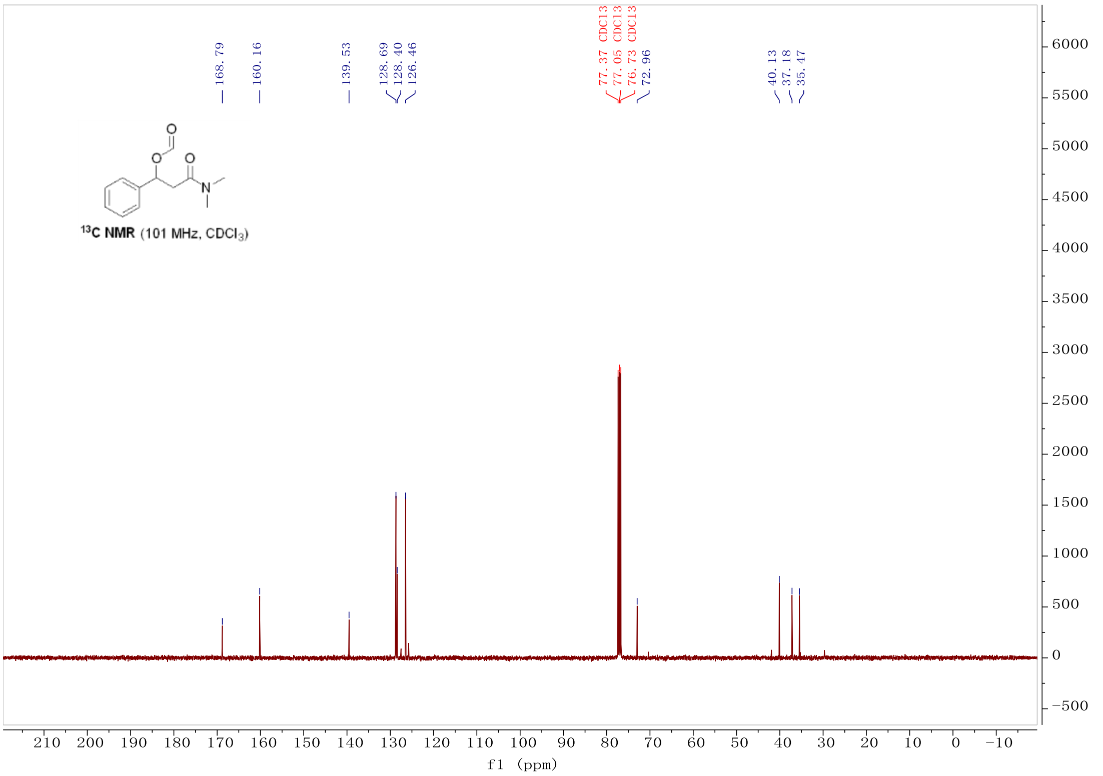

168.79
160.16
139.53
128.69
128.40
126.46
77.37 CDCl3
77.05 CDCl3
76.73 CDCl3
72.96
40.13
37.18
35.47
O
O
O
N
13C NMR (101 MHz, CDCl3)
f1 (ppm)

7. References

1. Kitcatt, D. M.; Scott, K. A.; Rongione, E.; Nicolle, S.; Lee, A.-L. Direct Decarboxylative Giese Amidations: Photocatalytic vs. Metal- and Light-Free. *Chem. Sci.* **2023***, 14,* 9806-9813.

8. Copies of NMR Spectra

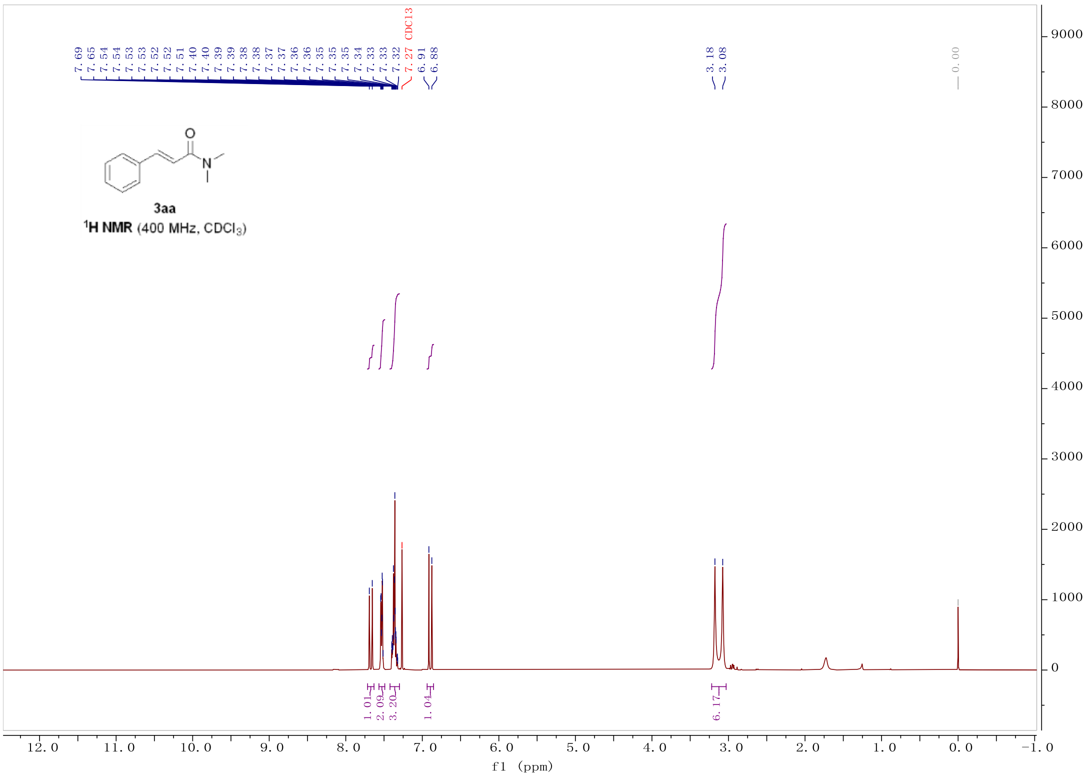

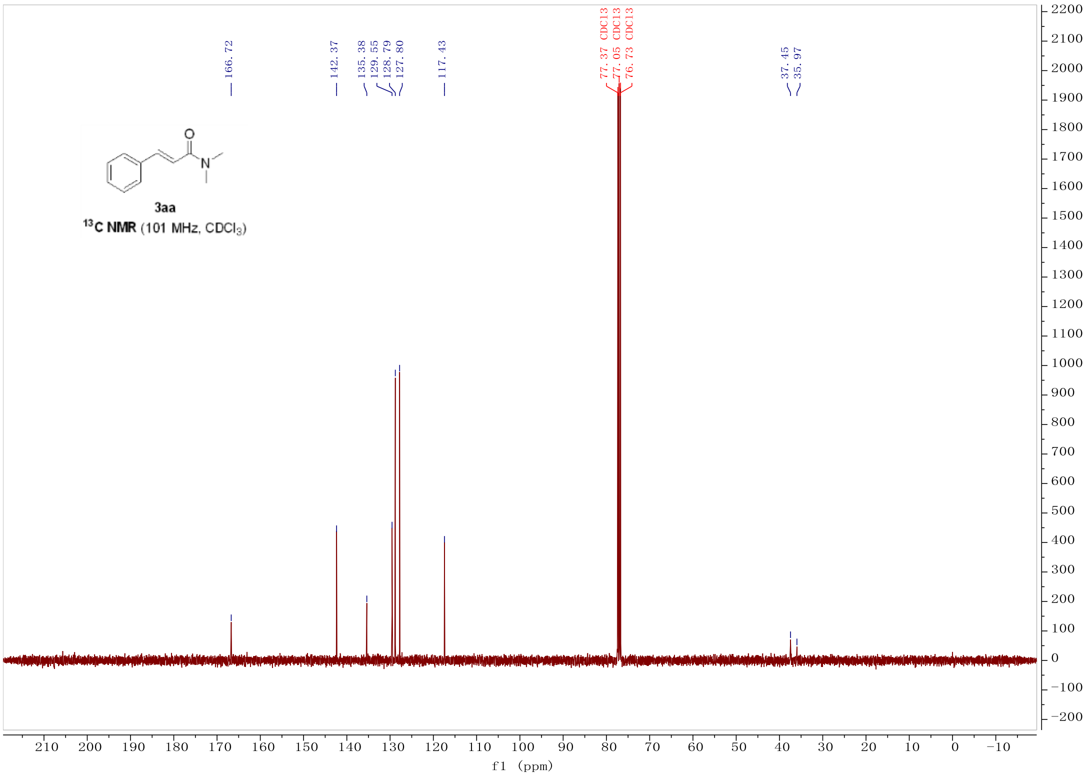

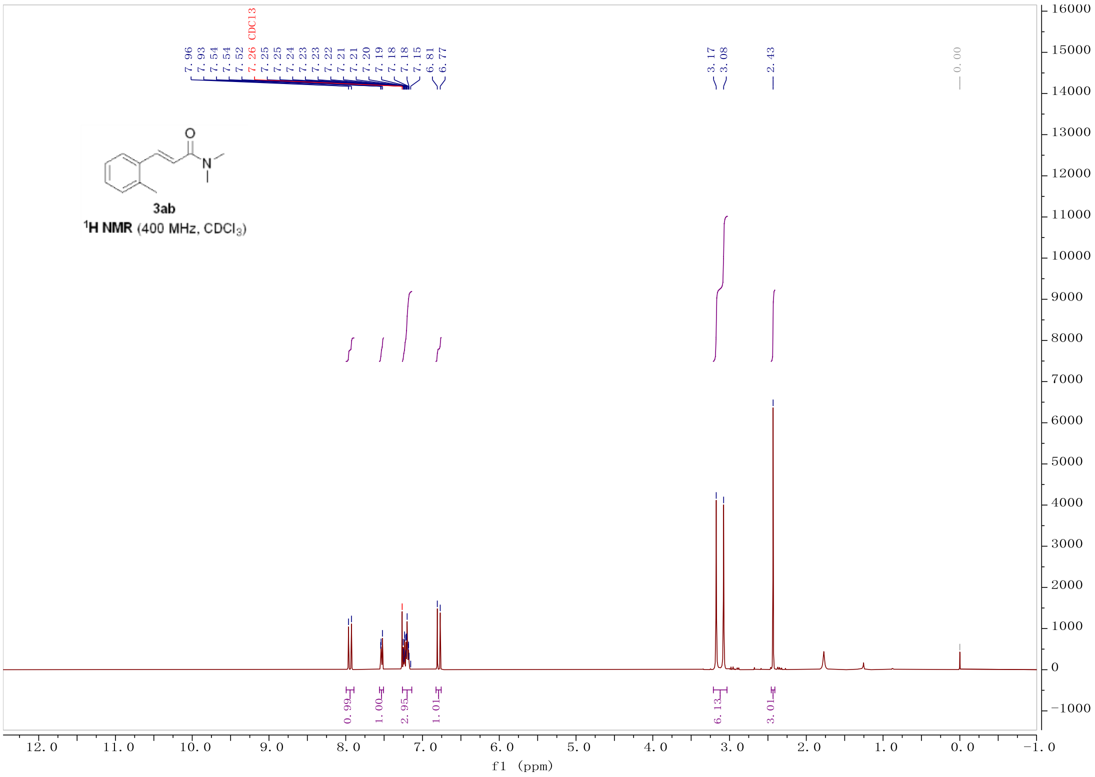
7.96 7.93 7.54 7.54 7.52 7.26 CDCl3 7.25 7.25 7.24 7.23 7.23 7.22 7.21 7.21 7.20 7.19 7.18 7.18 7.15 6.81 6.77
3.17 3.08
2.43
0.00
3ab
1H NMR (400 MHz, CDCl3)
0.99 1.00 2.95 1.01 6.13 3.01
f1 (ppm)

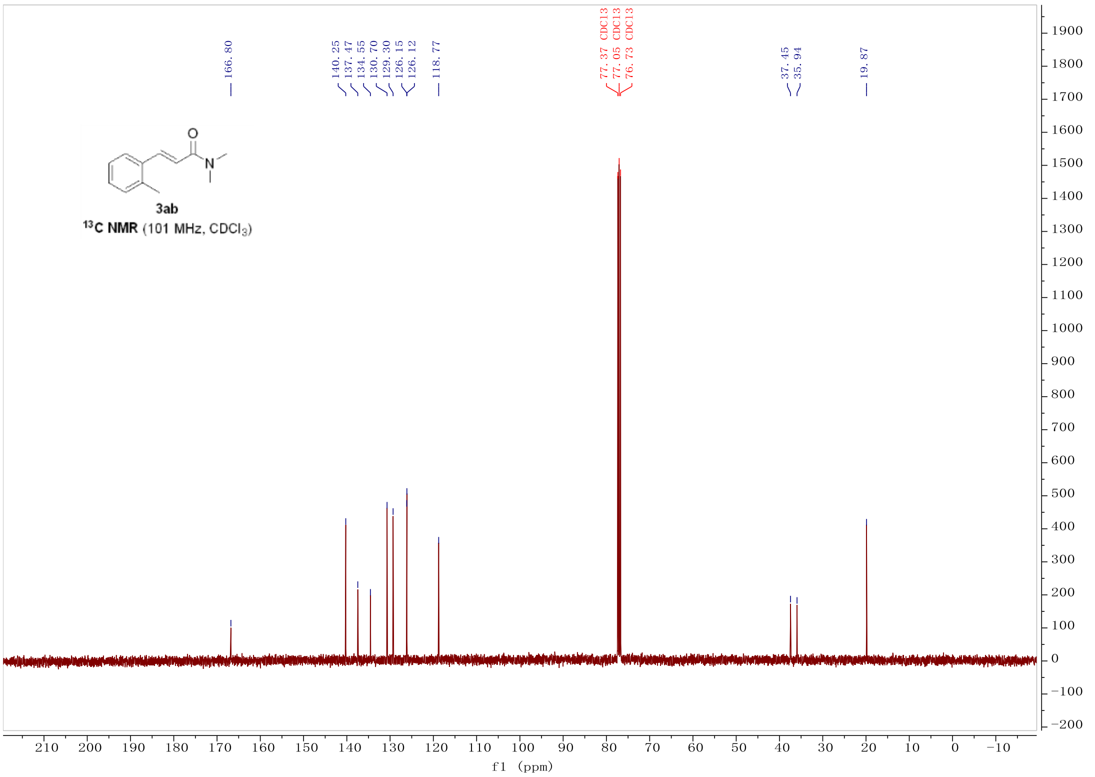
166.80
140.25 137.47 134.55 130.70 129.30 126.15 126.12 118.77
77.37 CDCl3 77.05 CDCl3 76.73 CDCl3
37.45 35.94
19.87
3ab
13C NMR (101 MHz, CDCl3)
f1 (ppm)

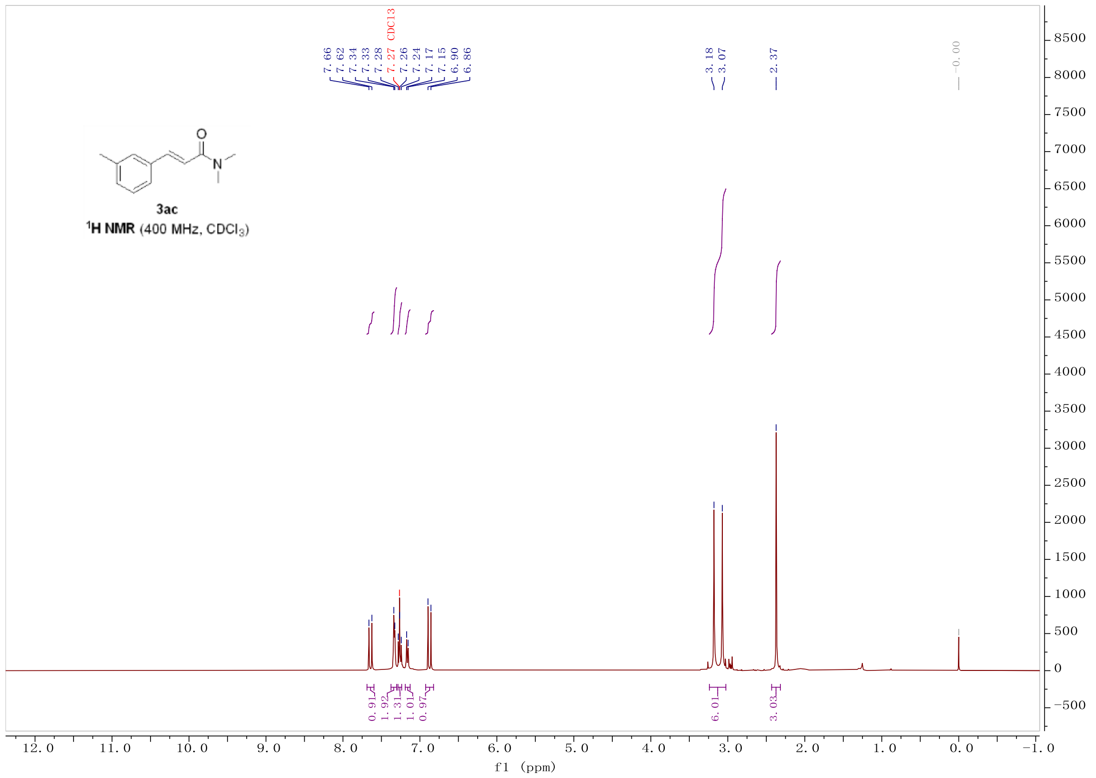

3ac
1H NMR (400 MHz, CDCl3)
7.66 7.62 7.34 7.33 7.28 7.27 CDCl3 7.26 7.24 7.17 7.15 6.90 6.86
3.18 3.07
2.37
0.00
0.91 1.92 1.31 1.01 0.97
6.01
3.03
f1 (ppm)

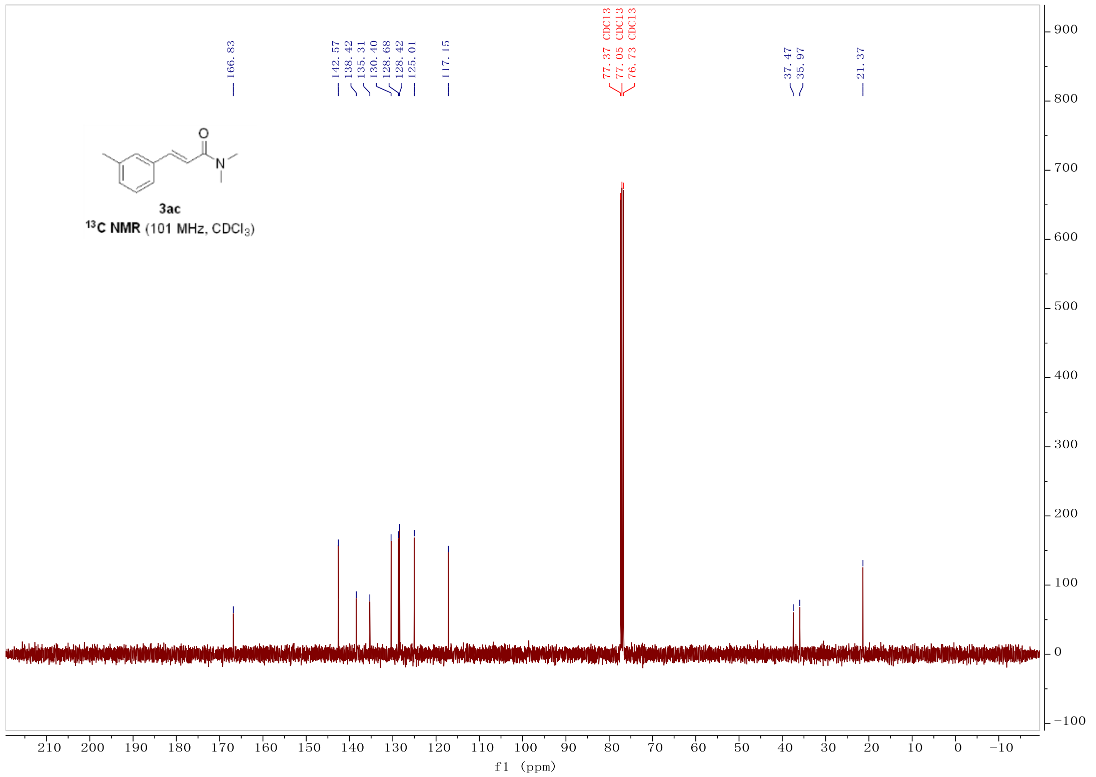

3ac
13C NMR (101 MHz, CDCl3)
166.83
142.57 138.42 135.31 130.40 128.68 128.42 125.01
117.15
77.37 CDCl3 77.05 CDCl3 76.73 CDCl3
37.47 35.97
21.37
f1 (ppm)

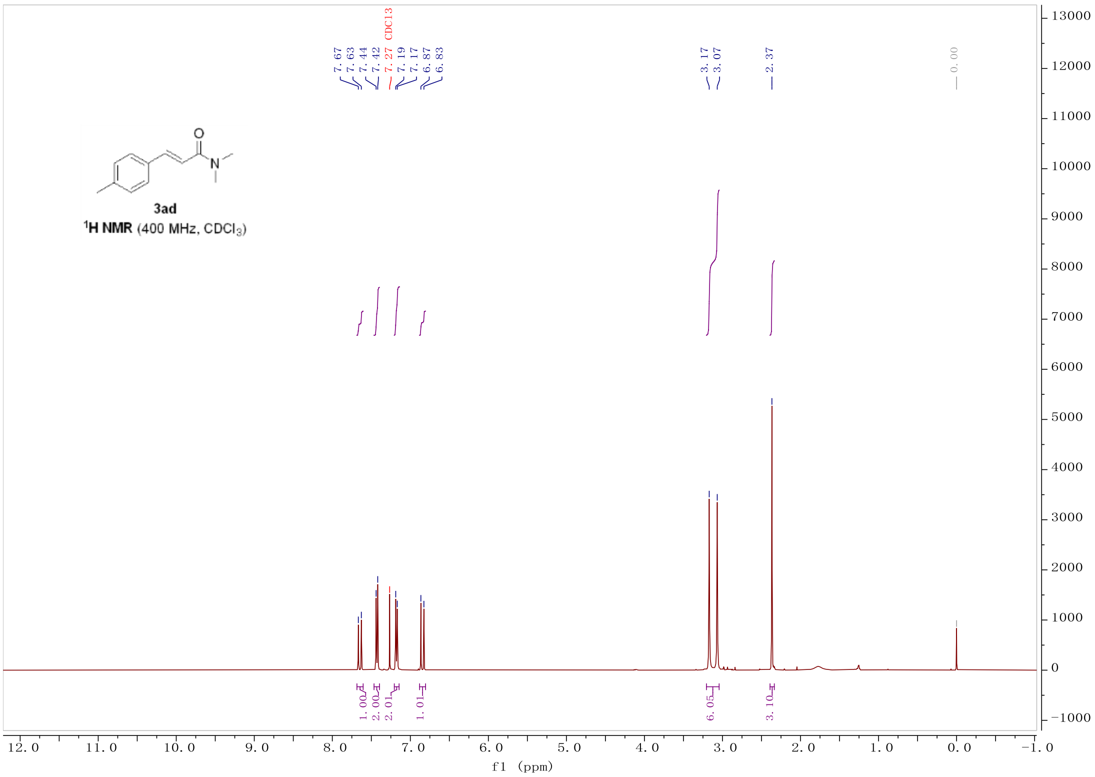

7.67
7.63
7.44
7.42
7.27 CDCl3
7.19
7.17
6.87
6.83
3.17
3.07
2.37
0.00
3ad
^{1}H NMR (400 MHz, $CDCl_3$)
1.00
2.00
2.01
1.01
6.05
3.10
f1 (ppm)

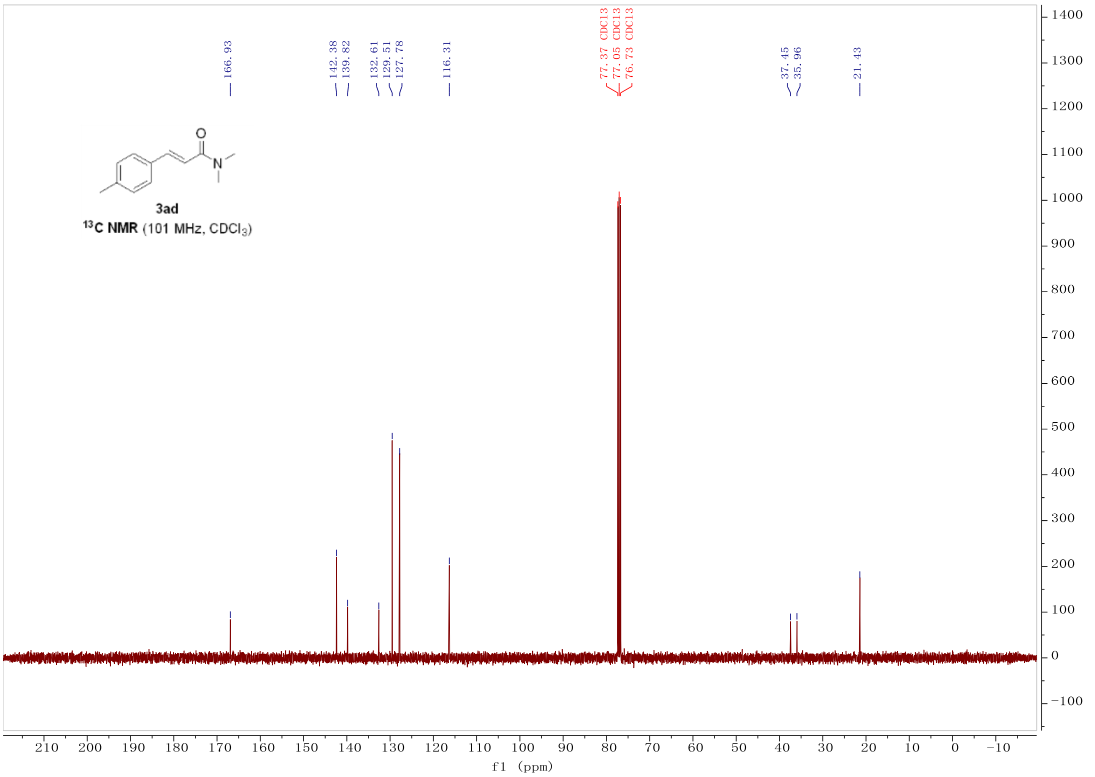

166.93
142.38
139.82
132.61
129.51
127.78
116.31
77.37 CDCl3
77.05 CDCl3
76.73 CDCl3
37.45
35.96
21.43
3ad
^{13}C NMR (101 MHz, $CDCl_3$)
f1 (ppm)

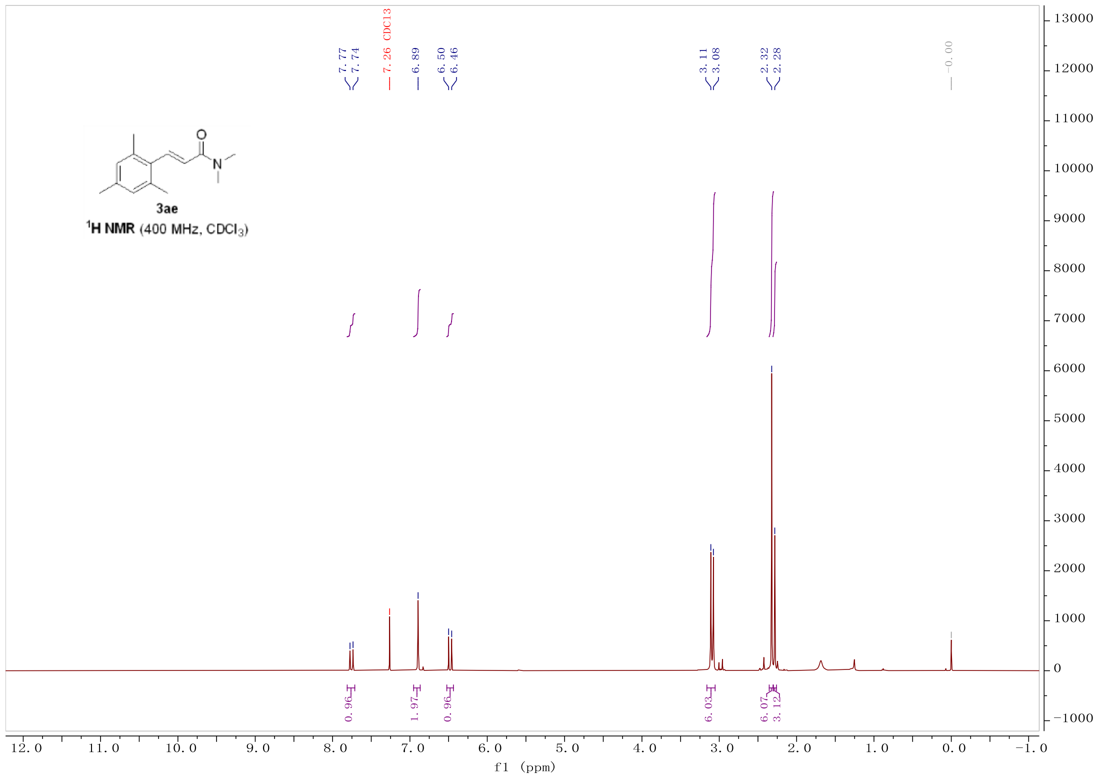
3ae
¹H NMR (400 MHz, CDCl3)
f1 (ppm)

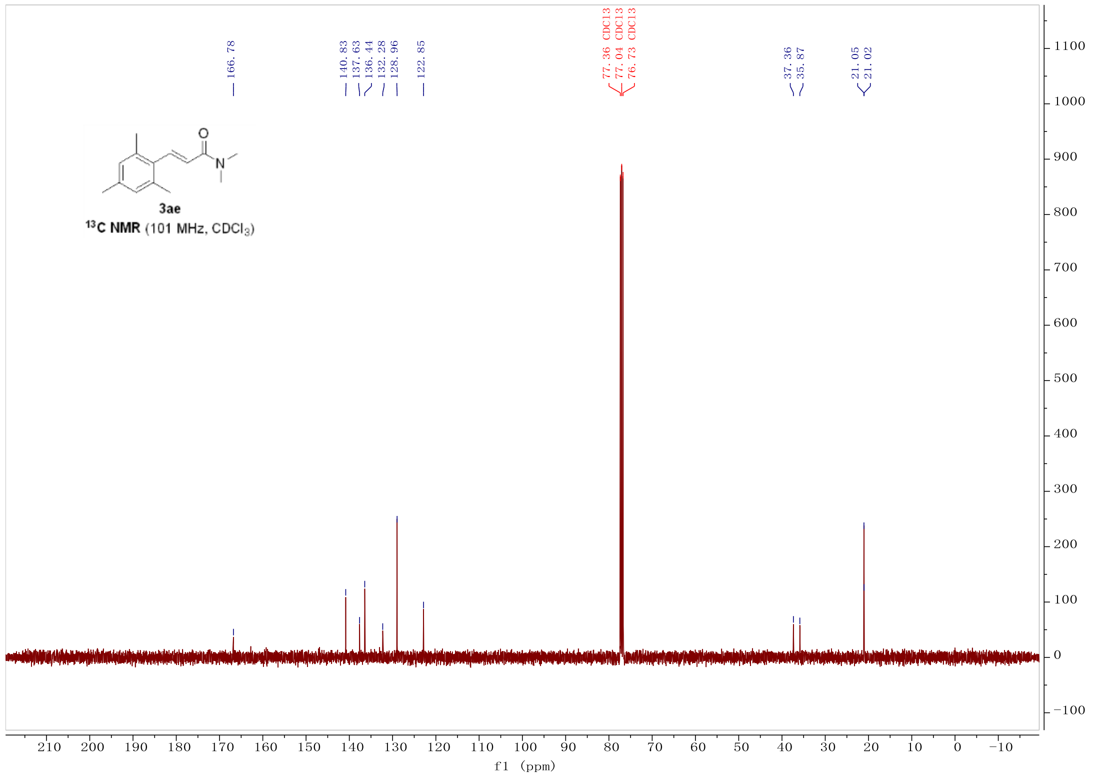
3ae
¹³C NMR (101 MHz, CDCl3)
f1 (ppm)

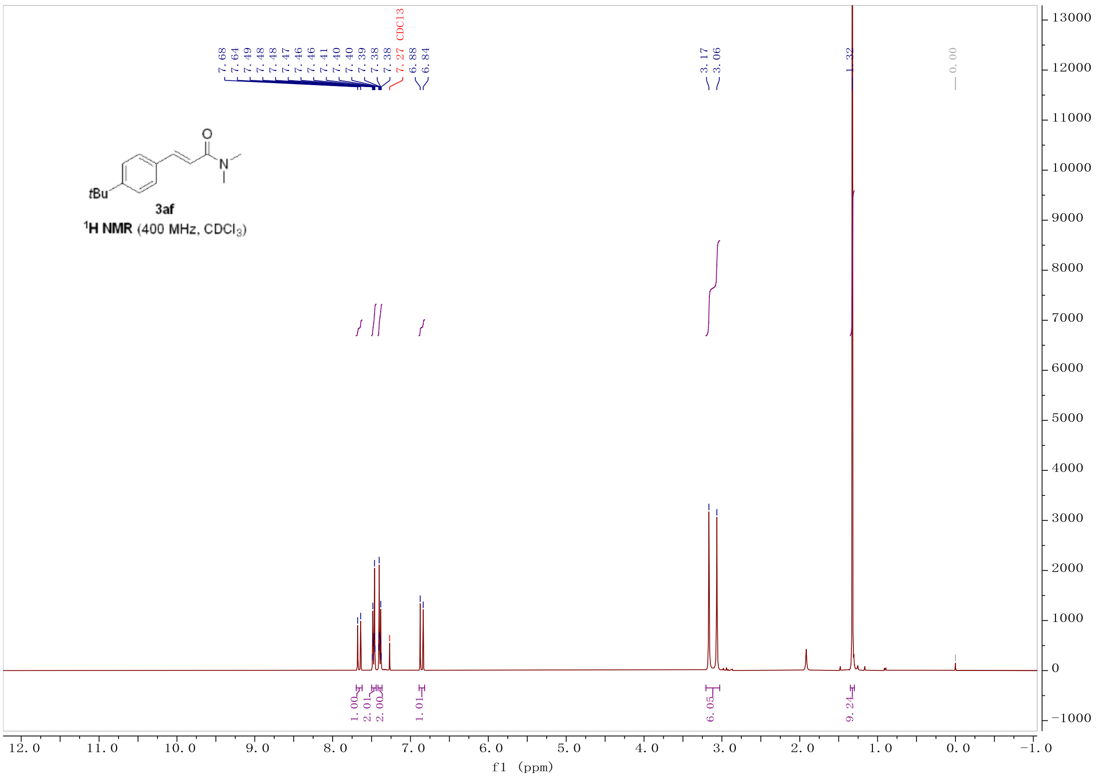
3af
^{1}H NMR (400 MHz, CDCl$_3$)
7.68 7.64 7.49 7.48 7.48 7.47 7.46 7.46 7.41 7.40 7.40 7.39 7.38 7.38 7.27 CDCl3 6.88 6.84
3.17 3.06
1.32
0.00
1.00 2.01 2.00 1.01 6.05 9.24
f1 (ppm)

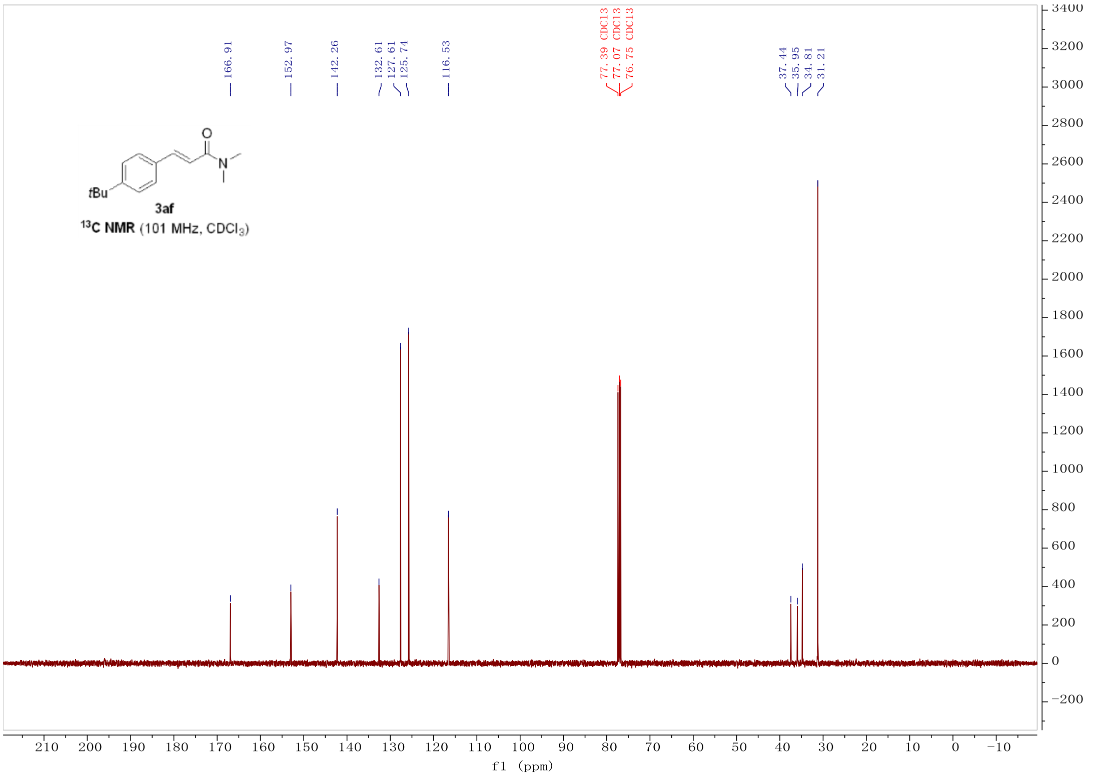
3af
^{13}C NMR (101 MHz, CDCl$_3$)
166.91
152.97
142.26
132.61 127.61 125.74
116.53
77.39 CDCl3 77.07 CDCl3 76.75 CDCl3
37.44 35.95 34.81 31.21
f1 (ppm)

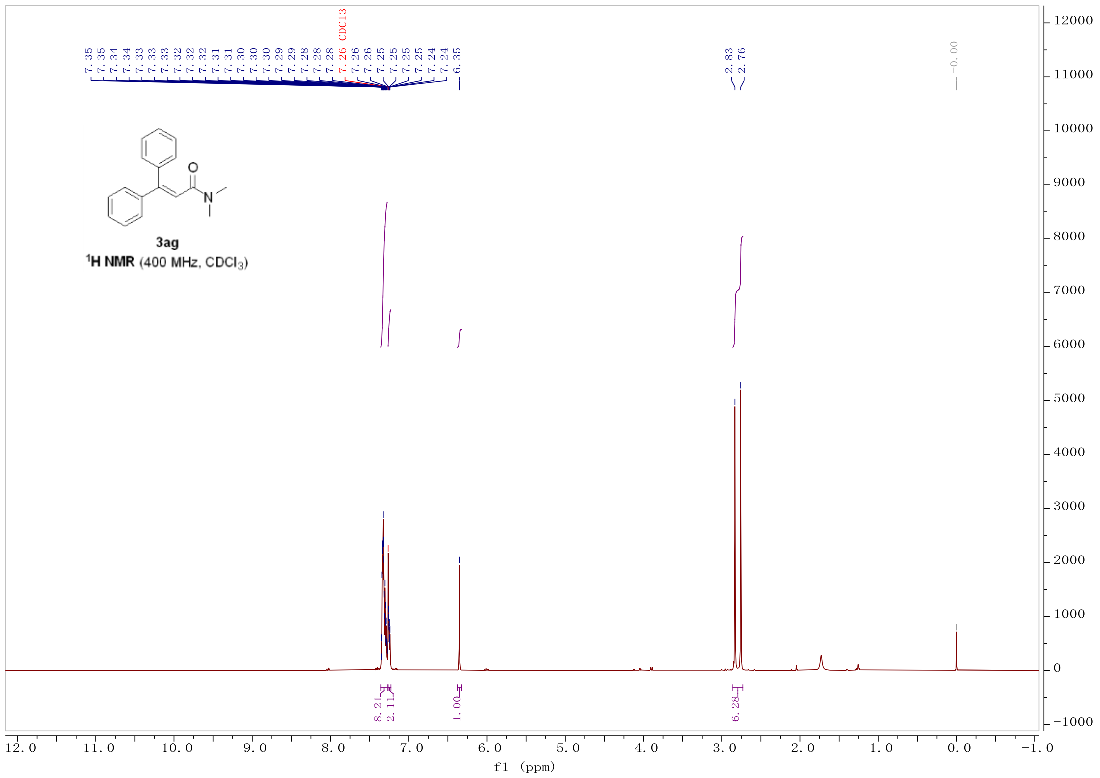
7.35 7.35 7.34 7.34 7.33 7.33 7.33 7.32 7.32 7.32 7.31 7.31 7.30 7.30 7.30 7.30 7.29 7.29 7.28 7.28 7.28 7.28 7.26 CDCl3 7.26 7.26 7.25 7.25 7.25 7.25 7.24 7.24 6.35
2.83 2.76
0.00
3ag
1H NMR (400 MHz, CDCl3)
8.21 2.11 1.00 6.28
f1 (ppm)

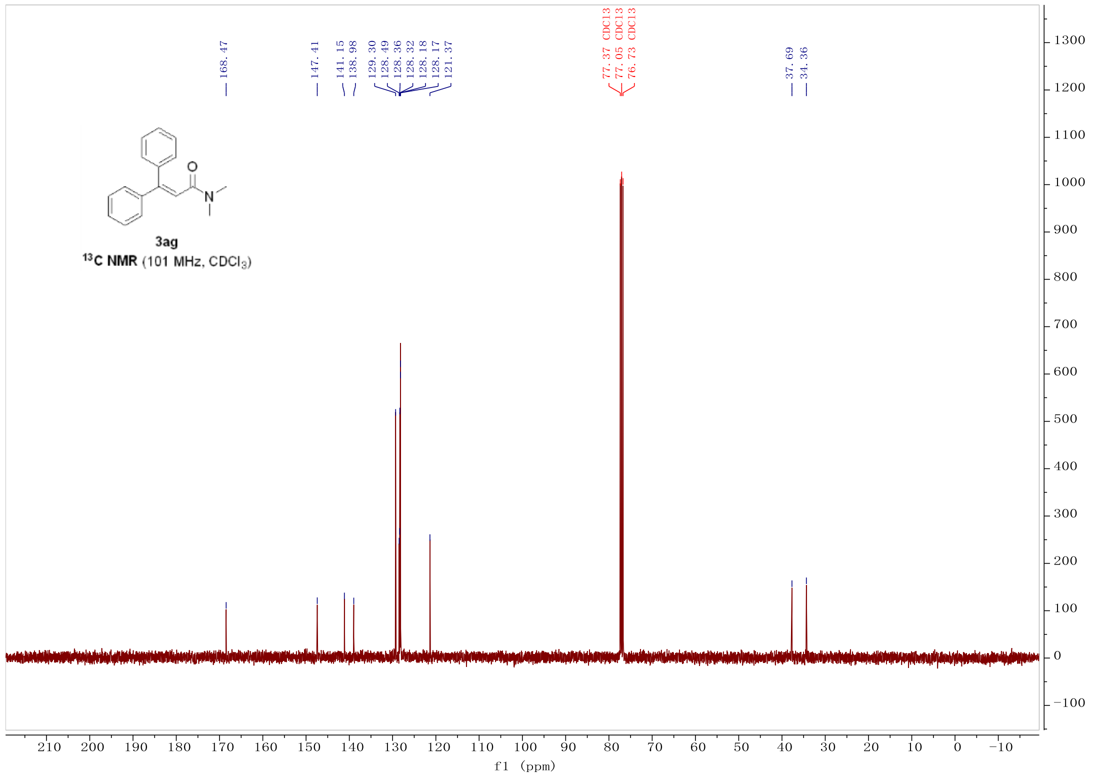
168.47
147.41 141.15 138.98 129.30 128.49 128.36 128.32 128.18 128.17 121.37
77.37 CDCl3 77.05 CDCl3 76.73 CDCl3
37.69 34.36
3ag
13C NMR (101 MHz, CDCl3)
f1 (ppm)

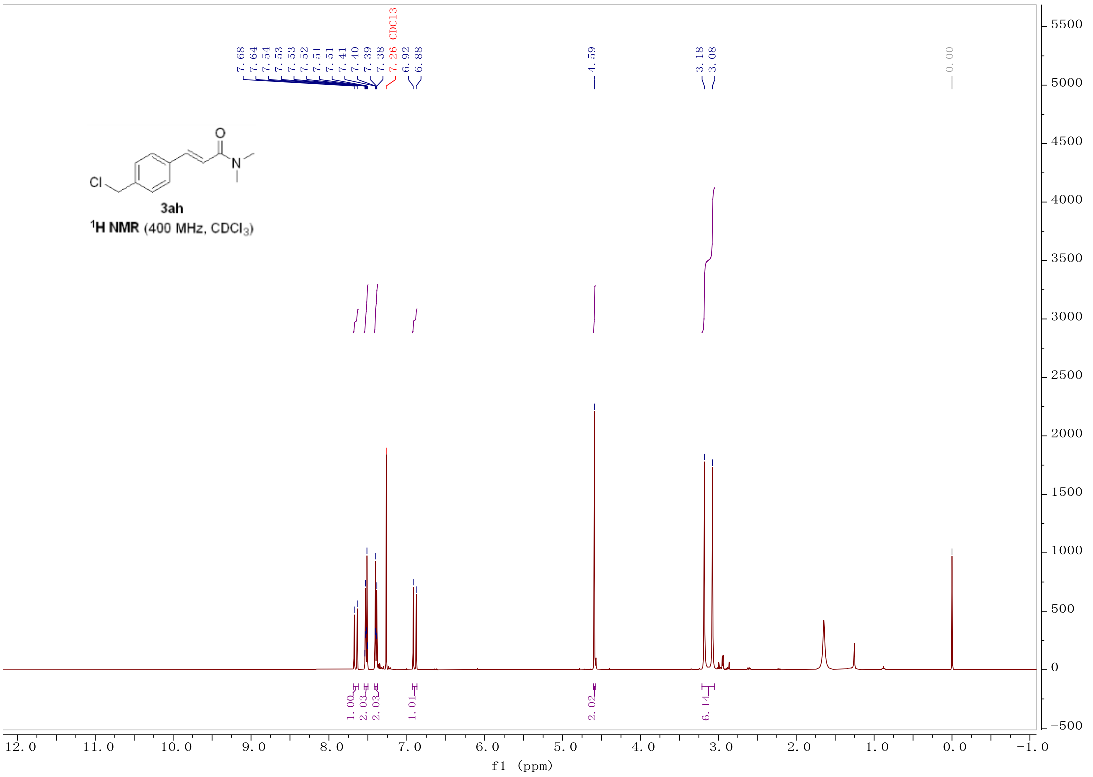
7.68
7.64
7.54
7.53
7.53
7.52
7.51
7.51
7.41
7.40
7.39
7.38
7.26 CDCl3
6.92
6.88
4.59
3.18
3.08
0.00
3ah
1H NMR (400 MHz, CDCl3)
1.00
2.03
2.03
1.01
2.02
6.14
f1 (ppm)

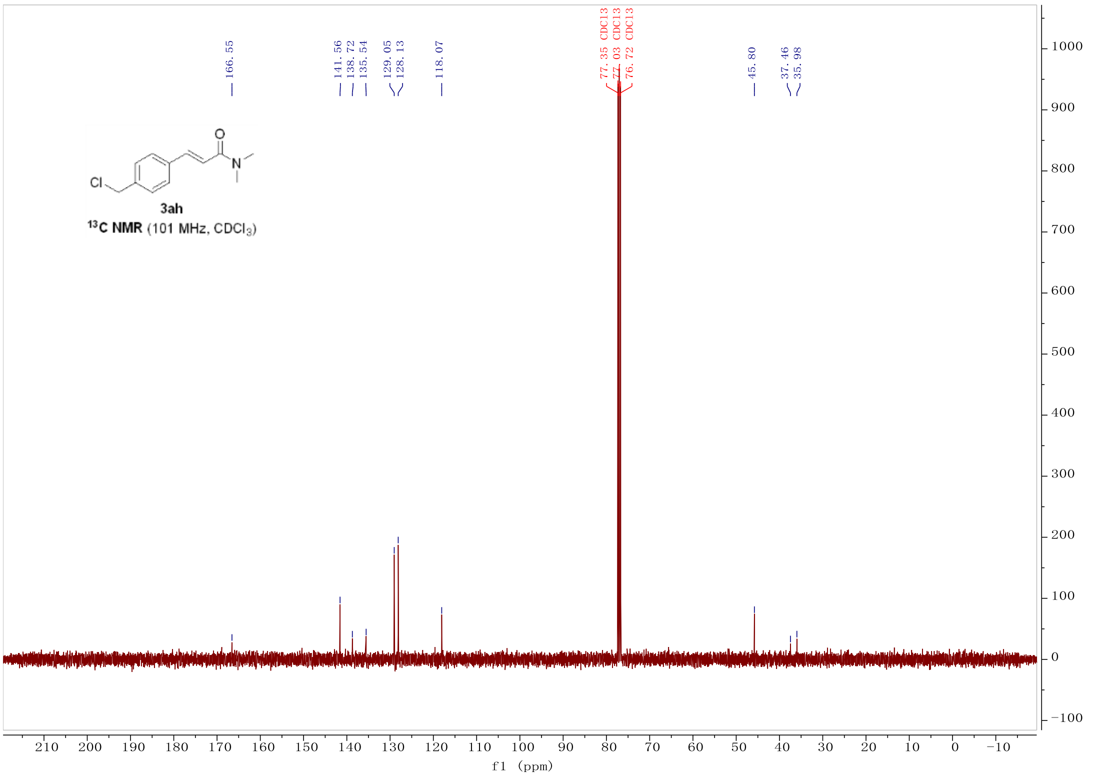
166.55
141.56
138.72
135.54
129.05
128.13
118.07
77.35 CDCl3
77.03 CDCl3
76.72 CDCl3
45.80
37.46
35.98
3ah
13C NMR (101 MHz, CDCl3)
f1 (ppm)

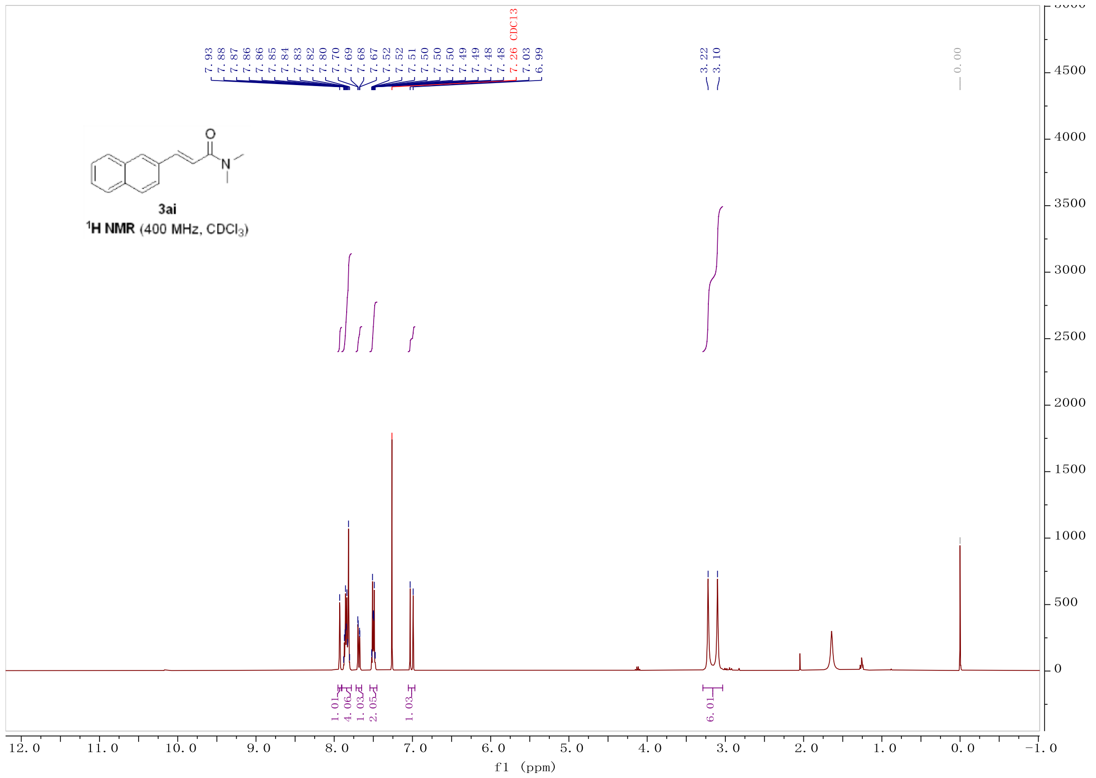
3ai
1H NMR (400 MHz, CDCl3)

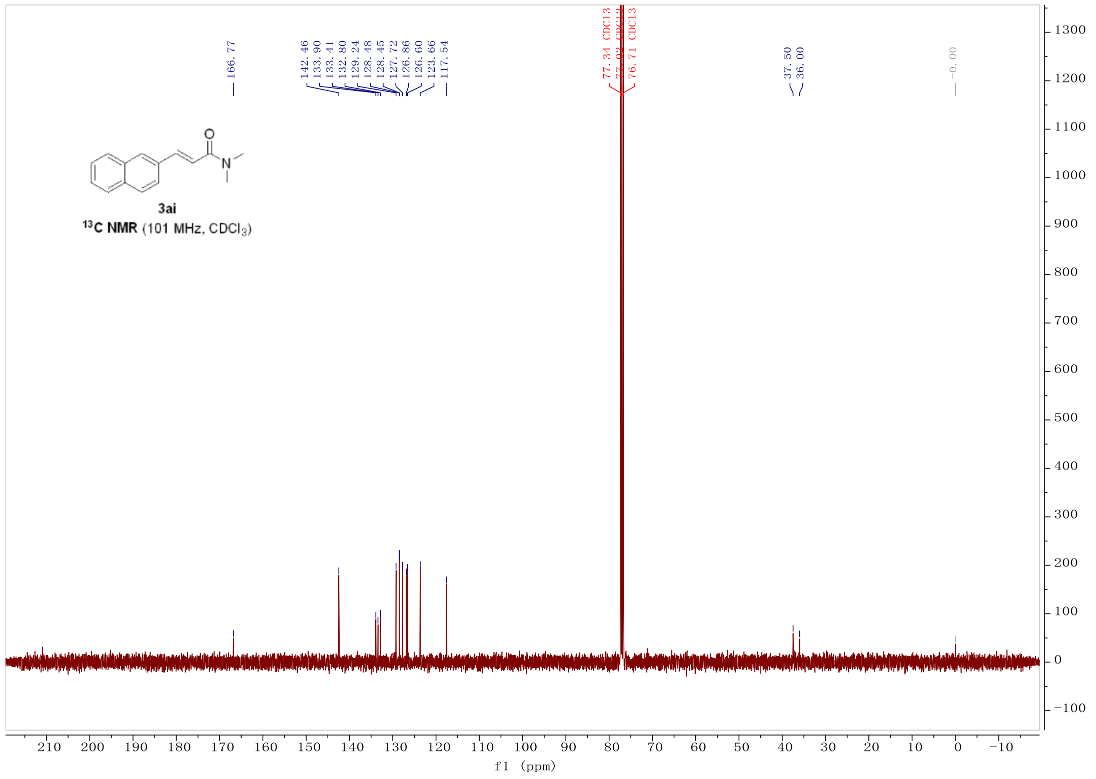
3ai
13C NMR (101 MHz, CDCl3)

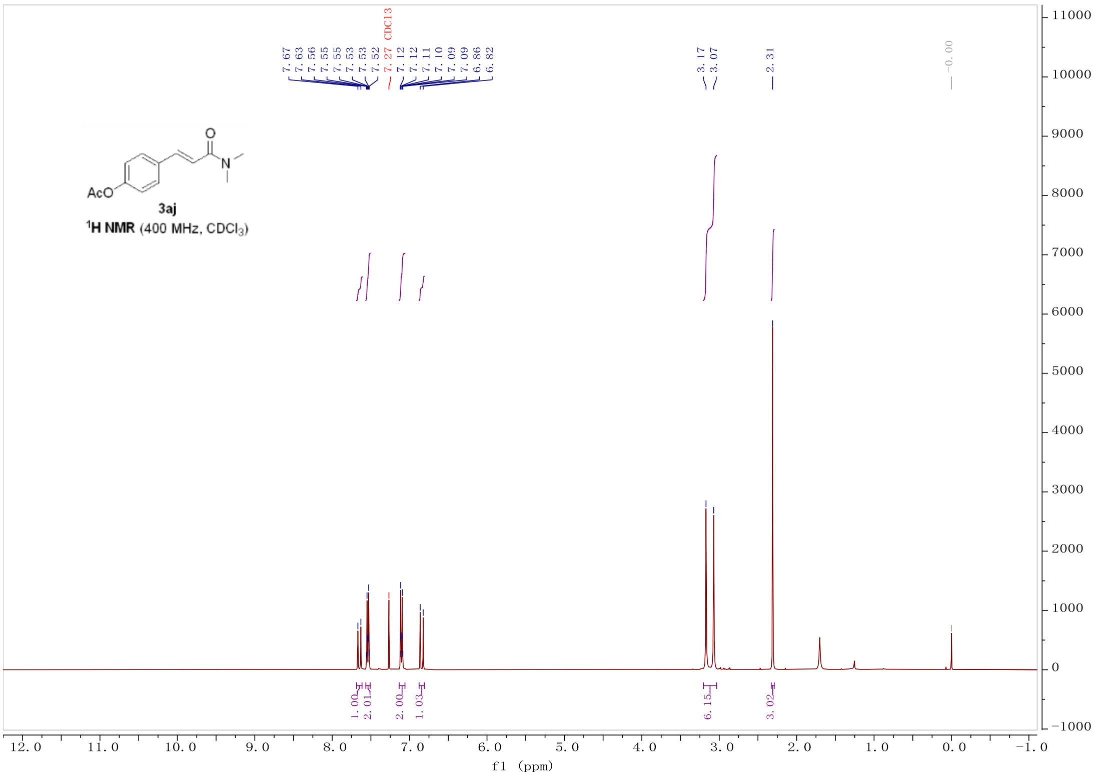
AcO
3aj
^{1}H NMR (400 MHz, CDCl$_3$)
7.67
7.63
7.56
7.55
7.55
7.53
7.53
7.52
7.27 CDCl3
7.12
7.12
7.11
7.10
7.09
7.09
6.86
6.82
3.17
3.07
2.31
0.00
1.00
2.01
2.00
1.03
6.15
3.02
f1 (ppm)

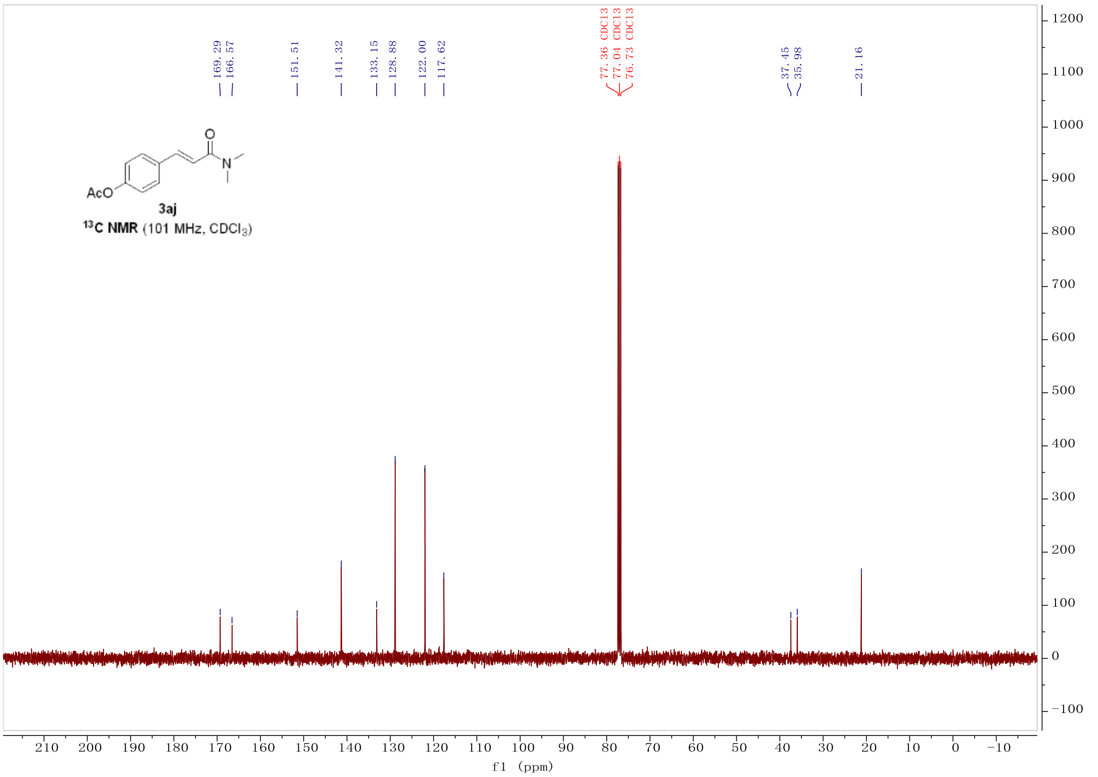
AcO
3aj
^{13}C NMR (101 MHz, CDCl$_3$)
169.29
166.57
151.51
141.32
133.15
128.88
122.00
117.62
77.36 CDCl3
77.04 CDCl3
76.73 CDCl3
37.45
35.98
21.16
f1 (ppm)

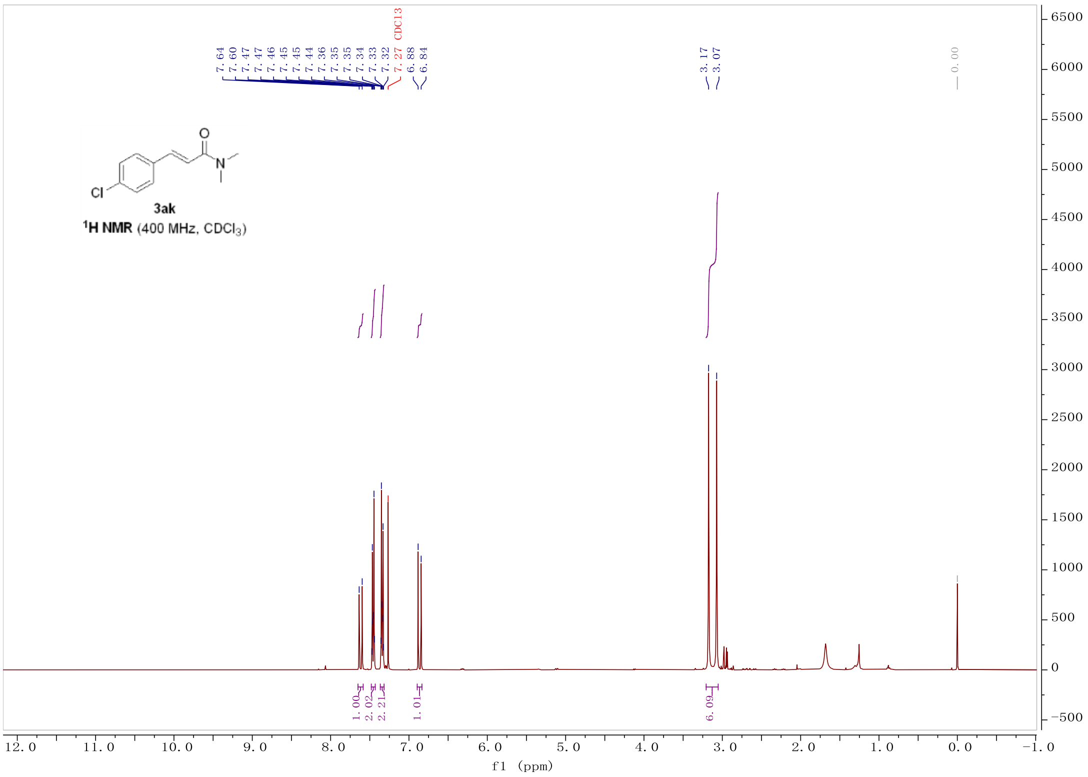
7.64
7.60
7.47
7.47
7.46
7.45
7.45
7.44
7.36
7.35
7.35
7.34
7.33
7.32
7.27 CDCl3
6.88
6.84
3.17
3.07
0.00
3ak
1H NMR (400 MHz, CDCl3)
1.00
2.02
2.21
1.01
6.09
f1 (ppm)

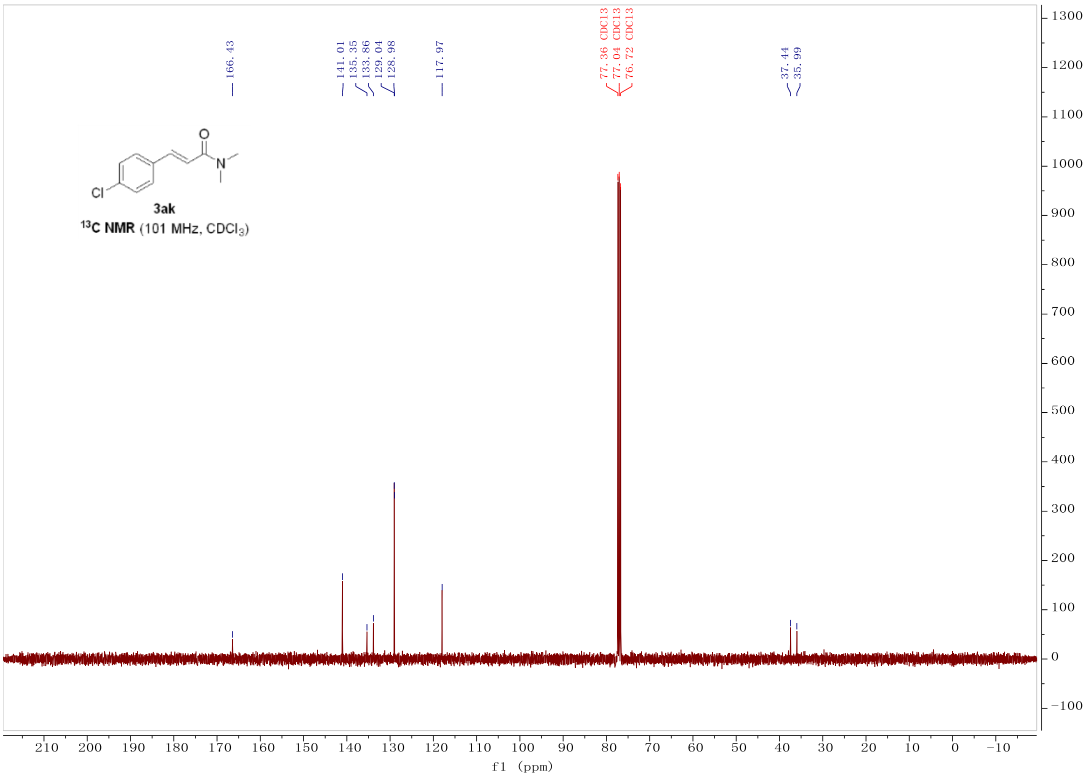
166.43
141.01
135.35
133.86
129.04
128.98
117.97
77.36 CDCl3
77.04 CDCl3
76.72 CDCl3
37.44
35.99
3ak
13C NMR (101 MHz, CDCl3)
f1 (ppm)

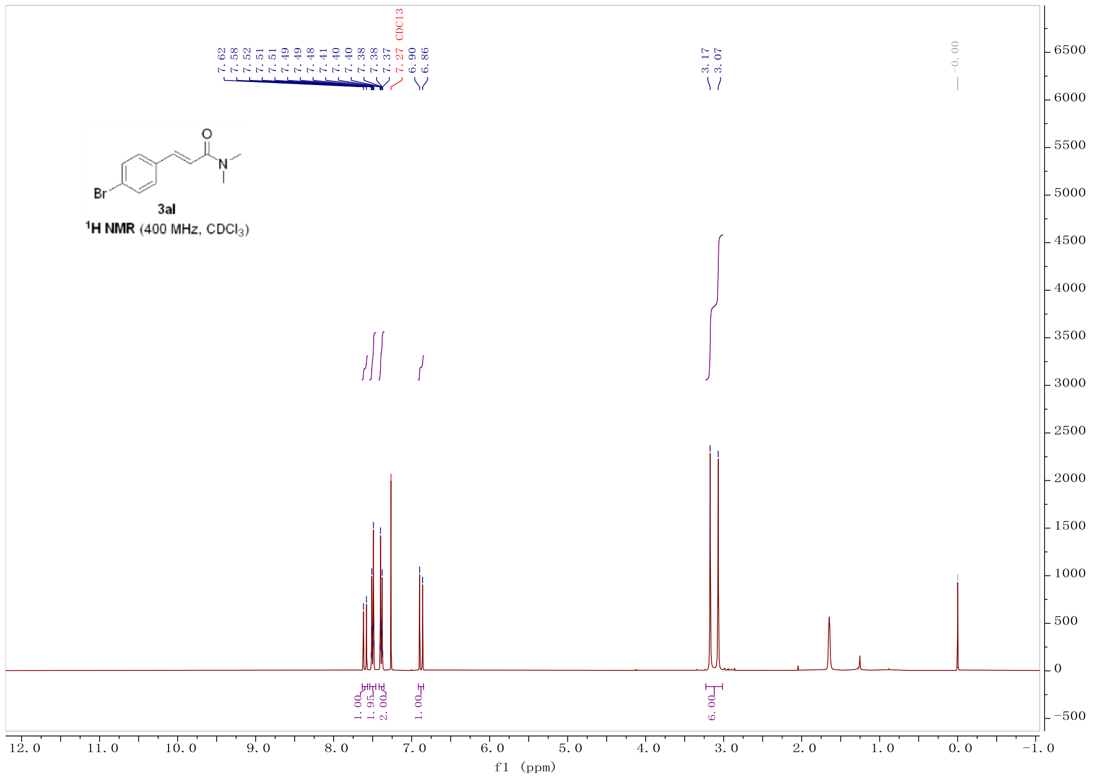
7.62
7.58
7.52
7.51
7.51
7.49
7.49
7.48
7.41
7.40
7.40
7.38
7.38
7.37
7.27 CDCl3
6.90
6.86
3.17
3.07
0.00
Br
O
N
3al
^{1}H NMR (400 MHz, CDCl$_3$)
1.00
1.95
2.00
1.00
6.00
f1 (ppm)

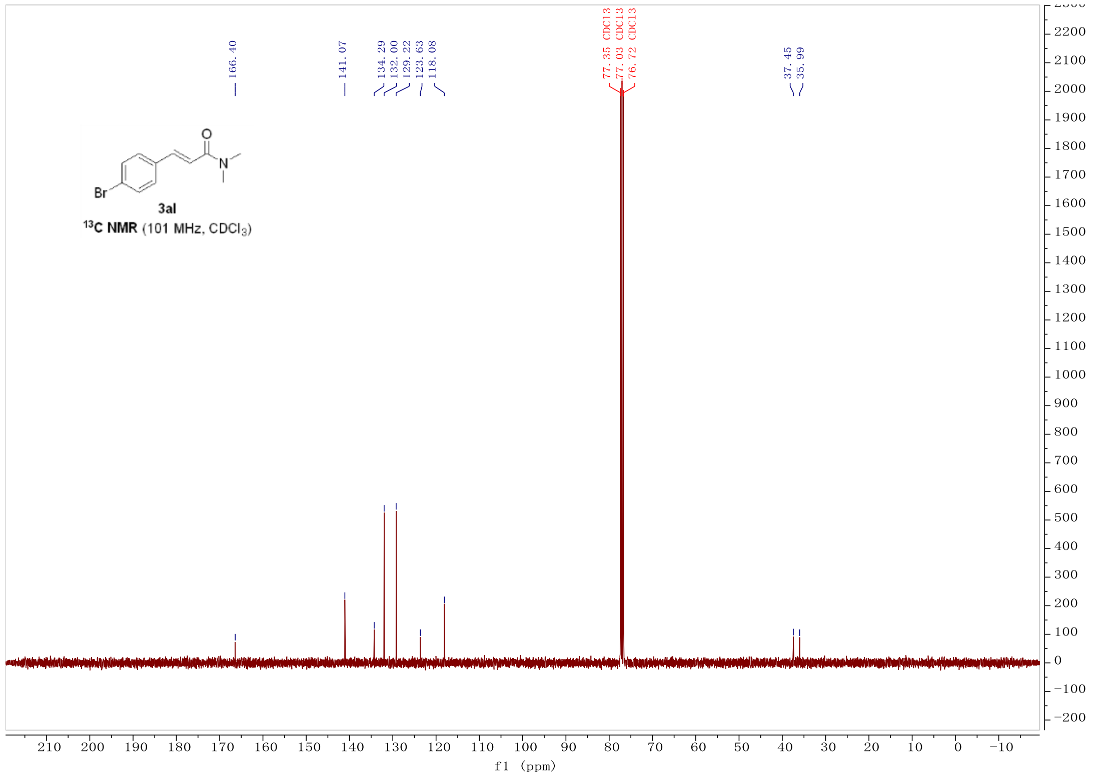
166.40
141.07
134.29
132.00
129.22
123.63
118.08
77.35 CDCl3
77.03 CDCl3
76.72 CDCl3
37.45
35.99
Br
O
N
3al
^{13}C NMR (101 MHz, CDCl$_3$)
f1 (ppm)

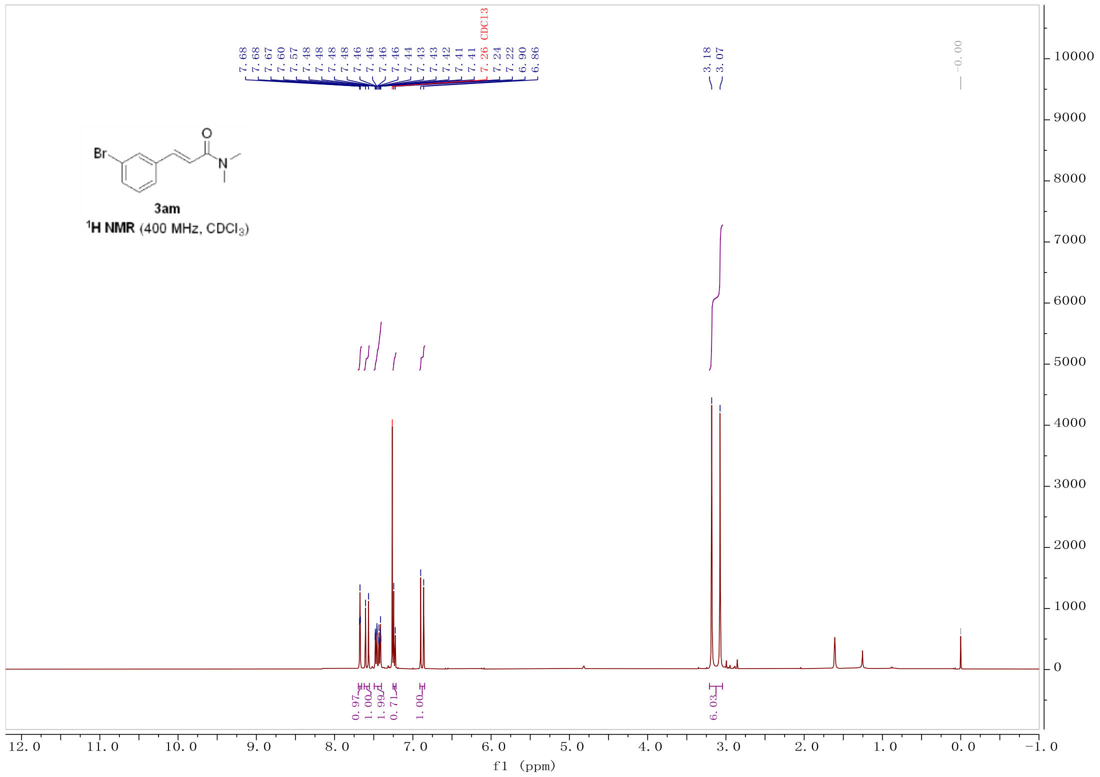
Br
O
N
3am
1H NMR (400 MHz, CDCl3)
7.68 7.68 7.67 7.60 7.57 7.48 7.48 7.48 7.48 7.46 7.46 7.46 7.46 7.44 7.43 7.43 7.42 7.41 7.41 7.26 CDCl3 7.24 7.22 6.90 6.86
3.18 3.07
-0.00
0.97 1.00 1.99 0.71 1.00
6.03
f1 (ppm)

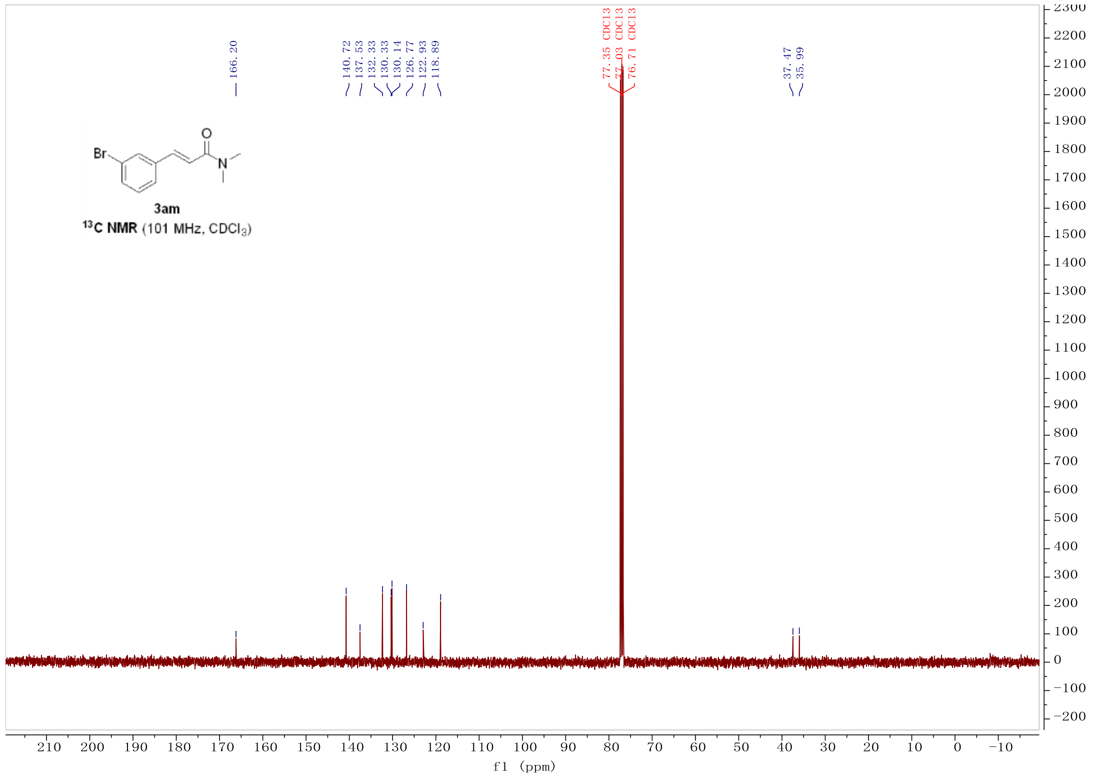
Br
O
N
3am
13C NMR (101 MHz, CDCl3)
166.20
140.72 137.53 132.33 130.33 130.14 126.77 122.93 118.89
77.35 CDCl3 77.03 CDCl3 76.71 CDCl3
37.47 35.99
f1 (ppm)

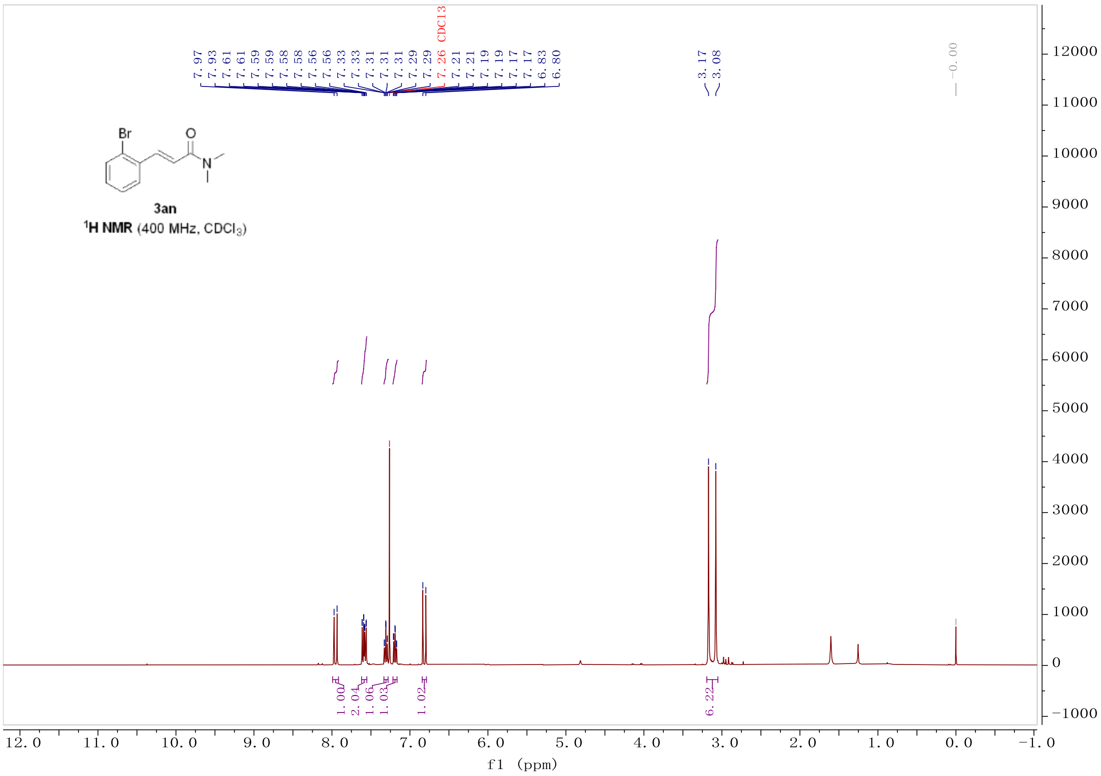
3an
1H NMR (400 MHz, CDCl3)
f1 (ppm)

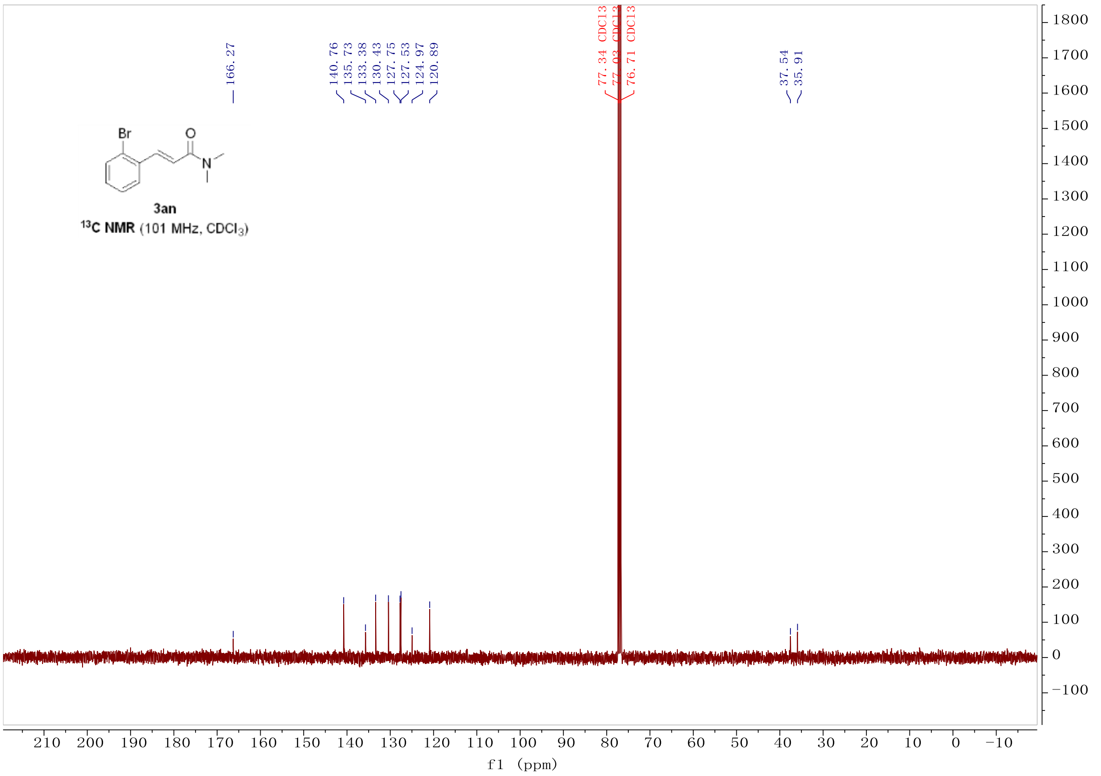
3an
13C NMR (101 MHz, CDCl3)
f1 (ppm)

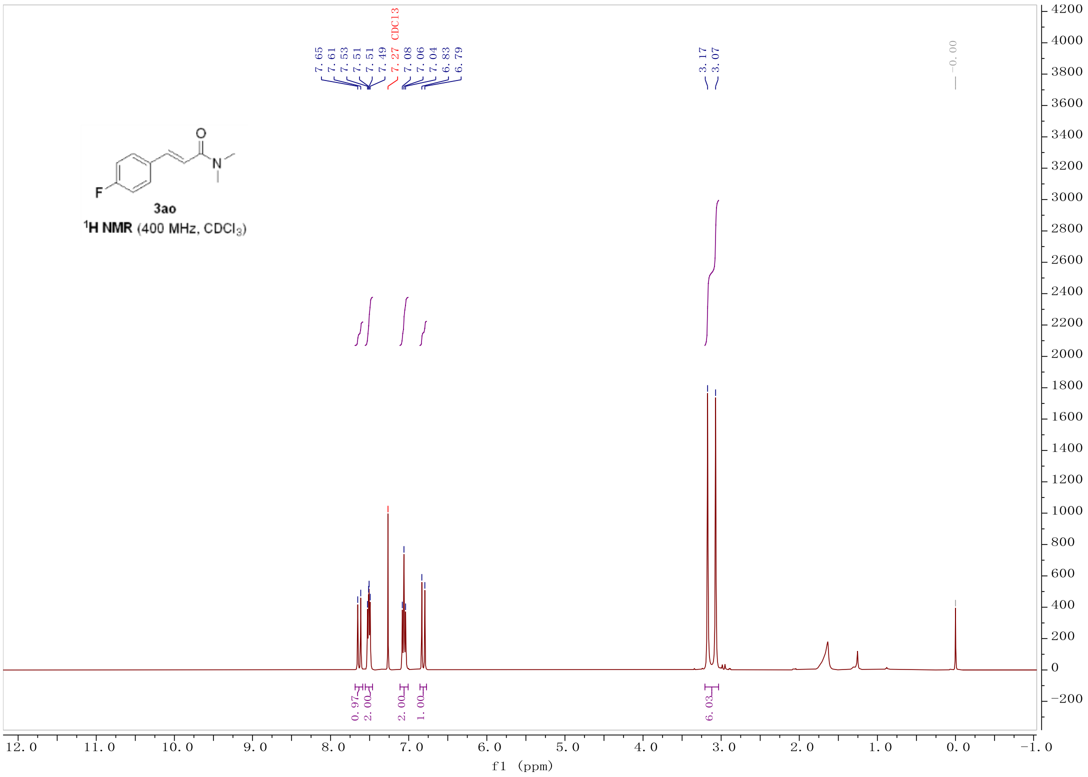
7.65
7.61
7.53
7.51
7.51
7.49
7.27 CDCl3
7.08
7.06
7.04
6.83
6.79
3.17
3.07
0.00
O
N
F
3ao
1H NMR (400 MHz, CDCl3)
0.97
2.00
2.00
1.00
6.03
f1 (ppm)

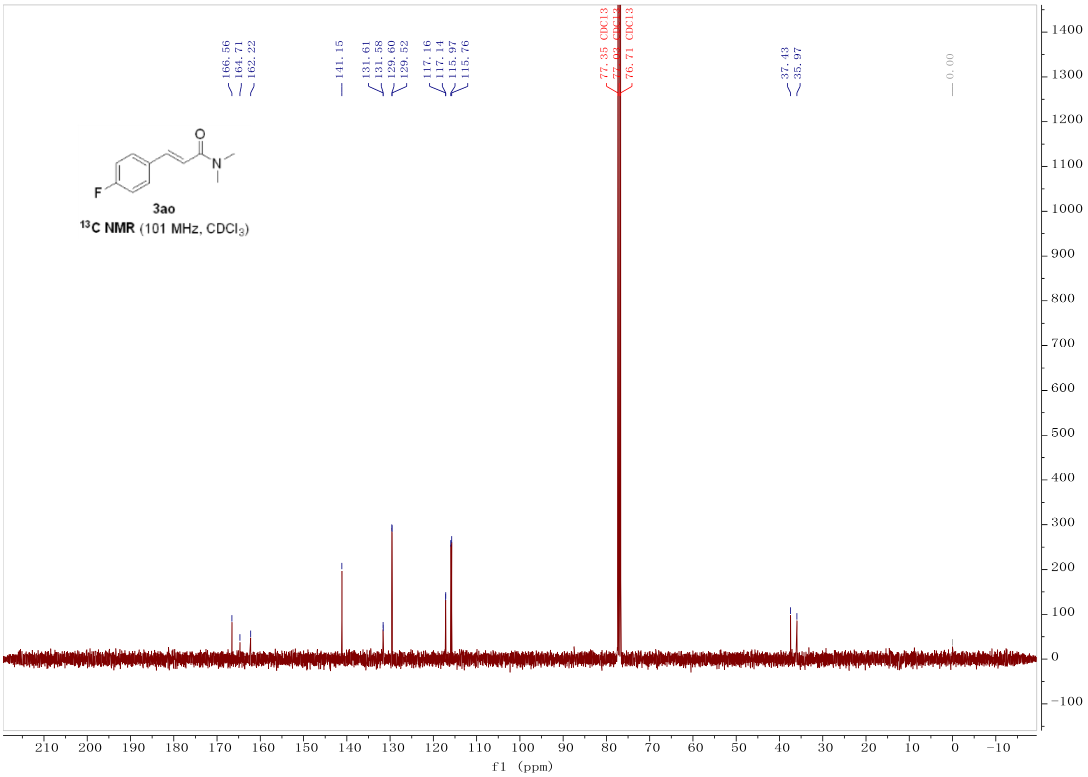
166.56
164.71
162.22
141.15
131.61
131.58
129.60
129.52
117.16
117.14
115.97
115.76
77.35 CDCl3
77.03 CDCl3
76.71 CDCl3
37.43
35.97
0.00
O
N
F
3ao
13C NMR (101 MHz, CDCl3)
f1 (ppm)

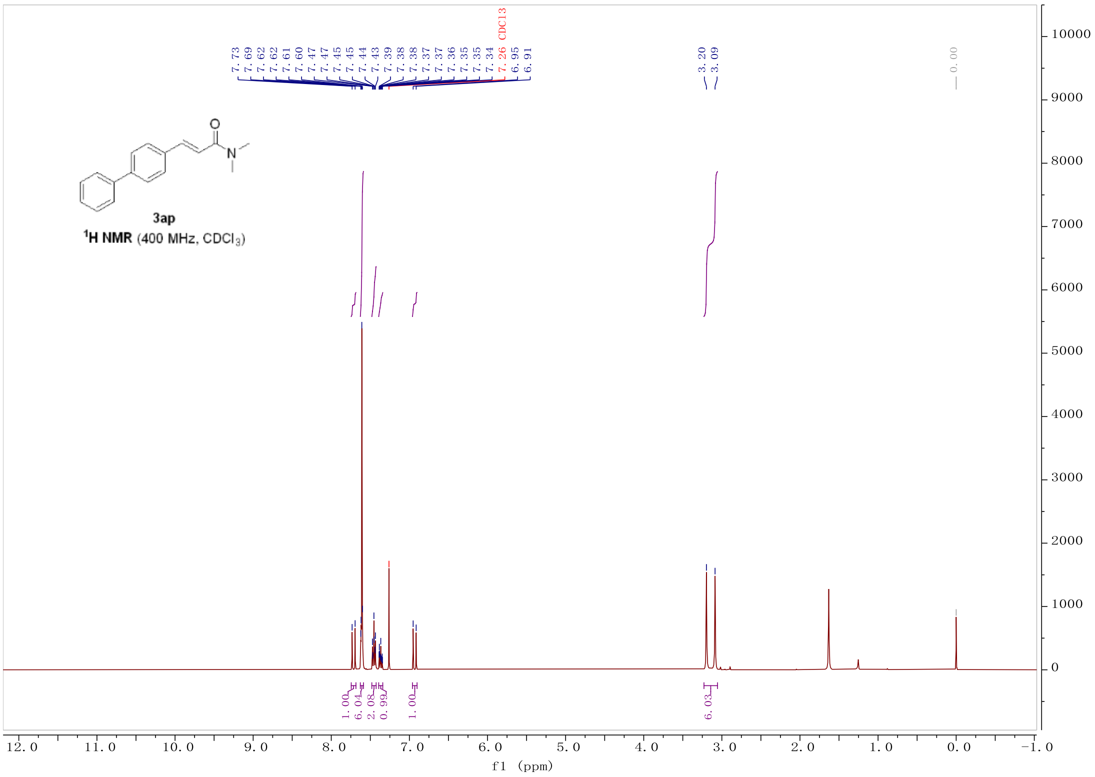
3ap
1H NMR (400 MHz, CDCl3)
7.73
7.69
7.62
7.62
7.61
7.60
7.47
7.47
7.45
7.44
7.43
7.39
7.38
7.38
7.37
7.37
7.36
7.35
7.35
7.34
7.26 CDCl3
6.95
6.91
3.20
3.09
0.00
1.00
6.04
2.08
0.99
1.00
6.03
f1 (ppm)

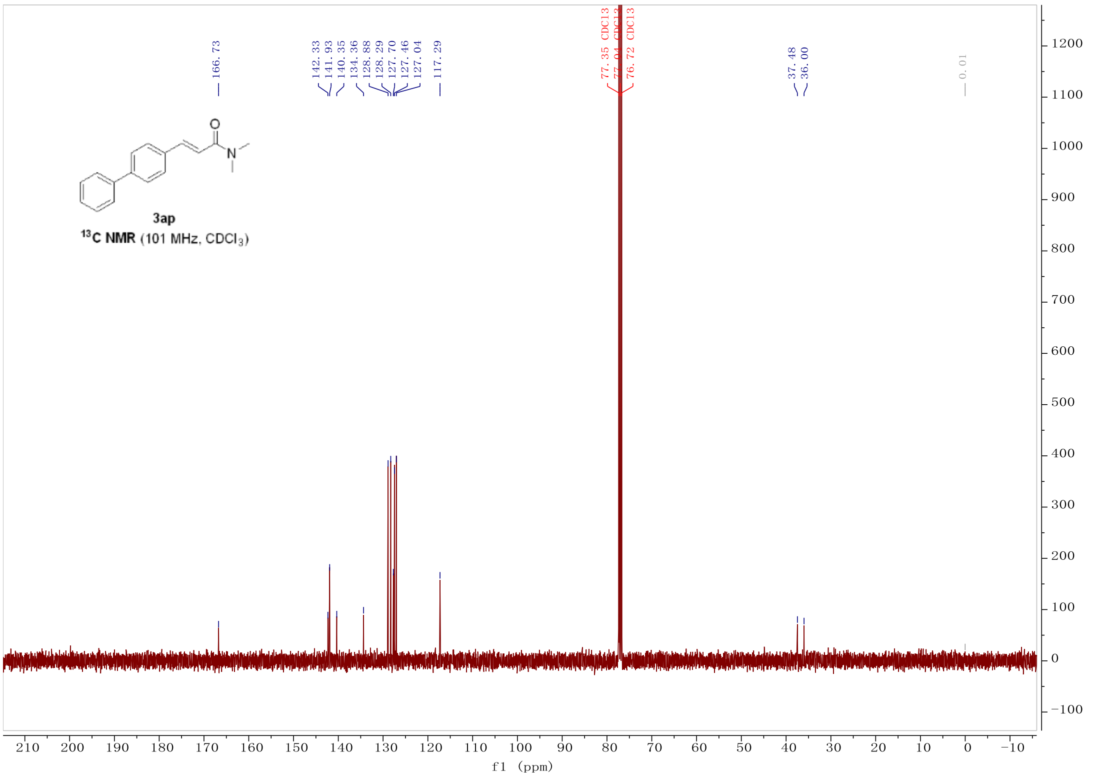
3ap
13C NMR (101 MHz, CDCl3)
166.73
142.33
141.93
140.35
134.36
128.88
128.29
127.70
127.46
127.04
117.29
77.35 CDCl3
77.04 CDCl3
76.72 CDCl3
37.48
36.00
0.01
f1 (ppm)

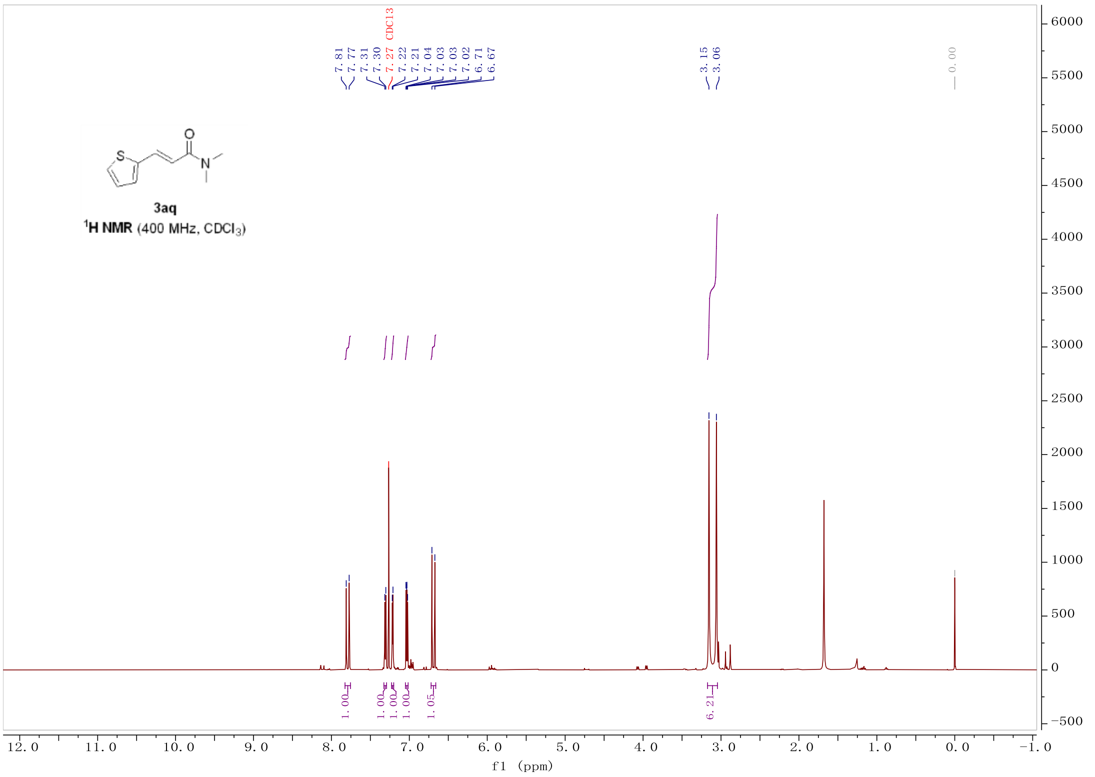
3aq
^{1}H NMR (400 MHz, CDCl$_3$)
7.81
7.77
7.31
7.30
7.27 CDCl3
7.22
7.21
7.04
7.03
7.03
7.02
6.71
6.67
3.15
3.06
0.00
1.00
1.00
1.00
1.00
1.05
6.21
f1 (ppm)

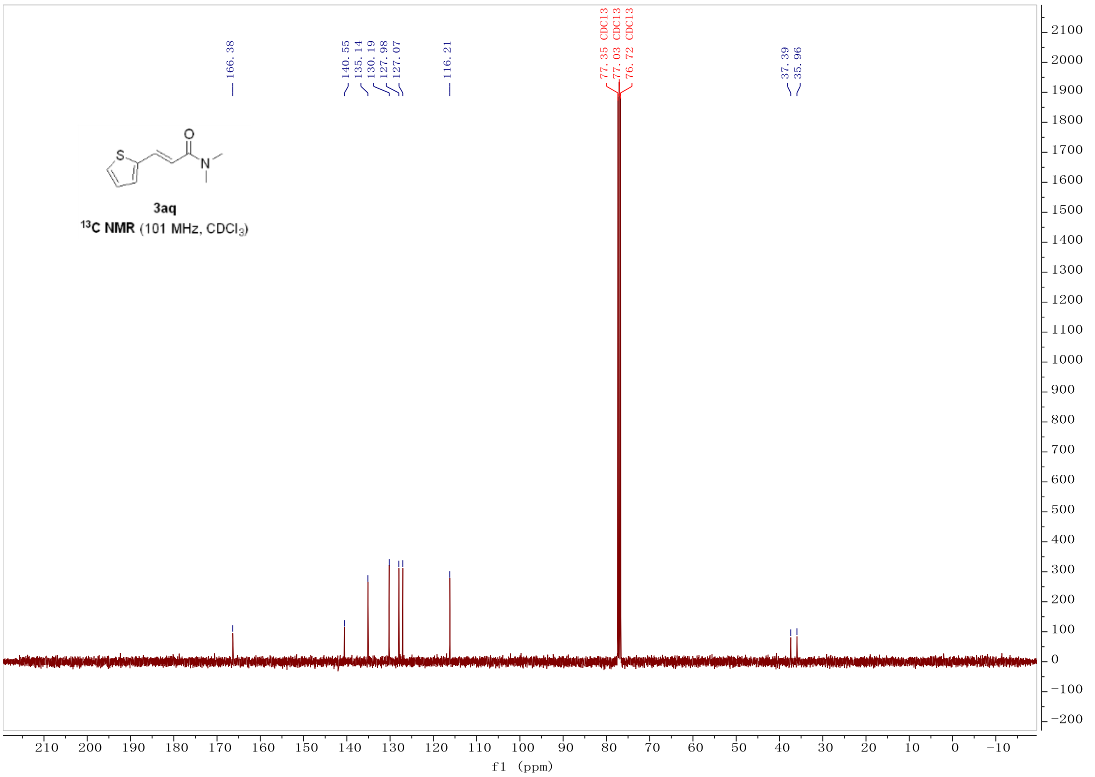
3aq
^{13}C NMR (101 MHz, CDCl$_3$)
166.38
140.55
135.14
130.19
127.98
127.07
116.21
77.35 CDCl3
77.03 CDCl3
76.72 CDCl3
37.39
35.96
f1 (ppm)

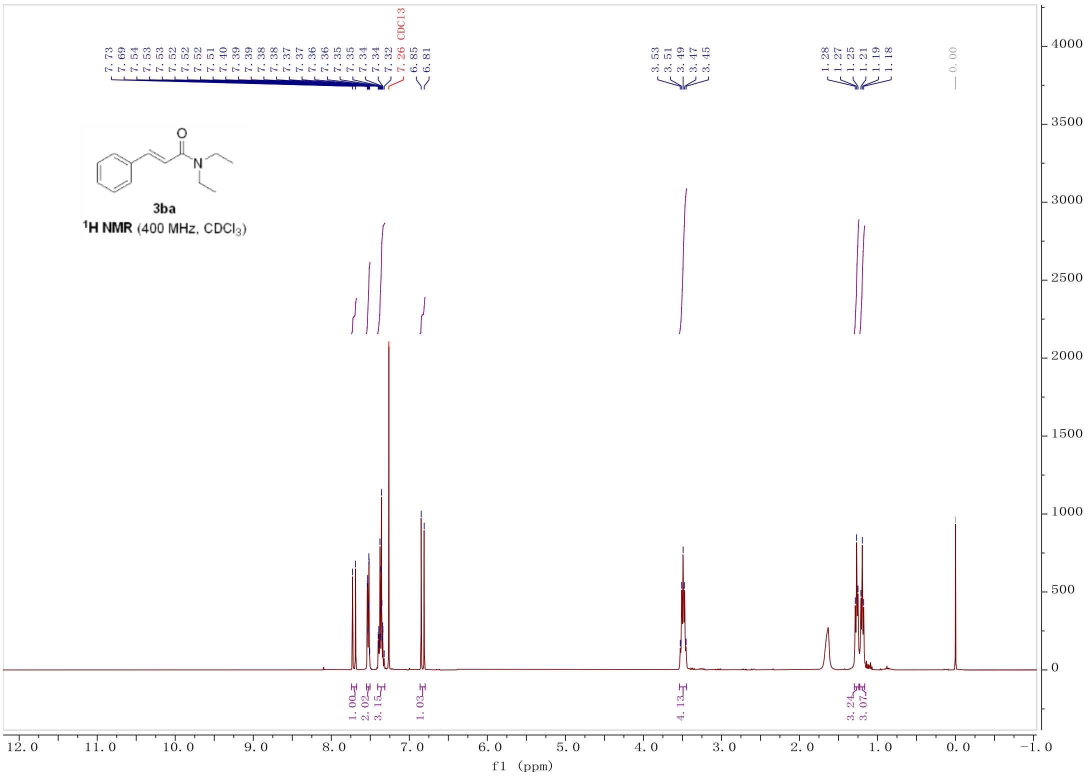
3ba
^{1}H NMR (400 MHz, CDCl$_3$)
f1 (ppm)

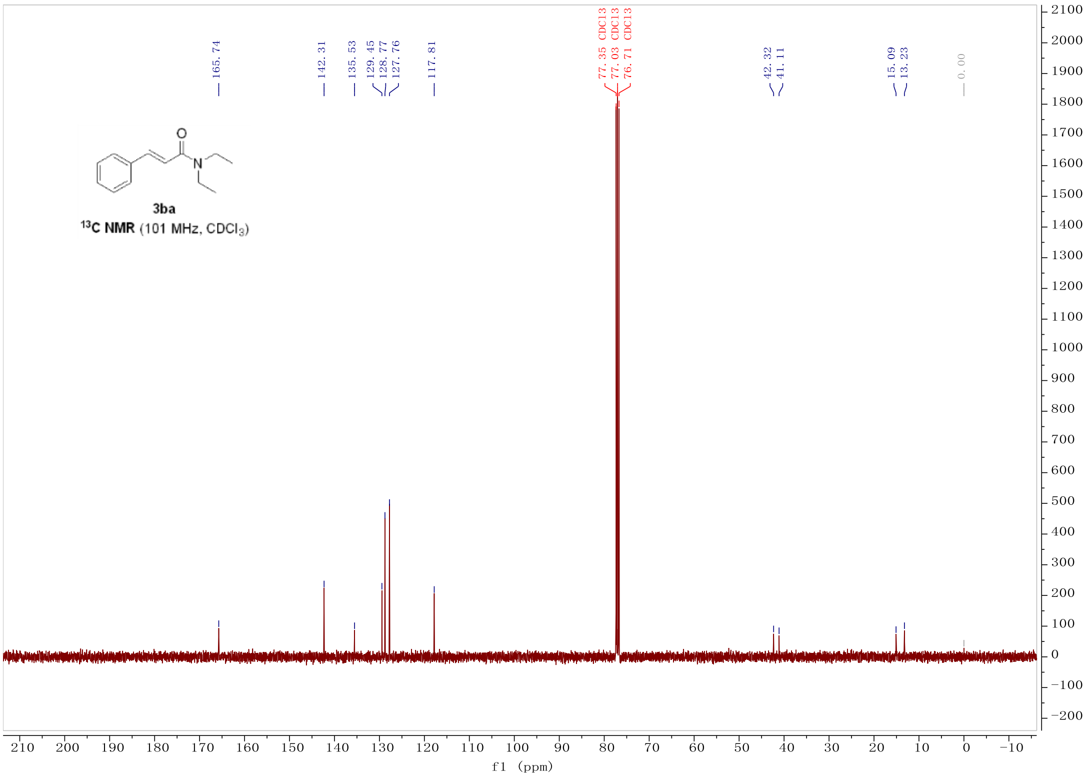
3ba
^{13}C NMR (101 MHz, CDCl$_3$)
165.74
142.31
135.53
129.45
128.77
127.76
117.81
77.35 CDCl3
77.03 CDCl3
76.71 CDCl3
42.32
41.11
15.09
13.23
0.00
f1 (ppm)

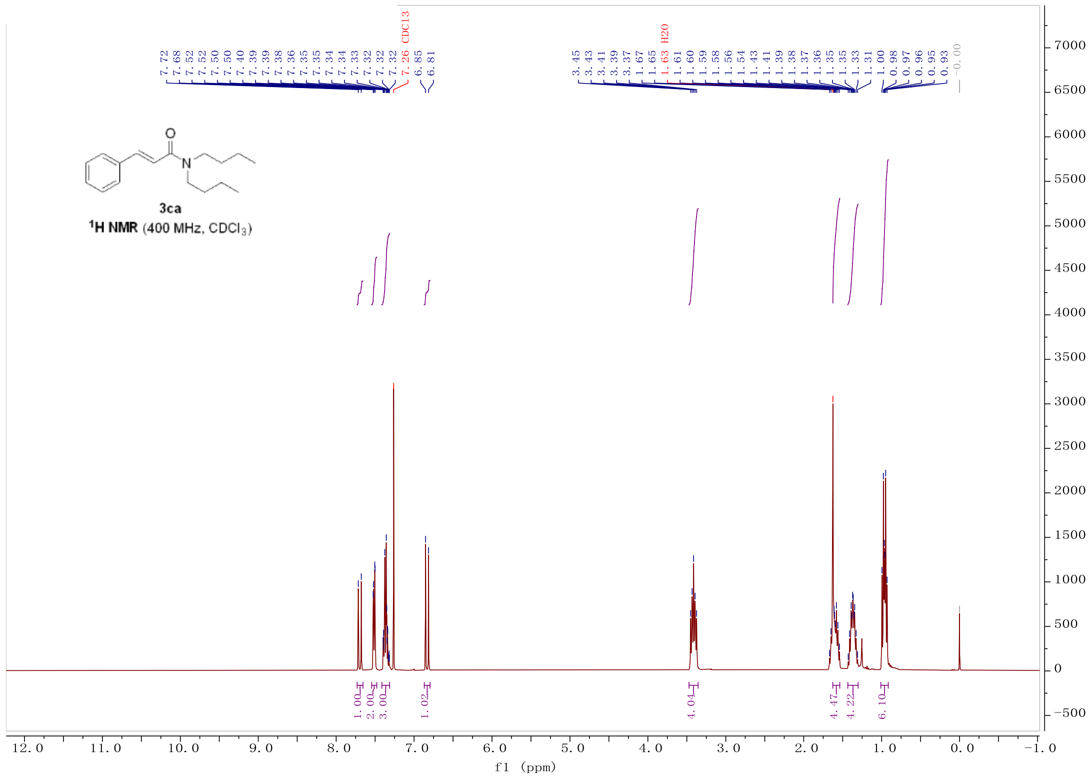
3ca
^{1}H NMR (400 MHz, CDCl$_3$)
f1 (ppm)

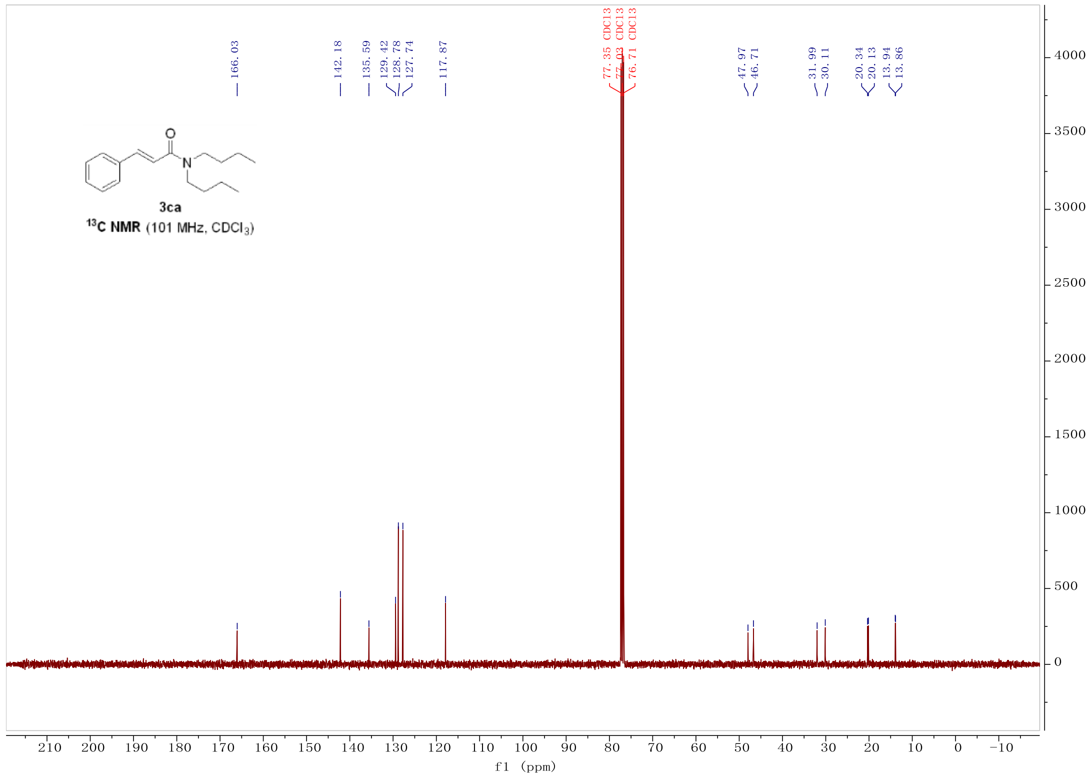
3ca
^{13}C NMR (101 MHz, CDCl$_3$)
f1 (ppm)

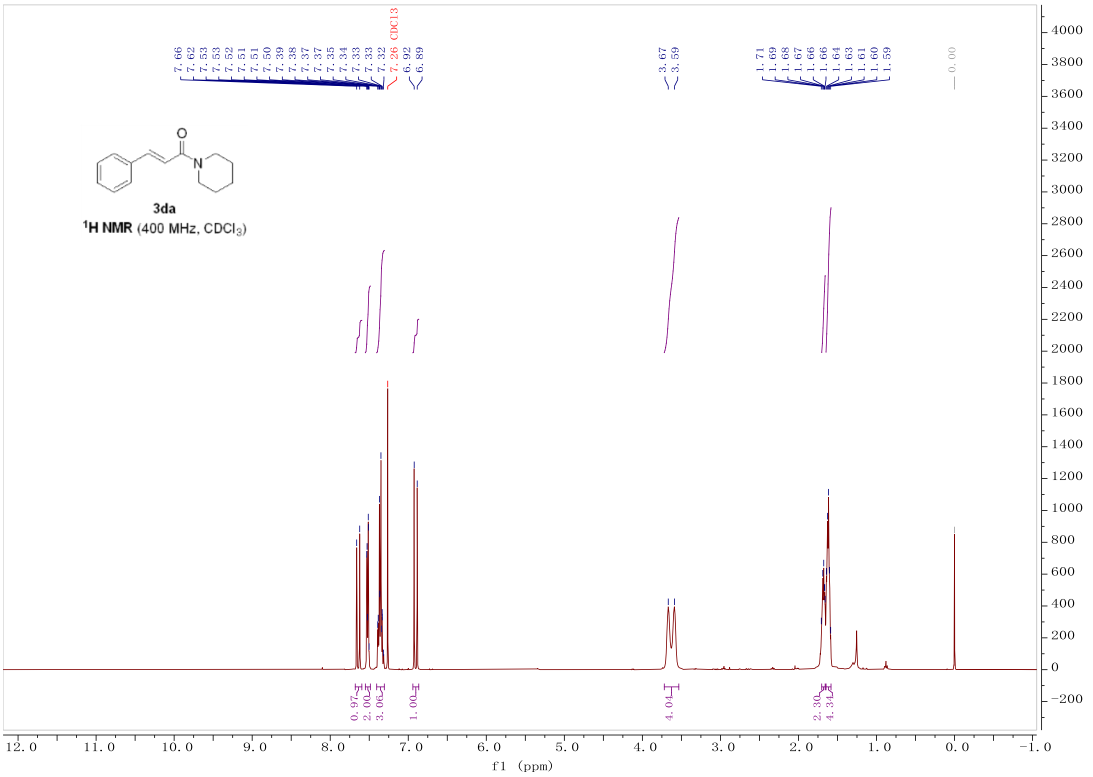
7.66
7.62
7.53
7.53
7.52
7.51
7.51
7.50
7.39
7.38
7.37
7.37
7.35
7.34
7.33
7.33
7.32
7.26 CDCl3
6.92
6.89
3.67
3.59
1.71
1.69
1.68
1.67
1.66
1.66
1.64
1.63
1.61
1.60
1.59
0.00
3da
1H NMR (400 MHz, CDCl3)
0.97
2.00
3.06
1.00
4.04
2.30
4.34
f1 (ppm)

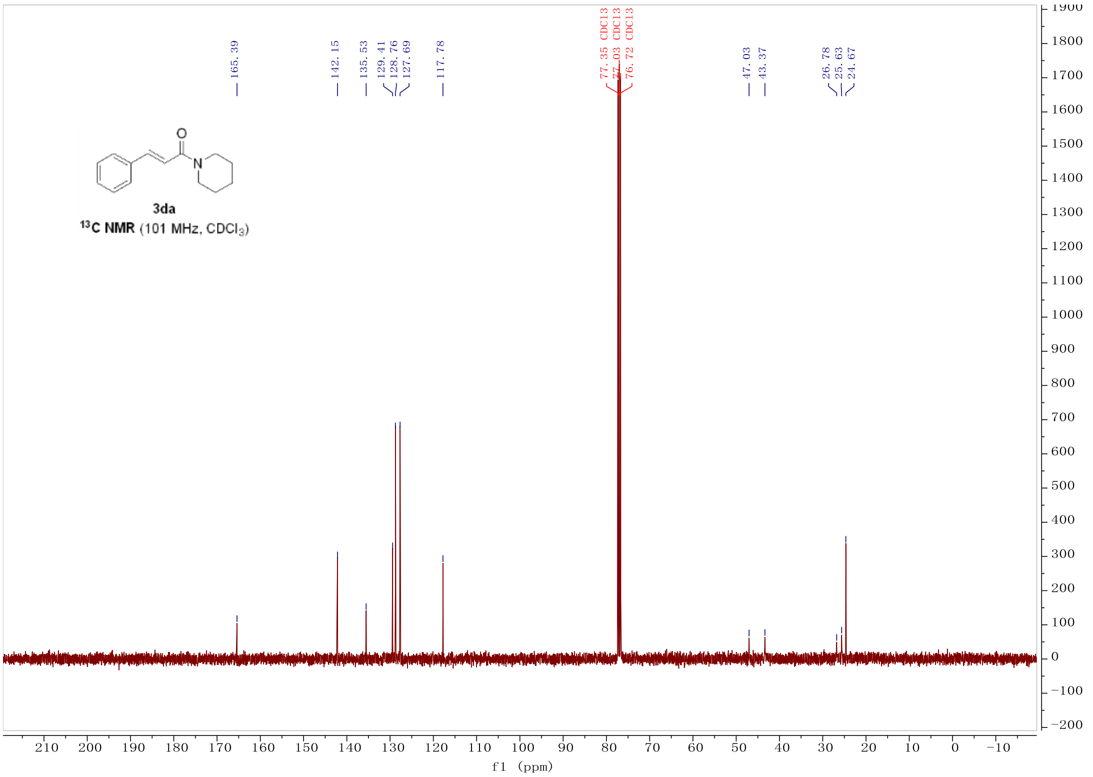
165.39
142.15
135.53
129.41
128.76
127.69
117.78
77.35 CDCl3
77.03 CDCl3
76.72 CDCl3
47.03
43.37
26.78
25.63
24.67
3da
13C NMR (101 MHz, CDCl3)
f1 (ppm)

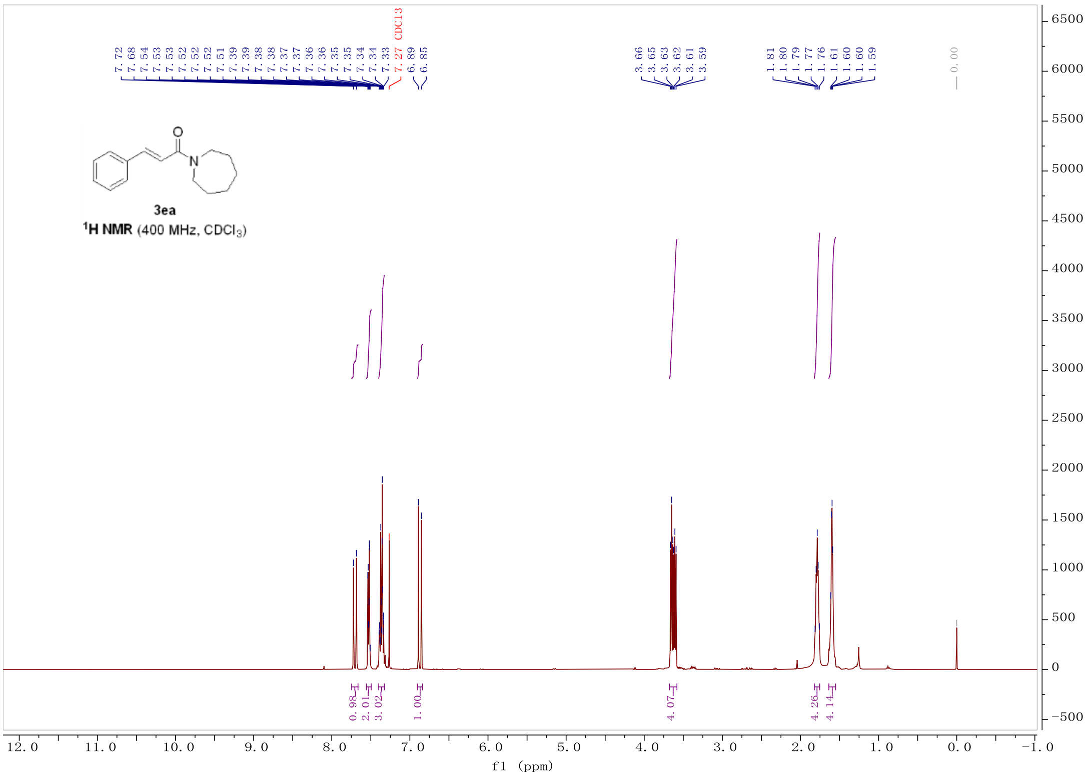
7.72 7.68 7.54 7.53 7.53 7.52 7.52 7.52 7.51 7.39 7.39 7.38 7.38 7.37 7.37 7.36 7.36 7.35 7.35 7.34 7.34 7.33
7.27 CDCl3
6.89 6.85
3.66 3.65 3.63 3.62 3.61 3.59
1.81 1.80 1.79 1.77 1.76 1.61 1.60 1.60 1.59
0.00
O
N
3ea
1H NMR (400 MHz, CDCl3)
0.98 2.01 3.02 1.00 4.07 4.26 4.14
f1 (ppm)

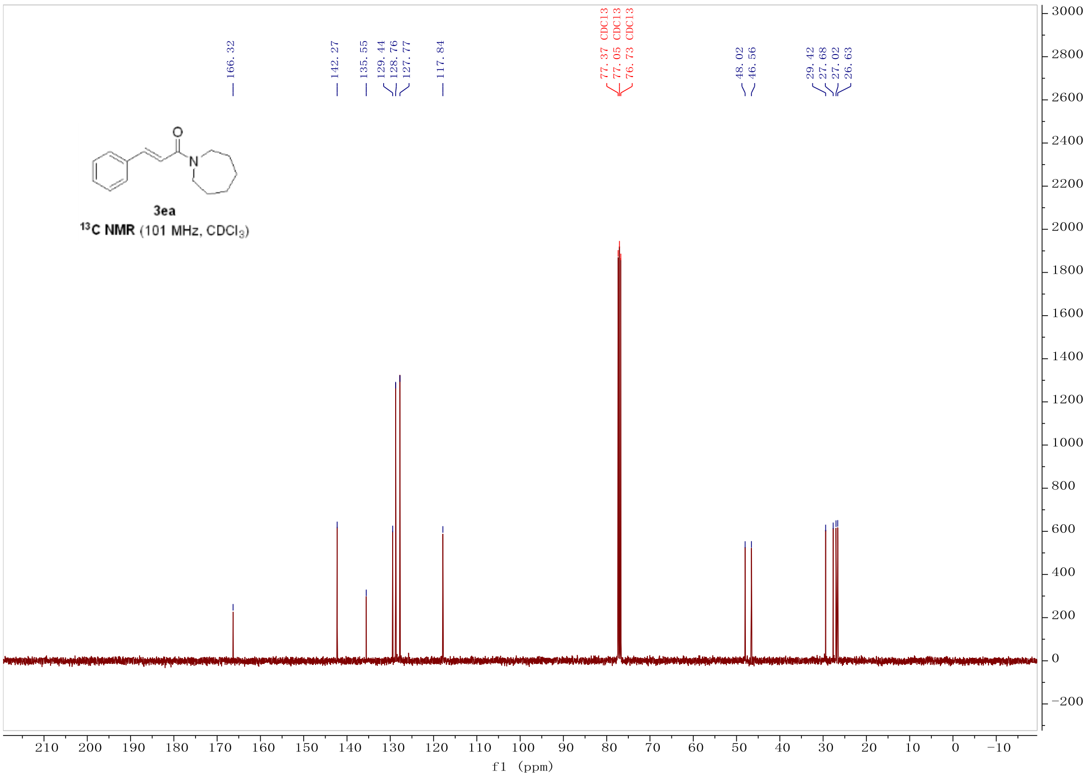
166.32
142.27 135.55 129.44 128.76 127.77
117.84
77.37 CDCl3 77.05 CDCl3 76.73 CDCl3
48.02 46.56
29.42 27.68 27.02 26.63
O
N
3ea
13C NMR (101 MHz, CDCl3)
f1 (ppm)

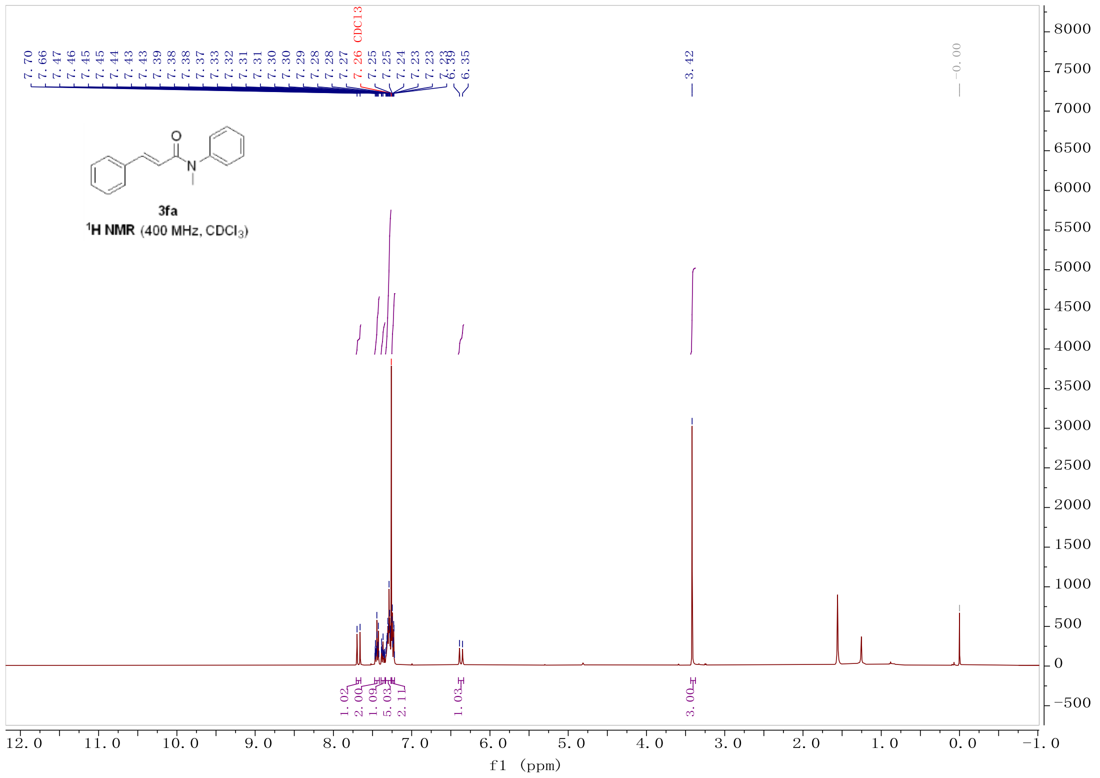
3fa
^{1}H NMR (400 MHz, $CDCl_3$)
f1 (ppm)

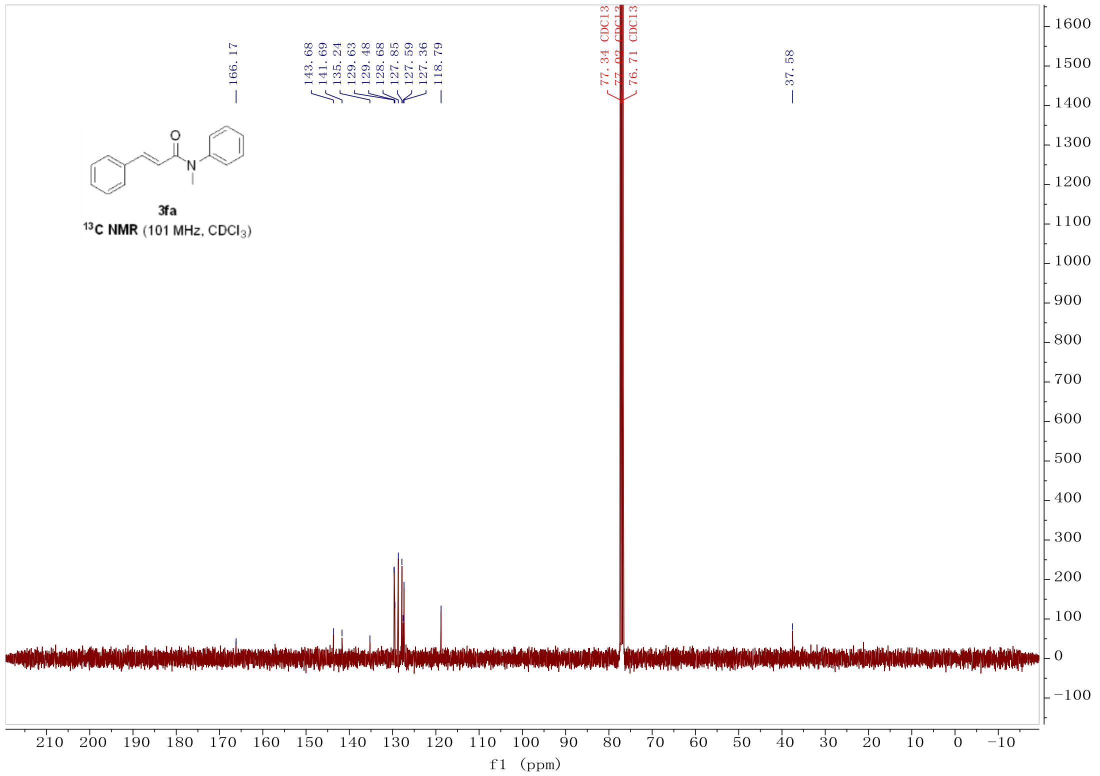
3fa
^{13}C NMR (101 MHz, $CDCl_3$)
f1 (ppm)

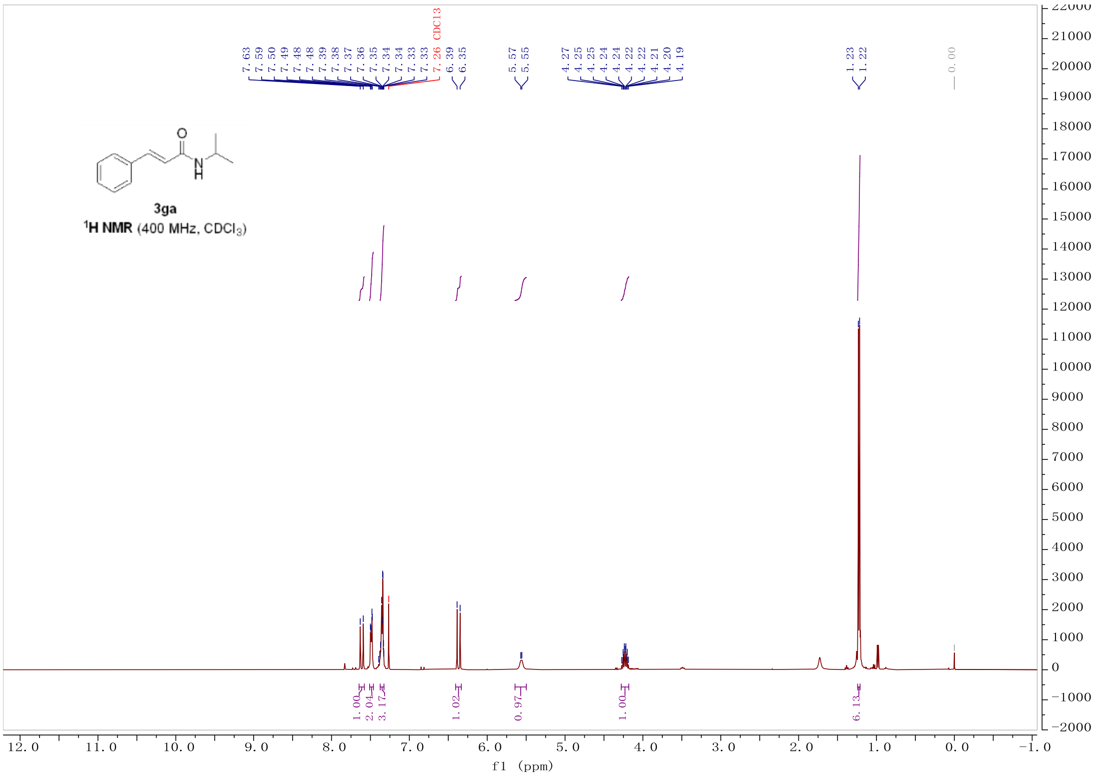
7.63
7.59
7.50
7.49
7.48
7.48
7.39
7.38
7.37
7.36
7.35
7.34
7.34
7.33
7.33
7.26 CDCl3
6.39
6.35
5.57
5.55
4.27
4.25
4.25
4.24
4.24
4.22
4.22
4.21
4.20
4.19
1.23
1.22
0.00
3ga
1H NMR (400 MHz, CDCl3)
1.00
2.04
3.17
1.02
0.97
1.00
6.13
f1 (ppm)

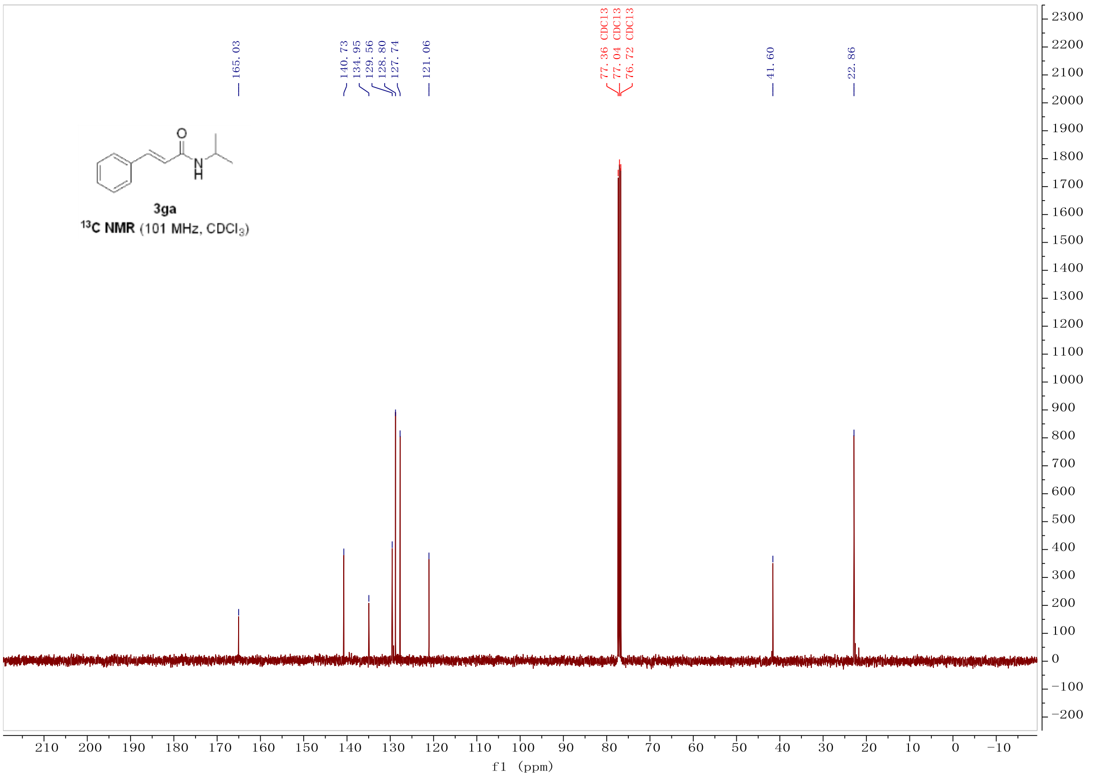
165.03
140.73
134.95
129.56
128.80
127.74
121.06
77.36 CDCl3
77.04 CDCl3
76.72 CDCl3
41.60
22.86
3ga
13C NMR (101 MHz, CDCl3)
f1 (ppm)

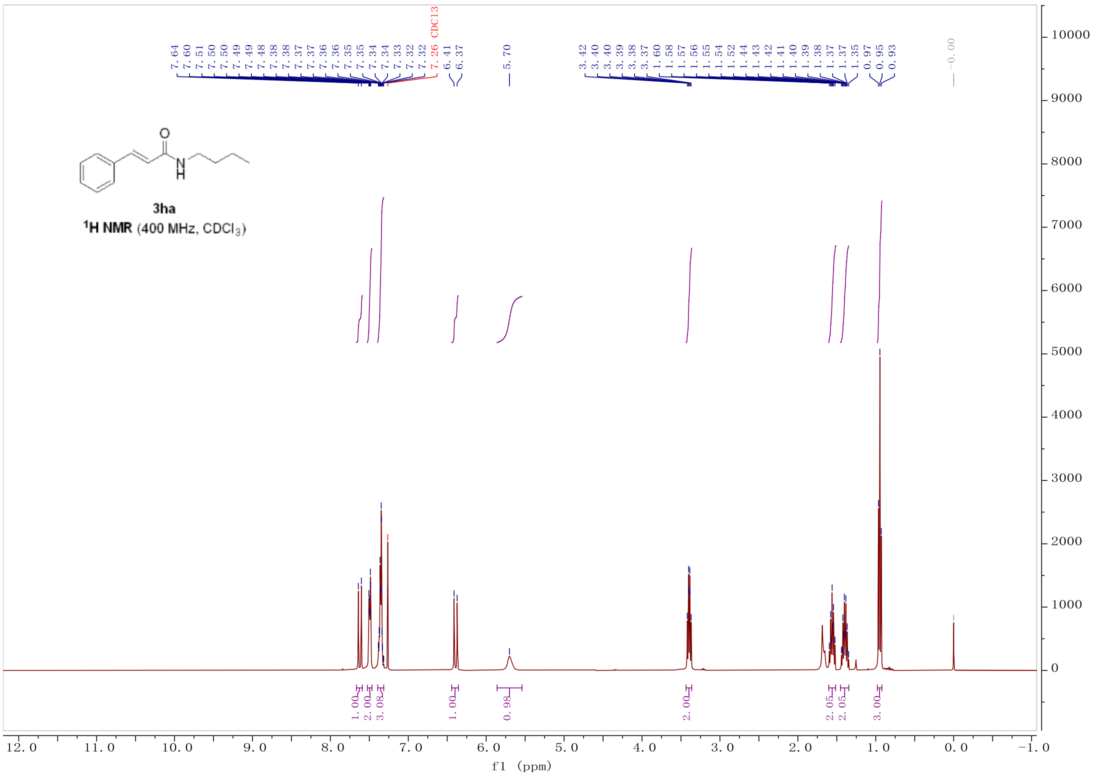
3ha
^{1}H NMR (400 MHz, $CDCl_3$)
7.64 7.60 7.51 7.50 7.50 7.49 7.49 7.48 7.38 7.38 7.37 7.37 7.36 7.36 7.35 7.35 7.34 7.34 7.33 7.32 7.32 7.26 CDCl3 6.41 6.37 5.70 3.42 3.40 3.40 3.39 3.38 3.37 1.60 1.58 1.57 1.56 1.55 1.54 1.52 1.44 1.43 1.42 1.41 1.40 1.39 1.38 1.37 1.35 0.97 0.95 0.93 -0.00
1.00 2.00 3.08 1.00 0.98 2.00 2.05 2.05 3.00
f1 (ppm)

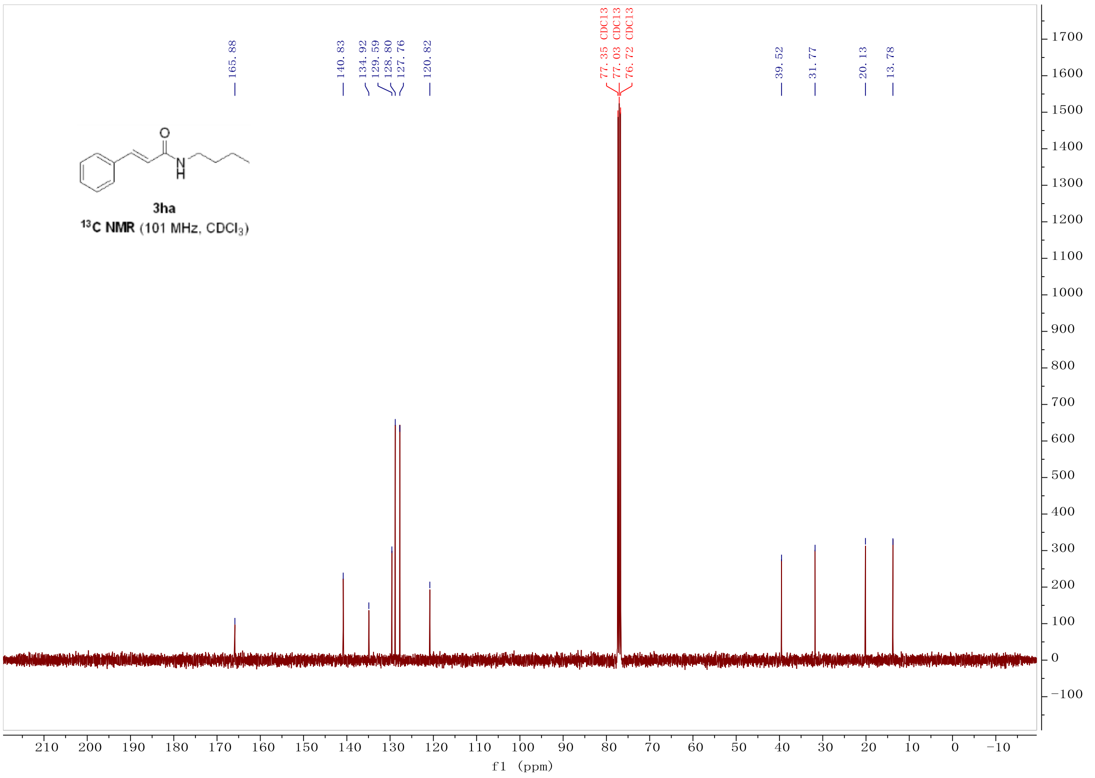
3ha
^{13}C NMR (101 MHz, $CDCl_3$)
165.88 140.83 134.92 129.59 128.80 127.76 120.82 77.35 CDCl3 77.03 CDCl3 76.72 CDCl3 39.52 31.77 20.13 13.78
f1 (ppm)

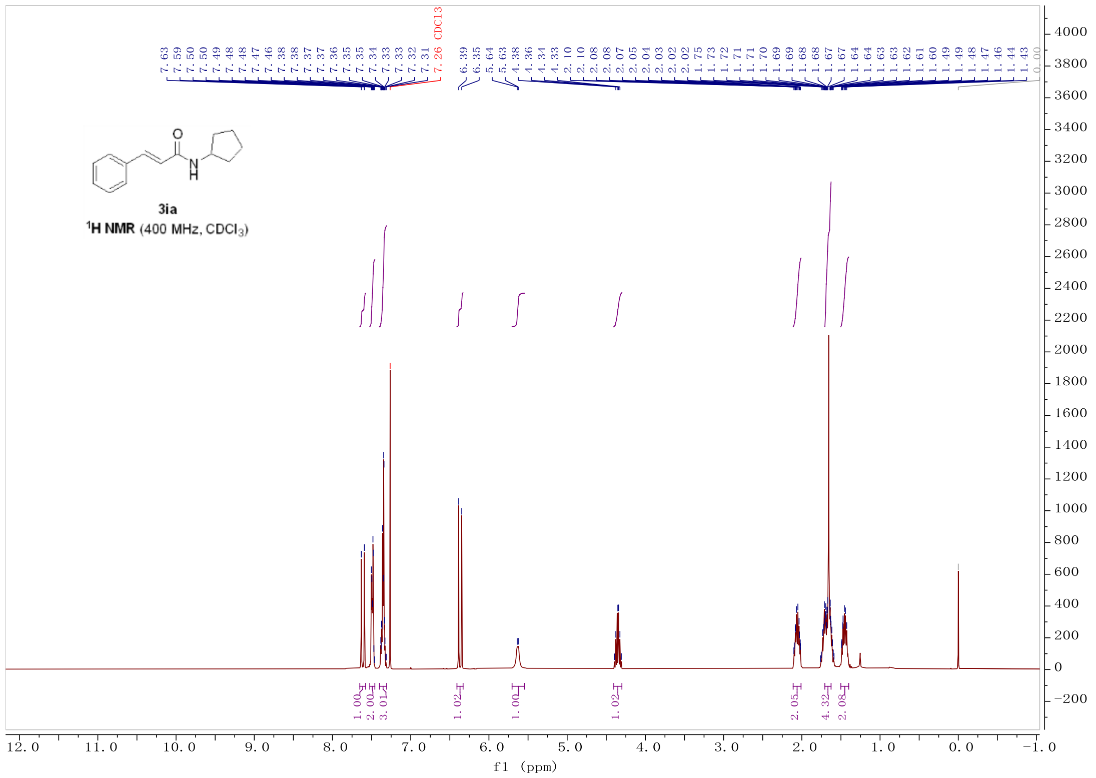
7.63 7.59 7.50 7.49 7.48 7.48 7.47 7.46 7.38 7.38 7.37 7.37 7.36 7.35 7.35 7.34 7.33 7.33 7.32 7.31 7.26 CDCl3
6.39 6.35 5.64 5.63 4.38 4.36 4.34 4.33 2.10 2.10 2.08 2.08 2.07 2.05 2.04 2.03 2.02 2.02 1.75 1.73 1.72 1.71 1.71 1.70 1.69 1.69 1.68 1.68 1.67 1.67 1.64 1.63 1.63 1.62 1.61 1.60 1.49 1.48 1.47 1.46 1.44 1.43 0.00
3ia
1H NMR (400 MHz, CDCl3)
1.00 2.00 3.01 1.02 1.00 1.02 2.05 4.32 2.08
f1 (ppm)

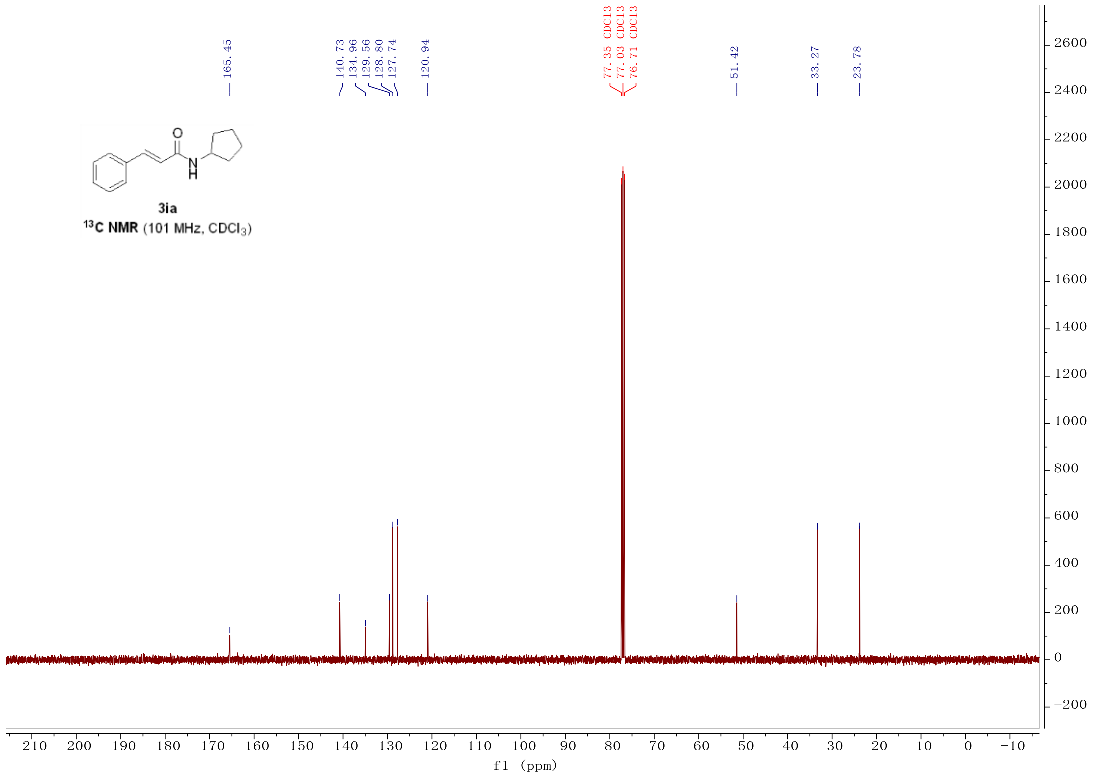
165.45
140.73 134.96 129.56 128.80 127.74
120.94
77.35 CDCl3 77.03 CDCl3 76.71 CDCl3
51.42
33.27
23.78
3ia
13C NMR (101 MHz, CDCl3)
f1 (ppm)
